# Novel Automated Blood Separations Validate Whole Cell Biomarkers

**DOI:** 10.1371/journal.pone.0022430

**Published:** 2011-07-22

**Authors:** Douglas E. Burger, Limei Wang, Liqin Ban, Yoshiaki Okubo, Willem M. Kühtreiber, Ashley K. Leichliter, Denise L. Faustman

**Affiliations:** Immunobiology Laboratories, Department of Medicine, Massachusetts General Hospital and Harvard Medical School, Charlestown, Massaschusetts, United States of America; RMIT University, Australia

## Abstract

**Background:**

Progress in clinical trials in infectious disease, autoimmunity, and cancer is stymied by a dearth of successful whole cell biomarkers for peripheral blood lymphocytes (PBLs). Successful biomarkers could help to track drug effects at early time points in clinical trials to prevent costly trial failures late in development. One major obstacle is the inaccuracy of Ficoll density centrifugation, the decades-old method of separating PBLs from the abundant red blood cells (RBCs) of fresh blood samples.

**Methods and Findings:**

To replace the Ficoll method, we developed and studied a novel blood-based magnetic separation method. The magnetic method strikingly surpassed Ficoll in viability, purity and yield of PBLs. To reduce labor, we developed an automated platform and compared two magnet configurations for cell separations. These more accurate and labor-saving magnet configurations allowed the lymphocytes to be tested in bioassays for rare antigen-specific T cells. The automated method succeeded at identifying 79% of patients with the rare PBLs of interest as compared with Ficoll's uniform failure. We validated improved upfront blood processing and show accurate detection of rare antigen-specific lymphocytes.

**Conclusions:**

Improving, automating and standardizing lymphocyte detections from whole blood may facilitate development of new cell-based biomarkers for human diseases. Improved upfront blood processes may lead to broad improvements in monitoring early trial outcome measurements in human clinical trials.

## Introduction

The lack of biomarkers is one of the foremost obstacles to advances in clinical medicine [1]–[3]. To our knowledge, there are few accurate and highly sensitive blood-based biomarkers for whole white blood cells, i.e., peripheral blood lymphocytes (PBLs). PBLs refer to a broad class of white blood cells including T lymphocytes (T cells), B lymphocytes (B cells), and monocytes. Given that many PBL subtypes are exceedingly rare in blood specimens (<1×10^−6^), yet exert disproportionately large roles in disease, biomarker innovation requires developing an accurate method of separating PBLs from the very abundant red blood cells (RBCs).

PBL separations and thus biomarker standardizations are difficult to develop partly because of blood's high viscosity and its high ratio of red to white blood cells (700 RBCs to 1 PBL). In our experience we have been unable to reliably obtain PBLs of a specific subtype with the decades-old and labor-intensive method of Ficoll density centrifugation for separating blood components. With Ficoll, the PBLs and their subpopulations that we seek to separate are lost in the separation process and the remaining cells that are retrieved are poor in viability, purity, and yield. Accurate methods of separating the many pathologic PBL subpopulations are central to achieving advances in autoimmunity, infectious disease, and cancer.

Although contributing to many diseases, pathological T and B cells are known to cause autoimmune diseases of at least 50 types. In our research on autoimmune type I diabetes, we seek to isolate rare and cytotoxic T lymphocytes (T cells) that bear the cell surface protein CD8. In type I diabetes, the sparse population of pathological, autoreactive CD8 T cells are largely responsible for destroying the insulin-secreting pancreatic islets of Langerhans. These T cells account for only 0.6–2% of the total CD8 T cell population. While some newer forms of centrifugation gradient technology improve whole cell detection by 300-fold [4], [5] they still are not sensitive enough to detect the pathological CD8 T cells whose amounts in human blood are orders of magnitude lower. This same issue plagues others looking for rare antigen activated T cells or pathogenic cells such as ongoing trials in AIDS, cancer, infectious diseases and allergy. High clinical development costs resulting in Phase III trial failures have become commonplace in the AIDS and diabetes literature [6]. There may be a role in improving drug discovery success rates if whole cell biomarkers of rare lymphocyte cell populations could be tracked early to show the negative consequences of an ineffective drug or inadequate dose of a vaccine.

Aiming to replace Ficoll gradient technology, we describe here our novel method of isolating, automating and standardizing the isolation of whole viable cells from fresh blood. Our method incorporates magnetic technology to separate intact PBL subsets from fresh blood samples without gradients and lysis buffers. We provide data to determine quantitatively whether the magnetic method is superior to Ficoll density gradients on three parameters: viability, purity, and yield of PBLs. We also seek to demonstrate that these three parameters are central for predicting success of subsequent biomarker assays. Finally, we seek to demonstrate that the magnetic method can succeed in isolating the extremely rare subset of lymphocytes largely responsible for the pathological attack against pancreatic islet cells [7]. This new method of PBL separation could have far-ranging applications for biomarker development not only for autoimmunity and vaccine development, but also for diagnosis and treatment of any immune-related disease, such as cancer, HIV, allergy and infectious diseases.

## Methods

### Subjects

Patients with Type I diabetes as well as non-diabetic control subjects were recruited over a five-year period at the Massachusetts General Hospital. Patients and controls totaling over 500 subjects, were prescreened to ensure disease course and to exclude subjects with potentially interfering medical conditions. All patient and control blood was drawn into BD VacutainerTM tubes (BD, Franklin Lakes, NJ) containing acid citrate and dextrose or EDTA.

### Ethics Statement

The study was approved by the Massachusetts General Hospital Institutional Review Board (IRB Protocol No. 2001P-001379). Written consent was obtained from all blood donors.

### Blood Preparation

For comparison of PBL separation methods, some PBLs were prepared using Ficoll gradients. Ficoll Hypaque (Amersham Biotech. Uppsala, Sweden) gradient centrifugation of fresh human blood followed the manufacturer's protocol. Remaining red blood cells (RBCs) were removed by a 5-minute (on ice) incubation with NH_4_Cl lysing solution (PharMLyse, BD, Franklin Lakes, NJ). PBLs were evaluated for viability, yield and purity immediately after the isolation.

For magnetic separations, direct isolation of human CD8 T cells from fresh human blood were performed using large magnetic beads (Life Technologies, Product No 113-33D), following the manufactures protocol either by manual methods or with the new automation methods described in this paper. Direct magnetic separation methods were perfected to the extent that daily isolated CD8 human T cells were obtained and studied from fresh human blood less than one hour after the blood draw. A key feature of this magnetic cell separation method is that the separated cells are free of attached beads or antibody and therefore, the membranes of the newly isolated pure cells were available for functional assays. These cell separation methods were further standardized and automated, using the Biomek FX robotic platform for achieving improved uniformity of the cell preparations (Beckman Coulter Fullerton, CA) as outlined below.

For the direct isolation of CD8 T cells from fresh blood, antibody labeled with 3.5 µm diameter super-paramagnetic beads (CD8 positive isolation kit (cat. #113.33D, Life Technologies, Carlsbad, CA) were used. Before each experiment, the beads were washed once with PBS buffer with 1% FBS to remove the sodium azide stabilizing agent from the bead buffer. The beads were then suspended in an equivalent volume of PBS buffer with 1% FBS and placed into a 2 ml sample vial.

The volume of each subjects' whole blood sample drawn was recorded and used later for CD8 cell concentration determination. Following a wash step, the blood samples were re-suspended to 15 ml volume with HBSS buffer in 50 ml (or 15 ml) conical tubes and placed in a custom tube holder on the instrument for processing and transfer to the 2 ml sample vials. Following attachment and CD8 cell isolation, the beads were detached from the cells using the detach-a-bead reagent supplied in the CD8 isolation kit. This reagent consists of a polyclonal antibody directed against the antigen recognition site of the CD8 antibody coated on the magnetic beads. This reagent detaches the antibody/bead complex from the cells by means of competition for the CD8 antibody binding site, essentially leaving a virgin cell. The entire isolation process was performed on ice except for the steps of removing the attached beads.

### Flow cytometry

All lymphocyte cell isolations were analyzed by flow cytometry using a FACSCalibur (BD Biosciences, San Jose, CA). Cell quality was determined by quantifying cell viability, purity and yield. For most flow cytometry studies, flow gates were set “open” for inclusion of all cells. The open gate included cells of all sizes but excluded cell debris, red blood cells, fragmented cells, and apoptotic bodies. The open gate was chosen because cells undergoing cell death, especially by apoptosis, can be detected based on changes in light scattering properties and cell size.

Prior to flow analysis, sample volumes were recorded and a small aliquot (10 µL) from each patient and control sample was removed and the cells counted using a hemocytometer with a microscope. Concomitantly, to help maintain viability, cells were re-suspended in RPMI medium supplemented with 1% FCS and antibiotics following a single 5 min wash by spinning, at ∼600 g at 4°C. After the supernatant was carefully removed a fresh volume of media was added to give ∼1×10^6^ cells per ml, based on the hemocytometer cell count and the original sample volume. Next, a sample aliquot (50 µL) was transferred to a Falcon tube (BD Biosciences) containing 100 µL PI stain solution (5 µg/ml). Subsequently, FITC-CD8 antibody was added and after a 15 minute incubation in the dark and at room temp, the samples were run on the flow cytometer.

We used propidium iodide (PI) to indicate dead cells, and FITC conjugated to CD8 antibody for identifying CD8 T-cells. For these studies, gates were set ‘open’ for inclusion of all cells.

### Buffer reagents

For washing of whole blood, whether isolated with the Ficoll methods or magnetic separation methods, we used 1x PBS or HBSS, without calcium & magnesium chloride, supplemented with 1 or 2% heat inactivated fetal calf serum (FCS) and 1% antibiotics (penicillin/streptomycin and kanamycin Sulfate). For washing the bead-cell complexes following their attachment to the beads, we used Hanks Balanced Salt Solution (HBSS) supplemented with 1 or 2% heat inactivated fetal calf serum (FCS) further supplemented with antibiotics as above. Following the detachment of beads the CD8 T-cells are ready for final suspension in RPMI-1640 media supplemented with 1–2% FCS and 1% antibiotics and flow analysis.

### Cell Death Measurements and detection of rare autoreactive T cells

For measurement of T-cell death, Propidium Iodide (PI) (Oncogene, San Diego, CA) staining was used in combination with AnnexinV. Death was labeled as early (AnnexinV^+^PI^-^) or late (AnnexinV^+^PI^+^) apoptosis or necrosis (AnnexinV^-^PI^+^). T-cell subsets were identified using anti-CD3 antibody (CD4/8-T cells) (Clone UEHT1; BD Pharmingen, San Diego, California). This subset-specific antibody was linked to phycoerythrin (PE).

For the detection of the rare autoreactive T cells of type 1 diabetes, we used two methods. Autoreactive T cells for diverse human diseases undergo accelerated apoptosis when exposed to TNF unlike normal human T cells that show a small amount of proliferation [7]. A 96 well plate based, WST-1 assay (Roche Applied Science, Indianapolis, IN), was used to confirm cell death versus viability. This is a cell proliferation assay that indirectly measures cell death. The isolated CD8 T lymphocytes were plated into 96 well U-bottom plate at a cell concentration of 100,000 cells/well. Cells were cultured overnight at 26°C in RPMI media with 1% heat inactivated fetal calf serum. T cells were then treated with TNF for 1 hr. After 1 hour of exposure to TNF, the WST-1 reagent (Roche Applied Science, Indianapolis, IN) was added according to the manufacturers instructions. The cleavage of WST-1, by metabolically active cells, was measured by a DTX 880 Spectrophotometer (Beckman Coulter, Fullerton, CA) at a wavelength of 405 nm. Each experiment was performed in triplicate. Test medium was used as background control. Data were presented as a percent of proliferation compared to untreated cells. Throughout this text we use the words pass and fail with respect to this assay and this will mean that if killing is observed in a diabetic sample it passes the test.

### Detection of autoreactive CD8 T cells using Tetramers

CD8 T cells were isolated using magnetic bead separation from fresh human blood within 1 hour of blood donation. For isolation buffer we used Hank's Buffered Salt Solution (HBSS, Invitrogen, Grand Island, NY) without calcium and magnesium containing 2% heat-inactivated Fetal Bovine Serum (FBS, Hyclone, Logan, UT) and dasatinib (100 nM, Axon Medchem, Groningen, The Netherlands [8]). Isolated cells were stained with phycoerythrin-labeled HLA-A2 Tetramers containing fragments of the islet specific peptide IGRP (islet-specific glucose-6-phosphatase catalytic subunit-related protein, sequence LNIDLLWSV). For negative controls we used tetramers containing fragments of the breast cancer peptide Her-2/neu (sequence KIFGSLAFL). The IGRP tetramers were produced by the NIH Tetramer Core Facility (Emory University, Atlanta, GA) whereas the Her-2/neu tetramers were purchased from Beckman Coulter (Fullerton, CA). Staining was in the dark for 30 min and the cells were co-stained for FITC-CD8 (Beckman Coulter) during the last 10 minutes. The cells were then washed in HBSS with 2% heat-inactivated FBS and kept on ice until flow cytometry. A minimum of 25,000 CD8 T cells were analyzed on a Becton Dickinson FACSCalibur using the CellQuest acquisition program. Separate samples of cells labeled with FITC-CD8 and Propidium Iodine were analyzed to determine purity and viability for each preparation. The results were analyzed using either no gating, or gating on the main single cell CD8 population, thus excluding debris, dead or apoptotic cells, and clustered cells.

### Robotic Hardware

All experiments were performed using the Biomek FX, a multi-axis Liquid Handler platform (Beckman Coulter, Fullerton, CA) as the platform for the development of the magnetic cell separation process. The instrument we used was equipped with two robotic pods. One was a 96-channel pod with a pair of grippers for moving labware around the instrument work area (deck) in x, y and z-motions. The second robot arm was a span-8 liquid handler. This pod held a series of eight disposable probes (tips) that perform liquid handling operations independent of each other. This arm was used for pipetting, for sample aspirate/dispense operations, for mixing, and when performing blood or reagent transfers. We used 1 ml volume tips (Biomek P1000s, Beckman Coulter, Fullerton, CA) when using whole blood. This was the maximum allowable for the instrument. We used 200 µL tips (AP96 P250's (220 µL) Beckman Coulter, Fullerton, CA) when transferring magnetic beads to blood samples and/or detach-a-bead reagent to blood-cell complexes. Pipetting operations such as aspirating and dispensing of liquids was done at rates of ∼200–300 µL/sec. We also added a mechanical mixing option consisting of an Orbital Shaker (Beckman Coulter, Fullerton, CA). This hardware piece was mounted on the instrument deck and attached as an active automated laboratory positioner (ALP) providing support, power and computer control to allow selectable orbital motions at the 700–800 rpm rate of the shaker. Similarly, a 90 degree rotating ALP was added to the deck in order to provide position orientation control of the sample vial holder. The ability to adjust the sample holder to the best position for span-8 tip access gave the robot arm the best logistics for optimizing the hardware for high throughput.

### Computer and Software

To control the instrument robotic operations, an x486 PC computer was interfaced to the Biomek FX instrument. The computer operates using the Windows XP Professional OS (Microsoft, Redmond, WA). A large number of steps are required to automate the magnetic bead-cell separation process, and many of these steps had to supply liquid level position information supplied as feedback to the software Method in order to keep red blood cell contamination to a minimum during washing steps. We learned through trial and error that the capacitive detection liquid level sensing (LLS) technology supplied with the Biomek FX instrument was unable to provide this accuracy on a consistent basis when doing whole blood samples. This made it necessary for us to write our own computer software program that could address this problem.

### Custom Software

We developed software in-house that successfully addressed the above liquid level accuracy issue by utilizing geometry measurements taken from our custom labware (see below). This information was scripted so that the code could calculate real-time tip positions. This was possible by incorporating a system feature of internal tracking and summing of the number of z-axis stepper motion steps needed. This information was used by the program to calculate precise tip position information. The resulting Method (about 500 lines of code) contained various pre-built software components from both Microsoft OS and the Beckman Coulter Biomek software. We used the Microsoft Visual Studio 6.0 suite of tools (Microsoft, Redmond, WA). The source code is available in the Supporting Information section (Document S1).

The above code was written so it would be transparent by providing an interactive interface module that allowed the user a way to navigate, specify and select the experimental variables used to complete automated cell separation process. All variables are specified by the user at the beginning of the experiment and read in by the software and translated by the Method into specific machine instructions to be completed under control of the computer. This program provides all the instructions necessary to perform the equivalent of what manually would be done at the “bench” when performing the magnetic bead-cell isolation process. This software interface simplifies user interaction with the robot allowing for walk-away operation with automatic alerts, if desired, to the user if there is any cell issue while doing an isolation.

### Sample Dilutions

The large number of red blood cells in whole blood required a sufficient number of washes to achieve a dilution factor of >10^12^. Getting a wash reagent capacity with proper reservoir orientation on the deck for robot access was critical. We used a 40 ml/well by six well reservoir (# 534680 (Beckman Coulter, Fullerton, CA) oriented at a 90 degree placement to allow all span-8 tips equal access. To meet this condition, a custom reservoir mounting support was designed and built to the specifications positioning the reservoir by 90 degrees on a 3 by 1 ALP. This gives the span-8 tips equal access to all reservoir wells simultaneously to speed-up wash steps and minimize potential tip cross-talk occurring when separating different samples.

### Sample vial holder

Keeping with the 96-well micro-titer plate format of the FX instrument, but needing larger ml sample volumes of blood when isolating rare lymphocyte cell subsets, we designed, built and tested a custom sample vial holder (Document S2). Unlike the 96-well plate which has many small fixed wells in a standard footprint, we kept the footprint but redesigned the plate body to enable us to use removable 2 ml sample vials (Corning Inc. # 430661 round bottom cryogenic Nunc vials, Corning Inc, Corning, NY). The 2 ml vials are placed into the custom holder which now has a 4-row by 6-column format. One row of 2 ml (sample) vials constitutes one blood sample and contains a total of 12 ml. There are 24×2 ml potential removable vials (wells) and a possible four blood samples per custom sample holder. The holder was made from white polypropylene plastic set to standard titer plate footprint dimensions. The 2 ml removable sample vials are designed to be discarded at the end of each experiment and the sample vial holder reused.

The sample vial holder was designed to be compatible with a custom magnet plate positioned and underneath the vial holder to allow small magnets to capture the magnetic beads or bead-cell complexes. The sample vial holder design included machined slots to accommodate pick-up and movement of the holder by the span-8 gripper on the instrument deck.

### Ring magnet(s) and magnet base plate

Neodymium iron boron (NdFeB) rare earth ring magnets (750″ OD x .5″ ID x 5 mm), grade N50 (K&J Magnetics, Inc., Jamison, PA) were used to separate the cells of interest from whole blood on the Biomek FX instrument. The symmetrical ring magnet geometry and their several thousand Gauss field strength each make them ideal candidates to use for magnetic bead separation applications as they quickly and easily separate such beads present in 2 ml vials of whole blood.

The two piece custom magnet plate design used to hold the ring magnets in place consists of a magnet support matrix to place each magnet made from aluminum stock, and a base piece made from aluminum alloy with high permeability for magnetic fields (Document S2). This combination minimizes field interference by magnetic field lines under the sample vials. The magnet base assembly is capable of supporting twenty-four rare earth ring magnets, one for each sample vial. In operation, the robot moves the sample vial holder to location over the ring magnets and lowers the sample vial holder onto the magnet base plate bringing the sample vials into the ring magnet center(s).

To help maintain uniform field patterns for the sample vials or positions, the magnet base plate remains populated with the full complement of 24-magnets. These ring magnets can remain in position in the plate, even if using fewer than the four possible samples in the sample vial. Additionally, to help keep external magnet fields in check, each ring magnet is placed into the magnet support holder using an alternating north-south pole facing configuration.

When in position, the magnetic field pulls the magnetically tagged cells in the sample vial(s) into a continuous ring around the lower inside of each vial. This allows pipetting of liquid by the span-8 without disturbing the tagged cells. The ring position is low enough in the vial bottom to allow use of both small 200 µL and larger ml volume washes.

### Two stage magnet post rack

Initial magnetic bead and cell separation experiments began with a two stage magnet post (tube) rack (Beckman Coulter, Fullerton, CA). This magnet tube holder combination consists of a 15 magnetic position holder arranged on a standard plate footprint. The 15 cylindrical (post) magnets are made to be inserted into matching positions on the underside of the top-piece custom MagTubeRack holder. The top labware piece contains 24×2 ml vial positions arranged as 4-rows by 6-columns), of 2 ml tubes (Corning, Corning Inc, Corning, NY). Unlike the ring magnet configuration, each sample vial for the two stage post magnet sits adjacent to one or more post magnets, and thus experiences magnetic interference. This design uses the same number and kind of sample vials used for the custom ring magnet sample vial plate.

### Sample Delivery with Blood Tube Holder

Because blood is drawn in standard 10 ml BD Vacutainer tubes (BD, Franklin Lakes, NJ), dispensing these volumes for delivery by the robot on the instrument using 2 ml vials requires some means to properly allocate the sample(s). To automate the process the span-8 robot arm needs to gain access to the original starting volume of whole blood so it can transfer the sample onto the deck and into the 2 ml sample vials. We accomplished this by designing and building a custom tube holder for standard sterile Nunc vials. This holder was ALP mountable on the instrument deck and accessible by the span-8 so it could do liquid transfer operations into the 2 ml sample vials. The tube holder was designed to use 50 ml or 15 ml conical tubes (Falcon tubes, Becton Dickinson, Franklin Lakes, NJ). The blood is placed into the conical tubes following washing (under a laminar hood to maintain sterility) and loaded into either 50 ml or 15 ml conical tubes. These are placed into the custom holders on the robotic platform on the instrument deck then automatically transferred into 2 ml sample.

## Results

With the goal of developing viable whole cell PBL biomarkers, we first sought to create a cell separation method superior to Ficoll density gradients: we developed and tested a new method of magnetic separation. We also sought to ensure that the new method was accurate and sensitive enough to detect PBL subtypes of special interest to our laboratory. Once we developed the manual version of this new method, we sought to automate it by developing liquid handling software and hardware with a distinctive type of magnet configuration to isolate the cells of interest. We also performed bioassays to determine whether the magnetic method was capable of reproducibly detecting the rare pathological (autoreactive) subset of CD8 T cells, which are largely responsible for autoimmune attack in type 1 diabetes. These final experiments were undertaken to demonstrate that magnetic methods of blood cell separation that accurately detect and quantify PBLs and subpopulations of interest can lead to new standard biomarkers using viable whole cells.

### Comparison of Manual Ficoll Gradient Separation vs. Manual Magnetic Separation

**Figure 1:**
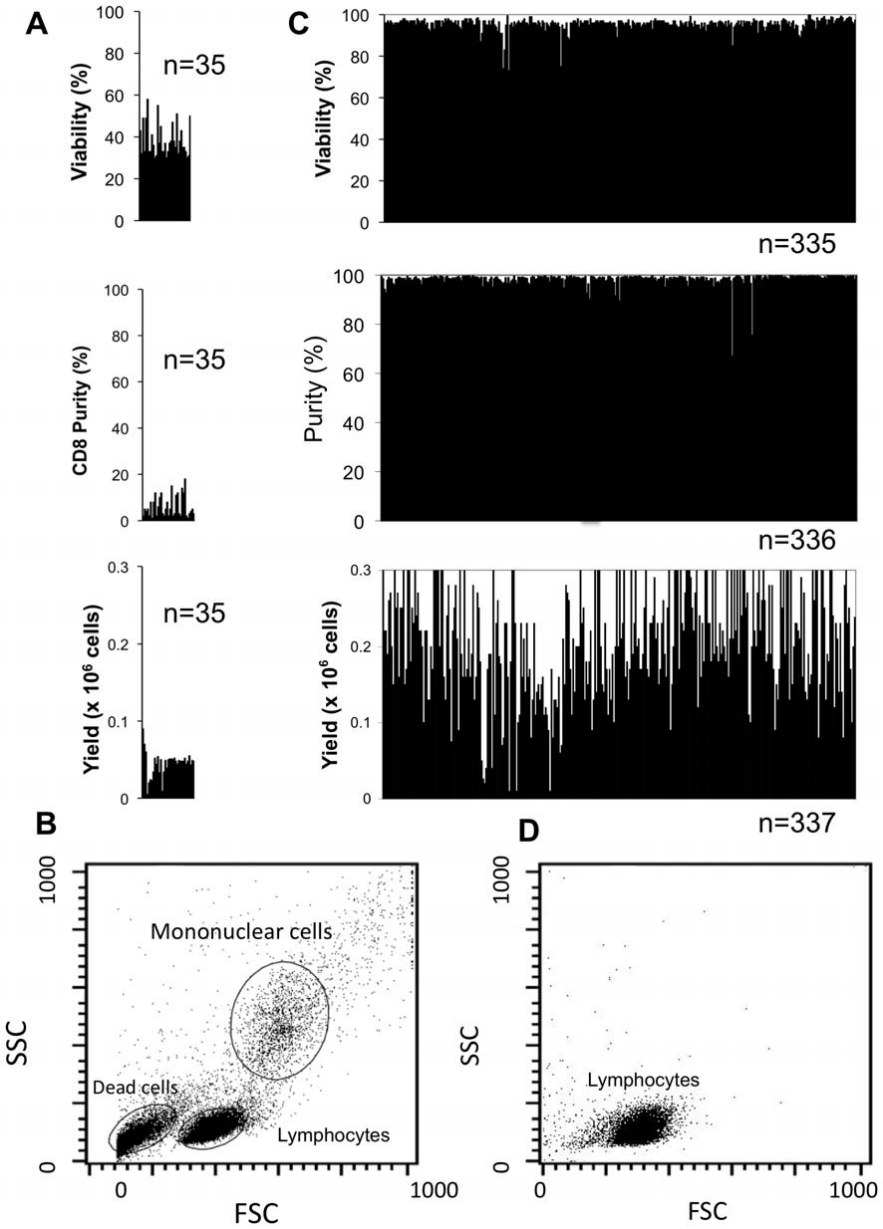
Comparison of accuracy of isolation. In recruited subjects, the accuracy of PBLs or lymphocyte subset (CD8 lymphocytes) isolations from fresh blood was compared between the Ficoll method (N>35 subjects) (A,B) and the manual magnetic method (N>335 subjects) (C,D). Accuracy of the isolates was analyzed by viability (A,C), purity (A,C), yield (A,C), and cellular composition by analysis of FSC (forward)/SSC (side) scatter (B,D) from flow cytometry. The Ficoll method (A) was inferior to the magnetic method on viability and purity (C). The magnetic method (C) also generated higher yields but the variability was high, reflecting inherent variability found across healthy subjects. In addition, the cellular composition, as determined by scatter plots (B,D), revealed that the magnetic method isolated the cells of interest, CD8 T cells, whereas Ficoll produced a mix of PBLs sufficiently contaminated with dead cells and mononuclear cells that it would require further purification to detect autoreactive CD8 T cells.

We first compared the accuracy of blood separations by Ficoll density gradient versus the new magnetic method. Three parameters were studied: cell viability, purity, and yield, each of which was quantified by flow cytometry. We also examined the cellular composition of the isolate, another measure of purity (Fig. 1). Each line in Figure 1a and Figure 1c represents one healthy subject's blood sample studied on a single day.

With the Ficoll method (N = 35 subjects), the viability of PBLs in each individual sample ranged from 40 to 60% viable of the isolated lymphocytes (Fig. 1A), as determined by counting cells stained with propidium iodide (PI), which stains only dead cells. This finding indicates that up to a maximum of 60% of cells in the isolate were already dead immediately after harvest from the fresh blood. As shown by flow cytometry studies on single isolated blood PBL samples after Ficoll, the subjects' PBLs had abundant dead PBLs, and, worse yet, the dead cells became a potential interfering source of background noise by flow cytometry (Fig. 1B).

Purity (Fig. 1A) was measured by the percentage of CD8 T cells within isolates of PBLs. Due to the lack of cell specificity, purity using Ficoll only reached a high of 20% in normal samples. Purity was further analyzed by the cellular composition of the isolated PBLs by a scatter plot of Forward Scatter (FSC) and Side Scatter (SSC) (Fig. 1B). The cellular constituents were primarily mononuclear cells, dead cells, and lymphocytes, the latter of which consisted of a mix of CD4 and CD8 T lymphocytes and B lymphocytes. The yield, or the total number of whole PBLs per 1 mL of human blood assayed by flow cytometry (Fig. 1A), was a mean 0.045±0.003 (SEM) ×10^6^ (n = 35). With the magnetic method that eliminated the need for gradients such as Ficoll for blood separations and without any use of a lysis buffer (Fig. 1C, D), the findings were robust. The presented Ficoll data had demonstrated CD8 cells at low levels of purity (Fig. 1A). Using the new magnetic methods applied to fresh blood, we found striking improvements over Ficoll for all three parameters (Fig. 1C, D). The viability of CD8 T cells was very high and averaged 98.2±0.3% (n = 335). The mean purity of CD8 cells obtained by flow cytometry was 90.8±0.5 (n = 334). The purity of the sample was bolstered and self evident by the analysis of the scatter plot (Fig. 1D). The plot of FSC vs. SSC showed an abundance of intact, normal CD8 T cells, negligible percentages of dead cells, and absence of monocytes. The yield, or the total number of whole CD8 cells per 1 mL of human blood, was 0.23±0.01×10^6^ (n = 326), a value more than five-fold greater than the yield with Ficoll.

### Development and design of automated magnetic lymphocyte separations for fresh human blood

The goal of this project was to demonstrate the accuracy of magnetic separation methods in terms of viability, purity and yield of cells. It was our belief that these manual methods should be automated for finer degrees of accuracy and reproducibility for clinically useful whole cell biomarkers. If successful, the automation process would also increase capacity, accuracy and set the stage for another level of standardizations that would remove the human factor from manually performing the blood separation process. At this time point we were working under the assumption that the standardization of whole blood separations, by the defined parameter of whole cell viability, purity and yield, would also strengthen the accuracy of downstream whole cell biomarker assay perfection.

One of the early decisions in the design of the whole blood cell separation process was to find a paramagnetically tagged antibody reagent that was compatible with robotic whole blood separations and could bind PBLs of interest, for instance CD8 lymphocytes. After comparing a variety of paramagnetically linked antibody methods, we found that antibodies labeled with 3.5 µm diameter super-paramagnetic beads resulted in lymphocyte subpopulations with the best purity, yield and viability. This is a relatively large bead size for the paramagnetic antibody reagent but it was chosen because larger beads expedite cell migration to the side of the tube. Also, large magnetic beads attached to the blood cells of interest were likely to eliminate the problems inherent in the use of their small counterparts. Small paramagnetic particles attached to antibodies require other blood processing steps such as centrifugation, long time periods for cell separations through viscous blood and complex wash and handling steps due to the longer times to bring a cell to the side of the tube. Also, larger magnetic forces would need to be applied to smaller particles. In addition it was generally observed that large magnetically linked antibodies require the binding of fewer cell surface epitopes to facilitate cell movements to the side of the tube.

**Figure 2:**
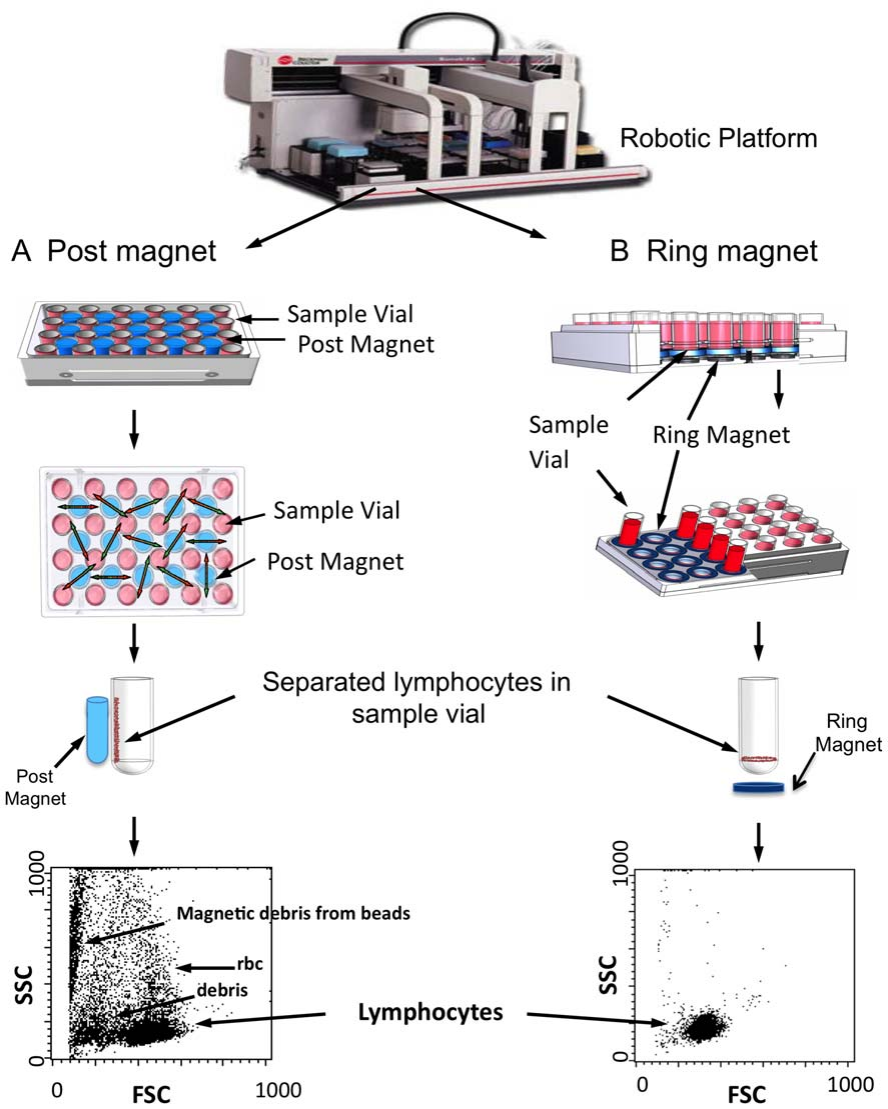
Automated magnetic separations of lymphocytes from human blood using a robotic platform. We compared two different plate designs for the robotic platform (A,B). For each test system, Nunc vials were loaded with fresh human blood and paramagnetic beads tagged with the antibody to the whole blood cells of interest was added to the sterile vials. This blood and antibody mixture typically was tested for its ability to separate lymphocyte subsets, such as CD8 or CD4 white blood cells. The most robust platform design gradually culminated in two different plate designs with varying configurations of the external magnets. On the left (A) is a plate design with a post magnet on the outside of the Nunc vial. On the right (B) is a plate design with a ring magnet design at the base of the Nunc vial. The resulting separated lymphocytes were evaluated for the highest yield, viability and purity. It was first thought that a full-length magnet along the side of the tube would facilitate the cellular separations. However, the disadvantage of this plate design was that every magnet experienced interference from the surrounding magnets, thus decreasing the overall density of Nunc vials for maximal efficiency and/or decreasing the magnetic force. As a result, this method produced lymphocyte preparations contaminated with RBCs and magnetic debris, as shown in the left scatter plot of the yielded cells. In contrast, the ring magnets in (B) produced internally consistent magnetic fields that did not interfere with adjacent ring magnets since the pulling force was exclusively directed to the interior of the vial. Also, the ring magnet configuration maximizes the plate capacity for the maximal number of Nunc vials per plate, and allowed the isolation of CD8 lymphocytes from whole blood with much higher purity (right scatter plot).

Using this large bead size for the paramagnetic system, we built a robotic workbench configured as a platform to integrate hardware and software for automating lymphocyte isolations from fresh blood specimens. Combining paramagnetic bead technology combined with the design of external magnets, we tested and built the platform for its ability to separate whole cell lymphocytes, typically CD8 lymphocytes. The purpose and goal of the robotic system was to build a platform that did not require centrifugation steps that are difficult to automate and also to avoid any whole blood lysis steps requiring reagents that might facilitate removal of contaminating RBCs, but would also decreased the viability and yields of the lymphocytes. The workbench liquid handling and robot motions were controlled using custom software to specify position, movement, and mixing of blood samples held by lab ware built to function within the designated instrument deck area. We worked within the robotic platform constraints to engineer, build and test different magnet geometries, magnet holders, and mounting plate assemblies made to support sample plates typically holding 2.0 ml sterile cryo vials also known commonly as Nunc vials. We then took the best working magnet configurations (Fig. 2), sample tube plate designs and liquid processing requirements to seamlessly integrate each hardware piece with the automation movements (See Methods and Document S2).

Figure 2 shows the two most promising of several designs for the platform sample holders. Depicted on the left side is an automated robotic platform based on cylindrical (post-shaped) magnets, whereas the right side of the figure shows a design based on ring shaped external magnets that apply forces to the antibody linked cells with magnetic bead particles. FSC/SSC flow cytometry showed that the ring magnet design produced the purest cells of interest, in this case a subpopulation of white blood cells lymphocytes, called CD8 T cells. Impurities in the yielded cells with post-shaped magnets presumably were due to interference among the magnetic fields of adjacent cylindrical magnets. This was avoided by using ring magnets embedded in a flat plate where interfering fields between magnets were not an issue and overall capacity increase was possible by placing the magnets and Nunc tubes closer together.

### Isolation of rare blood cell populations by magnetic separation: impact on biomarker assays

**Figure 3:**
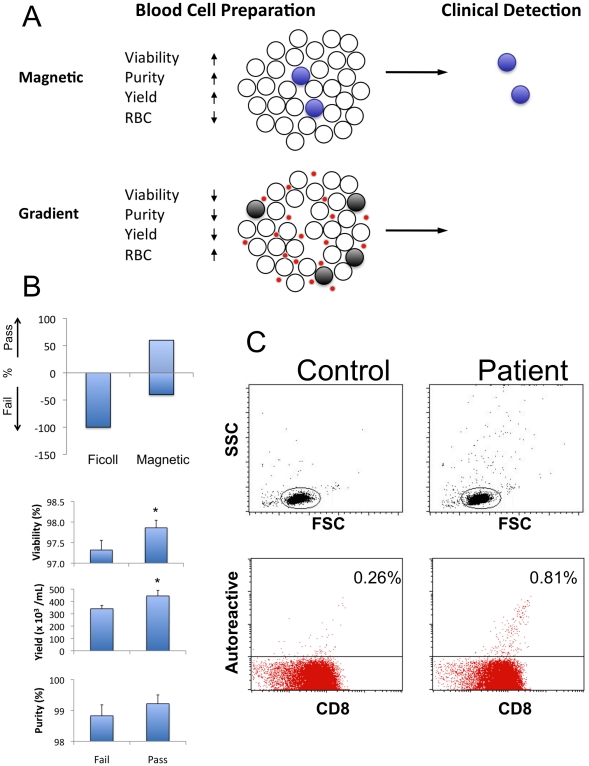
Lymphocytes for biomarker assays from Ficoll versus a magnetic method. In order to detect rare lymphocyte populations, it is critical that the separation method yields healthy cells of sufficiently high purity. (A) Schematic depiction of magnetic and gradient methods. The magnetic method is gentle, highly selective and retains the lymphocytes of interest (blue cells), whereas the gradient method has remaining red blood cells (red dots) and low viability (dark cells), while the cells of interest are largely lost due to cell death. (B) Comparison of performance parameters of magnetic and Ficoll gradient separation methods. Autoreactive CD8 T cells were isolated from 56 blood samples from human type I diabetics with both Ficoll and magnetic methods. Using the insensitive WST biomarker assay of rare autoreactive T cells, only magnetically separated cells showed the presence of the cells (Pass); Ficoll isolated cells uniformly lacked autoreactive T cells. The accuracy of the cell separation method was established by determining viability, purity, and yield. The results show that extremely efficient cell isolation procedures with high yield and high viabilities are required to preserve the cells of interest. (C) Flow cytometry with tetramers specific for autoreactive CD8 T cells shows that these cells are present and detectable after separation from patient blood samples using our magnetic method.

Taken together, the findings from >335 fresh blood samples studied by the magnetic method (Fig. 1C), combined with the ability to automate this blood separation process with robotic platforms (Fig. 2B), were convincing enough to justify the examination of these separated cell preparations in biomarker assays. We were particularly interested in testing the isolated whole blood cells for the reproducible detection of rare CD8 T cell subsets (approximately 0.6–2% of the total cell population) that are the autoreactive T cells in type I diabetes. As Figure 3A depicts, we hypothesized that with whole blood derived cell preparations featuring high viability, purity, yield and only infrequent RBC contamination, it was timely to see if the rare cells of autoimmunity persisted and were detectable. For this initial biomarker testing, autoreactive T cells were first studied by an indirect method of exposing isolated CD8 T cells to Tumor Necrosis Factor (TNF), which selectively kills autoreactive, but not normal CD8 T cells, which continue to proliferate [7]. After TNF challenge, the remaining CD8 T cell isolate was assessed for autoreactive cells by the WST assay for T cell proliferation. The second biomarker method we used to assess the impact of the isolation method on the detection of lymphocyte subpopulations was the more highly sensitive “tetramer” method. This highly sensitive method of autoreactive T cell detection directly evaluates cells for the presence of rare autoreactive CD8 T cells, and involves the incubation of whole blood isolates with self peptide bound with fluorescent HLA class I protein. Only a single clone of autoreactive T cells should recognize the tetramers in flow cytometric studies.

We performed a functional biomarker assay examining blood samples from 56 pairs of type I diabetics and matched non-diabetic controls to determine whether the automated magnetic method was sensitive enough to detect the presence of rare cells of interest, namely CD8 T cells (Fig. 3A,B). Using a relatively insensitive assay for autoreactive T cells, we found that 79% (n = 44/56) of diabetic blood samples isolated by the automated magnetic method were correctly identified as having the rare autoreactive cell type, i.e., the “pass group”. In marked contrast, none of the samples from the same 56 diabetic patients tested by Ficoll was found positive, i.e., the “fail group”. In other words, the prevailing method of Ficoll separation had a 100% failure rate.

A “pass” rate of 79% with the WST assay after the magnetic method is excellent compared to the Ficoll method with 0% success. However, it is not ideal and does not answer the question whether all improved methods of cell separations are adequate for this relatively insensitive biomarker assay. We therefore more finely studied the whole cell isolates to see if subtle differences in the cell isolation methods, as quantified by viability, purity and yield, might impact the rare cell detection with the WST assay. This was important from a clinical trial viewpoint, since if some parameters of monitoring autoreactive blood cell separations could have quality control cut offs, inadequate quality preparations of cells would not be studied further in biomarker assays. Therefore, we conducted an analysis (Fig. 3C) of only the magnet samples that passed (the 79% of patients with true positives) to determine which of the blood separation parameters—viability, yield, or purity—was most determinative for enhancing the pass rate as compared to the 21% of the magnetically separated cells that failed. The results show that better yield and viability are more important than is purity for detecting this rare cell population of autoreactive CD8 T cells. Figure 3C shows that a slight reduction in viability from 97.9±0.2% to 97.3±0.2% (average ± SEM; p = 0.04) makes the difference between passing or failing detection by the WST assay. We infer from this finding that the difference is minor because the population of apoptosis-prone, autoreactive CD8 T cells only accounts for a small segment (0.6%) of the total CD8 T cell population. Thus, these cells can be induced to die very easily. Therefore, the isolation process must be extremely gentle and efficient otherwise these cells will no longer be present in the final isolate.

We also observed a statistically significant difference between yield in the “pass” versus “fail” group (Fig. 3C, 444,700±44,900 versus 340,800±28,000 CD8 T cells per mL of donor blood; p = 0.03). This was expected because viability and yield are correlated in this type of process. Finally, there also was a trend for higher purity as being important to detection of autoreactive CD8 T cells, but the difference was not significant and would require a much larger sample size to reach sufficient power (Fig. 3C; 99.2±0.29% versus 98.8±0.35%; p = 0.20). The slight difference between passing and failing detection is approximately 0.5% in this relatively insensitive assay for autoreactive CD8 T cells. The analysis showed that even the magnetic method of blood separations could be impacted by cell quality and, at least for these samples, perfection of viability, yield, and purity avoided false negatives. Minor quantifiable differences distinguish between detection or non-detection of this rare autoreactive cell population. Put another way, blood separation methods impacted rare T cell detection through biomarkers.

The detection of rare autoreactive T cells isolated from blood could also be impacted by the choice of biomarker assay since different assays can have different degrees of sensitivity. The second biomarker method applied to assess the impact of improved isolation method on the detection of lymphocyte subpopulations was the more sensitive “tetramer” method. This highly sensitive method of autoreactive T cell detection involves the incubation of whole blood cell isolates with self peptide bound with fluorescent HLA class I protein. Only a single clone of autoreactive T cells is recognized, i.e. the rare pathogenic T cells. The published literature had previously reported using Ficoll methods that rare autoreactive T cells could not be found in long term diabetics with Ficoll isolation methods and “tetramer” methods [6]. Using patients who had “failed” in the insensitive WST assay but had separated lymphocytes from magnetic methods, i.e. part of the 21% fail group, we uniformly detected peptide specific autoreactive T cells with the more sensitive “tetramer” assay (Fig. 3D). Thus, refined and automated blood separations using magnetic methods led to successful rare T cell detections. The quality of cell preparations and the sensitivity of the biomarker assay in combination were synergistic in success.

### Automation leads to reduced labor and process costs

**Figure 4:**
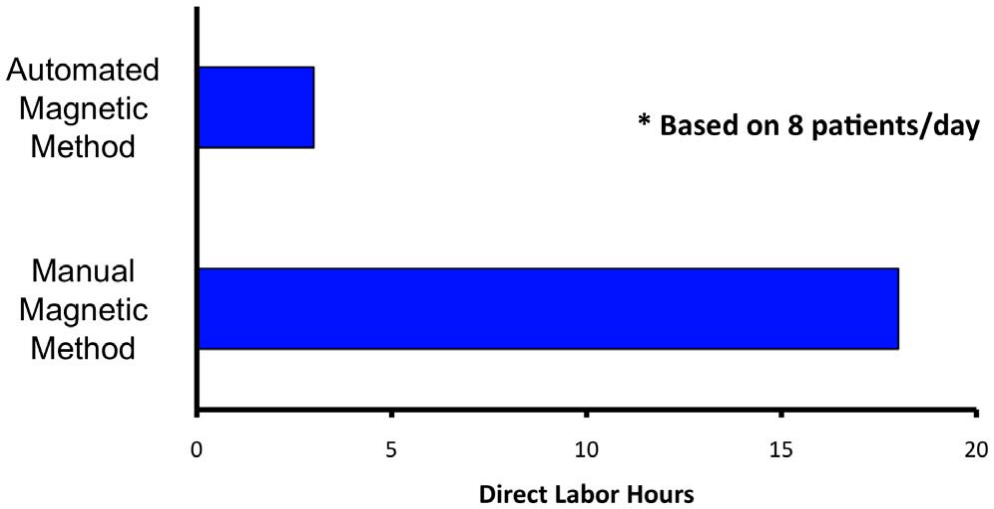
Comparison of labor hours needed to perform manual versus automated CD8 cell separation in our laboratory. Based on our workflow, one research associate works 4.5 hours to process blood samples from one diabetic patient and one control; or a total of 18 hrs for 8 patients. In our current automated setup one associate can initiate the processing of 8 samples in parallel and finish in 3 hrs while being able to perform other activities once setup is completed. Such dramatic savings in amount of direct labor will translate in large cost savings and increased throughput.

In addition to the inaccuracies inherent in the Ficoll isolation methods, the labor cost of the resource-intensive Ficoll separation of blood specimens is also rate-limiting for the development of whole cell lymphocyte isolations. To assess economic impact, we compared the labor time needed for manual PBL cell isolation using the new magnetic methods against the automation methodology described in this paper. We estimated resource needs and an overall estimate of gain in productivity. As is shown in Figure 4, the savings in manpower by automating cell isolations by the magnetic method are significant and will translate in substantial time, personnel, and cost savings. These benefits are on top of improved sample quality and consistency of meaningful data.

## Discussion

Escalating clinical care costs and high translational costs for new drug development are a motivation to develop better biomarkers for human disease. Biomarkers are important tools for clinical development since they provide early signs of drug efficacy, toxicity, dosing and failure. Indeed, much progress has been made in the development of biomarkers based on pharmacogenomics, mRNA and proteomics to track the human response to new drugs in clinical trials. What is lacking in biomarker development is the routine use of human blood whole blood cell markers of disease. The examination of lymphocytes and of the rare antigen-specific lymphocyte subsets is relevant to vaccine and AIDS research, as well as to infectious disease, autoimmunity and cancer trials. A remarkable number of opinion papers estimate that biomarkers on patient populations could lead to cost savings of $80 billion per year in streamlining clinical trials, yet one important facet of biomarker development, viable whole white blood markers, is conspicuously absent [7].

The use of Ficoll for the separation of blood into PBLs and RBCs was introduced in 1968 [9]. Since then it has become the mainstay for the separation of white blood cells and remains the method of choice in many clinical laboratories. Even so, Ficoll gradient separations result in poor quality PBLs combined with low yields and poor viability.

The literature shows that there are potential problems with Ficoll separations, such as contamination with iodine [10], loss or death of specific subpopulations of lymphocytes [11], [12] and changes in expression of adhesion molecules [13]. Also the use of Ficoll has failed to lead to any standardized biomarkers from whole blood nor did it lead to the easy standardization of biomarkers in clinical trials that depend on viable lymphocytes.

In this project we hypothesized that the poor or missing biomarkers based on whole blood lymphocyte preparations is likely due to inadequacies in the upfront process of whole blood cell separations. We reasoned that the decades old methods of whole blood cell separations were perhaps losing the cells of interest or the cells of interest were dying due to the harsh isolation conditions (Fig. 3A). Recent world-wide technology to isolate subpopulations of blood cells has been developed and these methods now frequently utilize paramagnetic particles coated with antibodies against specific cell surface markers to separate the white blood cells of interest. In addition, it is important to note that the methods as presented and further developed here can be performed on whole blood without the need to lyse the red blood cells nor enrich the lymphocytes using pre-processing steps that often involve gradient centrifugations. Our need for improved blood cell isolation methods has led us to the development of the automated magnetic separation methods that are the subject of this paper (Fig. 2).

In general, the isolation of white blood cells from whole blood is difficult and biologically hampered by the fact that the blood contains red blood cells that exceed the number of white blood cells by more than 700 to 1. Separations are further hindered by the viscosity of whole blood, the presence of serum proteins and the large number of cells per ml of blood. Many protocols therefore call for the pre-enrichment of WBCs by means of gradient separations and/or RBC lysis, prior to the application of magnetic bead separation. For rare cell populations this defeats the advantage of using magnetic bead technology as many cells already have been lost or damaged after this pre-enrichment step. This is demonstrated by the low yield after Ficoll gradient separations (compare Figs 1 and 3). For rare cell populations, it is critical that these cells are preserved by the isolation process and remain healthy for subsequent biomarker assays. Based on these considerations we decided to concentrate our efforts on cell separations directly from whole blood without previous lysis or gradient steps.

As a demonstration of the need for the highest possible quality of isolated blood cells to standardize biomarkers from blood, we present data that demonstrate that preparations of whole blood lymphocytes from fresh blood can lead to biomarker success, in contrast to Ficoll blood isolations that result in uniform failures. We show that a WST-1 assay for rare autoimmune cell detection only works reliably when the isolated blood cells are of sufficiently high yield, viability and purity (Fig. 4). The relatively small difference in viability, yield and (to a lesser extent) purity between the “pass” and “fail” groups is presumably due to the fact that these cells only constitute a very small fraction (less than 1%) of the total CD8 T cell population. Since these cells are more apoptosis-prone, they will be under-represented, or missing altogether, in the batch of isolated cells unless the blood separation process is efficient. In further support of the hypothesis that lymphocyte preparations from whole blood can lead to biomarker detection, we observe that automation of blood separations can also be successful.

The processes required to separate blood cells are tedious and time-consuming, especially for Ficoll methods. It usually takes a trained lab worker several hours to generate the necessary isolated cells. In addition, even when performed by the same skilled individual, there are quality and repeatability issues associated with the isolated samples that tend to make it hard to control uniformity and consistency. Variation can be significant; it tends to increase when more than a few samples are run in parallel and tends to be exacerbated due to person-to-person variability. When there is a need to scale-up, as would be the case in the clinic, the need to have greater capacity and/or higher throughput must be addressed. Consistent sample quality, the importance of timing and being able to process samples at all hours makes scheduling complicated. Many of these issues can be avoided by automation of the cell isolation process. The time-savings with the automated method will translate to large cost-savings, especially in a clinical setting, where high throughput is important.

Improving the efficiency of the blood separation process for the implementation of biomarkers represents a major advance in basic science with near-immediate translation to the clinic. The use of biomarkers to identify and test drug candidates may allow an increase in the numbers of new drugs and hopefully new drug approvals and is a current research topic of great interest for speeding the drug approval process with cost efficiencies [14]–[16]. In this paper, we show the impact of changing the basic concept of how fresh human whole blood is handled to obtain viability lymphocytes for biomarker studies. We eliminate both gradient and lysis methods to enrich lymphocytes and showed that the entire process can be automated with magnetic methods of blood cell separations. These innovations not only resulted in marked improvements in the yield, viability and purity of the cells, but also led to the validation of biomarkers assays that have previously failed in human populations. Biomarker data can be extremely valuable as predictors of drug effects. Our data suggests that the automation of whole lymphocyte separation from fresh blood may be central to developing PBL biomarkers aimed at fulfilling the needs of clinical research and translation to the clinic.

## Supporting Information

Document S1 **Blood cell isolation source code for Biomek FX.** To automate the isolation of human CD8 cells from whole blood we developed source code for the Beckman Coulter Biomek FX robotic platform. A user interface was written using Microsoft scripting software and Biomek liquid handler software templates to create a Method that was optimized for control of all CD8 isolation steps. This script uses software palettes and project file information to configure all the mechanical actions and functions of the instrument's robotic motions and to accurately process those instructions as part of the hardware setup and control.
(PDF)

Document S2 **Custom sample tube holders.** We custom designed and built sample holders for 2 ml sample vials (up to 24 vials) and a maximum of four patient samples (one for each row). A sample occupies one-row of the four-row (by 6 column) holder design. Each (sample) row holds six vials to provide sufficient volume (up to 12 ml) to provide a sufficient number of cells of interest to be collected. Shown are several different views…clockwise from top left, are tube holder top view, viewing the tube holder at an angle, and a side view of both the sample plate with two (of 24) vials sitting within the holder and on the magnetic separation plate. The exploded view shows the various engineering components used to fabricate the sample holder base and magnetic separation plate. This plate is used to pull any magnetic beads that are present in the sample vial solutions to the sidewall. The plate design includes the use of 24 separate (rare earth) ring magnets (one ring magnet for each sample). One (2 ml) sample vial is illustrated above the sample holder. The use of multiple plate layers ensures correct ring magnet placement to allow for accurate sample vial x, y, z insertion position. The various cut-away plates also help to confine (isolate) magnetic field lines to minimize unwanted interferences and field line overflow at the bottom of the sample vials. Separately, a sample plate holder is shown (top-right) with inserted sample vial. The slot seen near the top (matching on opposite side) is used by the robotic arm to move the plate on/off the magnet plate. On the bottom right is a cut-away side view of a sample holder sitting on a magnet separation plate with the sample vial positioned in the ring magnet of an assembled magnet separation plate. As shown, the ring magnet is positioned near the bottom of the sample vial which allows use of large and small liquid volumes.
(TIF)
