# Supplementary material for: Novel Automated Blood Separations Validate Whole Cell Biomarkers
Source: PLoS One. 2011 Jul 22;6(7):e22430. doi: 10.1371/journal.pone.0022430 (PMC3142167; doi:10.1371/journal.pone.0022430)
Supplement: Document S1 — Blood cell isolation source code for Biomek FX. To automate the isolation of human CD8 cells from whole blood we developed source code for the Beckman Coulter Biomek FX robotic platform. A user interface was written using Microsoft scripting software and Biomek liquid handler software templates to create a Method that was optimized for control of all CD8 isolation steps. This script uses software palettes and project file information to configure all the mechanical actions and functions of the instrument's robotic motions and to accurately process those instructions as part of the hardware setup and control. (PDF) [file pone.0022430.s001.pdf]

CD8Prep 0.12
6/8/2011 14:13:17
Copyright 2011 Massachusetts General Hospital

Method

Author: Administrator

Description:

Start

Comment

Description:

Version History

Comment:

0.10 10-20-08 OS FillVials & CD8Prep combined (w/real rotating ALP)

Scripted Let

Bitmap: C:\Program Files\Biomek Software\Bitmaps\Vials.bmp

Description:

Caption: Scripted Let

Code: World.Globals.PropertyChanger.ClearGlobals

```
Set World.Volatile.Shell = CreateObject("WScript.Shell")


Extend "MFX",            CreateObject("MGH_FX.CCD8Prep3")

Extend "KW",             CreateObject("OSTools.CKeywords")

Extend "TLS",            CreateObject("OSTools.CTools")

Extend "PC",             World.Globals.PropertyChanger

Extend "PG",             World.Globals.PauseGenerator

Extend "SIM",            CBool(Pipettor.PipettorObject.Channel.Port = "Simulate")

Extend "IsRunning",      Not CreateObject("World.EngineObject").Simulating
```

```
Extend "SNStartBelowCM", 0.6
Extend "ZMinCryo",       0.5


 Extend "RowWells", Array("", "1,2,3,4,5,6","7,8,9,10,11,12", "13,14,15,16,17,18",
"19,20,21,22,23,24")
 Extend "ColWells", Array("", "1,5,9,13,17,21", "2,6,10,14,18,22", "3,7,11,15,19,23",
"4,8,12,16,20,24")
Prompt: [IDispatch]
Tooltip: Configure Step
---------------------------------------------------------------------------
Script
Description:
Demo UI: P=34363, Pod=464, Volume=0
Execute the following script code:

'Make the wash station visible so it can be used in the SN Dispense step
World.LabwareClasses.WashStation.Visible = True

'----------------------------------------------------------------------------
PC.SetGlobal "VolMinSB",                  cint(StepDictionary.Let.minsb)
PC.SetGlobal "VolMaxSB",                 cint(StepDictionary.Let.maxsb)
PC.SetGlobal "MaxVolInTips",            cint(StepDictionary.Let.maxvtip)
PC.SetGlobal "SettleTimeBlood",        cstr(StepDictionary.Let.settletimeblood)
PC.SetGlobal "SettleTime",                cstr(StepDictionary.Let.settletime)
PC.SetGlobal "WorkOffline",            cbool(StepDictionary.Let.wkoffl)
PC.SetGlobal "SendSMS", "/doit=" & cbool(StepDictionary.Let.sndsms)
PC.SetGlobal "TipsUsed", 0
```

```
PC.SetGlobal "Col1", "1,7,13,19"

PC.SetGlobal "Col2", "2,8,14,20"

PC.SetGlobal "Col3", "3,9,15,21"

PC.SetGlobal "Col4", "4,10,16,22"

PC.SetGlobal "Col5", "5,11,17,23"

PC.SetGlobal "Col6", "6,12,18,24"


PC.SetGlobal "Col12", "1,7,13,19,2,8,14,20"

PC.SetGlobal "Col34", "3,9,15,21,4,10,16,22"

PC.SetGlobal "Col56", "5,11,17,23,6,12,18,24"


PC.SetGlobal "Wells", Array("",

"1,2,3,4,5,6","1,7,2,8,3,9,4,10,5,11,6,12","1,7,13,2,8,14,3,9,15,4,10,16,5,11,17,6,12,18",

Col1 & "," & Col2 & "," & Col3 & "," & Col4 & "," & Col5 & "," & Col6)


xCol0 = array("","","","","")

xCol1 = array("","1","1,7","1,7,13","1,7,13,19")

xCol2 = array("","2","2,8","2,8,14","2,8,14,20")

xCol3 = array("","3","3,9","3,9,15","3,9,15,21")

xCol4 = array("","4","4,10","4,10,16","4,10,16,22")

xCol5 = array("","5","5,11","5,11,17","5,11,17,23")

xCol6 = array("","6","6,12","6,12,18","6,12,18,24")


PC.SetGlobal "Col", array(xCol0,xCol1,xCol2,xCol3,xCol4,xCol5,xCol6)


' Tip Patterns - single or pairs

PC.SetGlobal "OneTip",  array("","1","1,2","1,2,3","1,2,3,4")

PC.SetGlobal "TwoTips", array("","1,5","1,2,5,6","1,2,3,5,6,7","1,2,3,4,5,6,7,8")
```

```
'---- Move pods to ZMax --------------

LeftPod.MoveZ LeftPod.Max.Z
On Error Resume Next ' in case there is no RightPod
zm = RightPod.Max.Z-1
ArrZ       = array("z1","z2","z3","z4","z5","z6","z7","z8")
ArrZDisp  = array(zm,zm,zm,zm,zm,zm,zm,zm)
ArrD       = array("d1","d2","d3","d4","d5","d6","d7","d8")
ArrDDisp  = array(0,0,0,0,0,0,0,0)

RightPod.AbsoluteMoveAxes ArrZ, ArrZDisp,RightPod.SpeedLimit
---------------------------------------------------------------------------
Group
---------------------------------------------------------------------------
Define EmptyTipsToWaste
  fromtop = -0.2
---------------------------------------------------------------------------
Script
Description:
Disp&TouchOff
Execute the following script code:
Set WS = Pipettor.Deck.Positions.W1
RightPod.ApproachPosition "W1", WS.LabwareX+WS.WasteX, WS.LabwareY, 6.3

TipLength = World.TipClasses(RightPod.Tip1.Class.Name).Height

ZDisp = WS.Z1 + TipLength + FromTop
```

```
ArrZDisp  = array(ZDisp,ZDisp,ZDisp,ZDisp,ZDisp,ZDisp,ZDisp,ZDisp)
ArrDDisp  = array(0,0,0,0,0,0,0,0)

ArrZ      = array("z1","z2","z3","z4","z5","z6","z7","z8")
ArrD      = array("d1","d2","d3","d4","d5","d6","d7","d8")

RightPod.AbsoluteMoveAxes ArrZ, ArrZDisp,RightPod.SpeedLimit
RightPod.AbsoluteMoveAxes ArrD, ArrDDisp,30 ' was 10
RightPod.RelativeMoveAxes  "x", -WS.WasteX,10
RightPod.RelativeMoveAxes  "y", -0.3,4
RightPod.RelativeMoveAxes  "y",  0.6,4

For i = 1 to 8
   If IsObject(RightPod.VariantDictionary.Get("Tip" & i)) Then
      Set Tip = RightPod.VariantDictionary.Get("Tip" & i)
      Set Contents = Tip.Contents
      For j = 1 to Contents.Count
         Tip.RemoveContent RightPod
      Next
   End If
Next

RightPod.MoveToMaxZ

Air       = RightPod.SystemTrailingAirgap
ArrAirgap = array(Air,Air,Air,Air,Air,Air,Air,Air)

RightPod.AbsoluteMoveAxes ArrD, ArrAirgap,RightPod.STAGSpeed
```

End Procedure

Define GetNewTips

  pattern = ="1,2,3,4,5,6,7,8"

If

If "Not TipsLoaded":

Then

Span-8 Wash

Wash all probes of Pod2 at W1; do a passive wash by dispensing 0 mL while in the wells and 1 mL to waste. Delay for 300 ms after each dispense.

Span-8 New Tips

Get new Span_8_1000uL_LLS tips for probes specified by "=Pattern" on Pod2.

Script

Description:

Execute the following script code:

```
PG.StallUntilETSIs 0

PC.SetGlobal "TipsLoaded", True


' IMPORTANT

' don't use the SetGlobal step for this as it interprets False as a string.

' Example: In a SetGlobal step set MyGlobal False and test it subsequently

' in an If step with condition MyGlobal = False, the result is False i.e. MyGlobal was not
```

considered False.

Set "TipsLoaded" to "True"

End

Else

End

End Procedure

Define DropTips

Span-8 Discard Tips

Discard tips from all probes on Pod2.

Script

Description:

Execute the following script code:

PG.StallUntilETSIs 0

PC.SetGlobal "TipsLoaded", False

Set "TipsLoaded" to "False"

End Procedure

Define StartTimer

index = =""

```
  timername = "Default"

  waittime = "00:00:00"

---------------------------------------------------------------------------

Script

Description:

Execute the following script code:

If IsRunning Then

   PG.StallUntilETSIs 0

   If 1>TLS.SecondsLeft(TimerName & index) Then TLS.SetTimer TimerName & index, WaitTime

End If



---------------------------------------------------------------------------

End Procedure

---------------------------------------------------------------------------

Define WaitTimer

  index = =""

  settimer = True

  timername = =""

  waittime = ="00:00:00"

---------------------------------------------------------------------------

Script

Description:

Execute the following script code:

If IsRunning Then PG.StallUntilETSIs 0

If Not SIM And TimerName <> "" Then _

   Pipettor.LightCurtain.Pause
```

```
---------------------------------------------------------------------------

Script

Description:

Execute the following script code:

'Wait Timer

If IsRunning And TimerName <> "" Then

   If SetTimer Then TLS.SetTimer TimerName & Index, WaitTime

   if 0<TLS.SecondsLeft(TimerName & Index) Then TLS.WaitTimer TimerName & Index

End If


'Resume LightCurtain

If Not SIM Then Pipettor.LightCurtain.Resume


---------------------------------------------------------------------------

End Procedure

---------------------------------------------------------------------------

Define ParkInWashStation

  fromtop = -0.2

  rule = 3

  withtipwash = False

---------------------------------------------------------------------------

If

If "WithTipWash":

---------------------------------------------------------------------------

Then

---------------------------------------------------------------------------

Configure

Bitmap: C:\Program Files\Biomek Software\Bitmaps\Vials.bmp

Description:
```

Caption: Configure

Code: ' Make the wellmap string. Depends on labware -

```
' if number of rows = 8 then tips 1-2-3-4
' can only use the upper 4 rows.
' Condition not implemented - always assumes 8 rows for now.

' First get the labware properties:
rows = Positions(Pipettor.Deck.FindLabwarePosition("WashTips")).Labware.Class.WellsY
cols = Positions(Pipettor.Deck.FindLabwarePosition("WashTips")).Labware.Class.WellsX

Select Case Rule
   Case 2
      Extend "UseTips", "5,6,7,8"
      PC.SetGlobal "TipWashColLower", TipWashColLower + 1
      WA = Split(MFX.TB1.WellsInCol(TipWashColLower, rows*1, cols*1), ",")
      Extend "WellString", WA(4) & "," & WA(5) & "," & WA(6) & "," & WA(7)
   Case Else ' 1 or 3
      Extend "UseTips", "1,2,3,4"
      PC.SetGlobal "TipWashColUpper", TipWashColUpper + 1
      WA = Split(MFX.TB1.WellsInCol(TipWashColUpper, rows*1, cols*1), ",")
      Extend "WellString", WA(0) & "," & WA(1) & "," & WA(2) & "," & WA(3)
End Select
```

Let: [IDispatch]

Weak: [IDispatch]

Prompt: [IDispatch]

Tooltip: Scripted Let

Transfer

Using Pod2, execute the following transfer:

From: WashTips, sections specified by "=WellString", BeadsplusCells

Proceed down first, then left to right.

Start from the beginning of the selection.

Do not set the mark.

Use the following custom technique:

  Use the following pipetting template: OS Span-8 P1000 MixOnly

  Calibration Offset: 0

  Calibration Slope: 1

  Minimum Pipetting Height: 1.5 mm

  Prewet: False

  Aspirate Blowout: True

  Follow Liquid: True

  Height: 0 mm from the top

  Mix: True

  Mix Aspirate Speed: 100µL/s

  Mix Aspirate Height: -10 mm from the top

  Mix Dispense Speed: 100µL/s

  Mix Dispense Height: -10 mm from the top

  Mix Count: 1

  Mix Volume: =MaxVolInTips µL

  Operation speed: 5µL/s

  Tip Touch: True

  Trailing Air Gap: False

  Override Liquid Type Settings

    Aspirate Delay: 200 ms

    Aspirate Speed: 50 µL/s

Blowout Delay: 0 ms

Blowout Volume: 20 µL

Dispense Delay: 0 ms

Dispense Speed: 950 µL/s

Prewet Delay: 0 ms

Prewet Overage: 0 µL

Tip Touch Delay: 0 ms

Tip Touch Speed: 50 µL/s

Trailing Air Gap Volume: 1 µL

Override the technique height by moving to -10 mm from the top.

To: WashTips, 0µL, sections specified by "=WellString", BeadsplusCells

Proceed down first, then left to right.

Start from the beginning of the selection.

Do not set the mark.

Use the following custom technique:

Use the following pipetting template: Span-8 P1000

Calibration Offset: 2.54

Calibration Slope: 1.033

Minimum Pipetting Height: 0.5 mm

Prewet: False

Blowout: False

Follow Liquid: False

Height: 0 mm from the liquid

Mix: False

Mix Aspirate Speed: 100µL/s

Mix Aspirate Height: 0 mm from the liquid

Mix Dispense Speed: 100µL/s

Mix Dispense Height: 0 mm from the liquid

Mix Count: 1

Mix Volume: 900 µL

Operation speed: 70µL/s

Tip Touch: False

Override Liquid Type Settings

Aspirate Delay: 10 ms

Aspirate Speed: 300 µL/s

Blowout Delay: 10 ms

Blowout Volume: 20 µL

Dispense Delay: 20 ms

Dispense Speed: 200 µL/s

Prewet Delay: 0 ms

Prewet Overage: 0 µL

Tip Touch Delay: 300 ms

Tip Touch Speed: 70 µL/s

Trailing Air Gap Volume: 10 µL

Override the technique height by moving to -10 mm from the top.

Dispense up to 1 time(s) per draw.

Create 1 replicate(s) of each source well.

Keep the current tips

Keep the tips when finished.

Stop when finished with Destinations.

Probes specified by "=UseTips" will be used.

End Scripted Let

End

Else

End

Script

Description:

Disp&TouchOff

Execute the following script code:

```
Rightpod.MoveZ  Rightpod.Max.Z-0.5, False

Rightpod.AbsoluteMoveAxes "s", 6.3 ' close all the way


Set WS = Pipettor.Deck.Positions.W1

RightPod.ApproachPosition "W1", WS.LabwareX+WS.WasteX, WS.LabwareY


' At this point there may be tips on 1-4 or 1-8, but never just 5-8.

' Because of that checking Tip1 only is ok.


If IsObject(RightPod.Tip1) Then

   TipLength = World.TipClasses(RightPod.Tip1.Class.Name).Height


   zdsp         = WS.Z1 + TipLength + FromTop


   ArrZ      = array("z1","z2","z3","z4","z5","z6","z7","z8")

   ArrZDisp  = array(zdsp,zdsp,zdsp,zdsp,zdsp,zdsp,zdsp,zdsp)

   ArrD      = array("d1","d2","d3","d4","d5","d6","d7","d8")

   ArrDDisp  = array(0,0,0,0,0,0,0,0)
```

```
   RightPod.AbsoluteMoveAxes ArrZ, ArrZDisp,RightPod.SpeedLimit

   RightPod.AbsoluteMoveAxes ArrD, ArrDDisp,30 ' was 10

   RightPod.RelativeMoveAxes  "x", -WS.WasteX,10

   RightPod.RelativeMoveAxes  "y", -0.3,4

   RightPod.RelativeMoveAxes  "y",  0.6,4


   For i = 1 to 8

      If IsObject(RightPod.VariantDictionary.Get("Tip" & i)) Then

         Set Tip = RightPod.VariantDictionary.Get("Tip" & i)

         Set Contents = Tip.Contents

         For j = 1 to Contents.Count

            Tip.RemoveContent RightPod

         Next

      End If

   Next


   ArrD      = array("d1","d2","d3","d4","d5","d6","d7","d8")

   Air       = RightPod.SystemTrailingAirgap

   ArrAirgap = array(Air,Air,Air,Air,Air,Air,Air,Air)


   RightPod.AbsoluteMoveAxes ArrD, ArrAirgap,RightPod.STAGSpeed

End If

---------------------------------------------------------------------------

End Procedure

---------------------------------------------------------------------------

Define CleanWashStation

  dspeed = 200

  fromtop = 0.6

  yoffset = 0.35
```

Scripted Let

Description:

Code: ' Make sure no tips are loaded

```
Extend "HasTips", IsObject(RightPod.Tip1)  OR _
                  IsObject(RightPod.Tip2)  OR _
                  IsObject(RightPod.Tip3)  OR _
                  IsObject(RightPod.Tip4)  OR _
                  IsObject(RightPod.Tip5)  OR _
                  IsObject(RightPod.Tip6)  OR _
                  IsObject(RightPod.Tip7)  OR _
                  IsObject(RightPod.Tip8)
```

Prompt: [IDispatch]

If

If "HasTips":

Then

Script

Description:

Disp&TouchOff

Execute the following script code:

```
If IsRunning Then TLS.Msg "Cannot clean wash station while tips are loaded"
```

End

Else

Script

Description:

Disp&TouchOff

Execute the following script code:

```
   Rightpod.MoveZ  Rightpod.Max.Z-0.5, False
   Rightpod.AbsoluteMoveAxes "s", 6.3 ' close all the way

   Set WS = Pipettor.Deck.Positions.W1
   RightPod.ApproachPosition "W1", WS.LabwareX+WS.WasteX, WS.LabwareY

   ZDisp     = WS.Z1 + FromTop

   ArrZ      = array("z1","z2","z3","z4","z5","z6","z7","z8")
   ArrZDisp  = array(ZDisp,ZDisp,ZDisp,ZDisp,ZDisp,ZDisp,ZDisp,ZDisp)
   ArrD      = array("d1","d2","d3","d4","d5","d6","d7","d8")
   ArrDDisp  = array(0,0,0,0,0,0,0,0)

   ' empty all tips
   RightPod.ValvesToOutput 255
   RightPod.AbsoluteMoveAxes ArrZ, ArrZDisp,RightPod.SpeedLimit
   RightPod.AbsoluteMoveAxes ArrD, ArrDDisp,30 ' was 10

   ' move to flush position
   ArrZDisp  = array(ZDisp,ZDisp,ZDisp,ZDisp,ZDisp,ZDisp,ZDisp,ZDisp)
   RightPod.AbsoluteMoveAxes ArrZ, ArrZDisp,RightPod.SpeedLimit
   RightPod.RelativeMoveAxes  "x", -WS.WasteX,10
```

Loop

Loop from "1" to "2", incrementing by "1".

Script

Description:

Disp&TouchOff

Execute the following script code:

```
   RightPod.RelativeMoveAxes  "y", -YOffset,4
```

Script

Description:

Disp&TouchOff

Execute the following script code:

```
RightPod.ValvesToInput 255


ArrD      = array("d1","d2","d3","d4","d5","d6","d7","d8")

ArrDDisp  = array(900,900,900,900,900,900,900,900)

RightPod.AbsoluteMoveAxes ArrD, ArrDDisp,DSpeed ' was 10
```

Script

Description:

Disp&TouchOff

Execute the following script code:

```
RightPod.ValvesToOutput 255


ArrD      = array("d1","d2","d3","d4","d5","d6","d7","d8")

ArrDDisp  = array(0,0,0,0,0,0,0,0)
```

```
RightPod.AbsoluteMoveAxes ArrD, ArrDDisp,DSpeed ' was 10
```

Script

Description:

Disp&TouchOff

Execute the following script code:

```
RightPod.RelativeMoveAxes  "y", 2*YOffset,4
```

Script

Description:

Disp&TouchOff

Execute the following script code:

```
RightPod.ValvesToInput 255


ArrD      = array("d1","d2","d3","d4","d5","d6","d7","d8")

ArrDDisp  = array(900,900,900,900,900,900,900,900)

RightPod.AbsoluteMoveAxes ArrD, ArrDDisp,DSpeed ' was 10
```

Script

Description:

Disp&TouchOff

Execute the following script code:

```
RightPod.ValvesToOutput 255


ArrD      = array("d1","d2","d3","d4","d5","d6","d7","d8")

ArrDDisp  = array(0,0,0,0,0,0,0,0)
```

```
RightPod.AbsoluteMoveAxes ArrD, ArrDDisp,DSpeed ' was 10
```

Script

Description:

Disp&TouchOff

Execute the following script code:

```
   RightPod.RelativeMoveAxes  "y", -YOffset,4
```

End Loop

Script

Description:

Disp&TouchOff

Execute the following script code:

```
   ArrD      = array("d1","d2","d3","d4","d5","d6","d7","d8")

   Air       = RightPod.SystemTrailingAirgap

   ArrAirgap = array(Air,Air,Air,Air,Air,Air,Air,Air)


   RightPod.AbsoluteMoveAxes ArrD, ArrAirgap,RightPod.STAGSpeed
```

End

End Scripted Let

End Procedure

```
--------------------------------------------------------------------------

Define CombineColumns

  transfervolume = 100

--------------------------------------------------------------------------

Run getnewtips

  pattern = =OneTip()(MFX.NumSamples)

--------------------------------------------------------------------------

Loop

Loop from "transfer" = "1" to "3", incrementing by "1".

--------------------------------------------------------------------------

Configure

Bitmap: C:\Program Files\Biomek Software\Bitmaps\Vials.bmp

Description:

Caption: Configure

Code: n=MFX.NumSamples

Select Case transfer

   Case 1: scol = Col()(1)(n) & "," & Col()(2)(n): dcol = Col()(1)(n)

   Case 2: scol = Col()(3)(n) & "," & Col()(4)(n): dcol = Col()(2)(n)

   Case 3: scol = Col()(5)(n) & "," & Col()(6)(n): dcol = Col()(3)(n)

End Select


'determine the asp height

Set LW = Positions(Labware.Location("Cryo1")).Labware

LW.ConfigureAmounts TransferVolume

aspheightcm = LW.HeightFromVolume(TransferVolume, 1)

              ' this is the height in CM

              ' needs to be multiplied by 10 because the step expects MM


Extend "SrcCol",   scol
```

Extend "DestCol", dcol

Extend "ZAsp",   (aspheightcm * 10) - 3 ' convert cm to mm; 3 mm below liquid

Prompt: [IDispatch]

Tooltip: Scripted Let

Transfer

Using Pod2, execute the following transfer:

From: Cryo1, sections specified by "=SrcCol", BeadsplusCells

Proceed down first, then left to right.

Start from the beginning of the selection.

Do not set the mark.

Use the following custom technique:

  Use the following pipetting template: OS Span-8 P1000 SlowRetract

  Calibration Offset: 2.54

  Calibration Slope: 1.033

  Minimum Pipetting Height: 0 mm

  Prewet: False

  Aspirate Blowout: True

  Follow Liquid: True

  Height: 0 mm from the liquid

  Mix: False

  Mix Aspirate Speed: =100µL/s

  Mix Aspirate Height: =ZAsp mm from the liquid

  Mix Dispense Speed: 100µL/s

  Mix Dispense Height: 2 mm from the liquid

  Mix Count: 1

  Mix Volume: 0 µL

Operation speed: 70µL/s

Tip Touch: False

Trailing Air Gap: False

Override Liquid Type Settings

Aspirate Delay: 10 ms

Aspirate Speed: 80 µL/s

Blowout Delay: 1000 ms

Blowout Volume: 20 µL

Dispense Delay: 20 ms

Dispense Speed: 200 µL/s

Prewet Delay: 200 ms

Prewet Overage: 0 µL

Tip Touch Delay: 300 ms

Tip Touch Speed: 70 µL/s

Trailing Air Gap Volume: 10 µL

Override the technique height by moving to =ZAsp mm from the bottom.

To: Cryo2, =TransferVolumeµL, sections specified by "=DestCol", BeadsplusCells

Proceed down first, then left to right.

Start from the beginning of the selection.

Do not set the mark.

Use the following custom technique:

Use the following pipetting template: OS Span-8 P1000 Blowout at LiqLevel 2

Calibration Offset: 2.54

Calibration Slope: 1.033

Minimum Pipetting Height: 0.5 mm

Prewet: False

Blowout: False

Follow Liquid: True

Height: 0 mm from the liquid

Mix: False

Mix Aspirate Speed: 100µL/s

Mix Aspirate Height: 0 mm from the liquid

Mix Dispense Speed: 100µL/s

Mix Dispense Height: 0 mm from the liquid

Mix Count: 1

Mix Volume: 900 µL

Operation speed: 70µL/s

Tip Touch: False

Override Liquid Type Settings

Aspirate Delay: 10 ms

Aspirate Speed: 300 µL/s

Blowout Delay: 10 ms

Blowout Volume: 20 µL

Dispense Delay: 20 ms

Dispense Speed: 200 µL/s

Prewet Delay: 0 ms

Prewet Overage: 0 µL

Tip Touch Delay: 300 ms

Tip Touch Speed: 70 µL/s

Trailing Air Gap Volume: 10 µL

Override the technique height by moving to 2 mm from the bottom.

Dispense up to 1 time(s) per draw.

Create 1 replicate(s) of each source well.

Keep the current tips

Keep the tips when finished.

Stop when finished with Sources.

Probes specified by "=OneTip()(MFX.NumSamples)" will be used.

End Scripted Let

End Loop

Run droptips

End Procedure

Define SetStepDone

  wait = False

Script

Description:

Execute the following script code:

```
If Wait Then PG.StallUntilETSIs 0
MFX.SetStepDone MFX.NextStep, True
```

End Procedure

End Group

Group:

Startup Group

Script

Description:

Initialize

Execute the following script code:

```
PC.SetGlobal "KFile", MFX.CMN.RuntimeFolder & "\CD8Prep.txt"

If Not KW.ExistFile(KFile) Then KW.CreateNewFile KFile

MFX.PrepareDialog KFile, "G:\Stepnames2.txt",IsRunning

SetStepNames

MFX.WorkOffline = WorkOffline

InitTimers


Sub SetStepNames

   'MFX.SetStepName 1, "  1: Downstack F-Plate"

   'MFX.SetStepName 2, "  2: Move F-Plate to DSP"

   ' etc.

End Sub


Sub InitTimers

   TLS.SetTimer "Shaking/Incubating", "00:00:00"

   TLS.SetTimer "Settling...",        "00:00:00"

   TLS.SetTimer "Incubating...",      "00:00:00"

   'TLS.SetTimer "xx", "00:00:00"

   'TLS.SetTimer "xx", "00:00:00"

   'TLS.SetTimer "xx", "00:00:00"

End Sub
```

If

If "Not IsNull (LeftPod.GrippedLabware)":

Then

Script

Description:

Execute the following script code:

```
PG.StallUntilETSIs 0

If IsRunning AND NOT SIM Then Pipettor.LightCurtain.Pause
```

Script

Description:

Execute the following script code:

```
PG.StallUntilETSIs 0

If IsRunning Then

   TLS.Msg "The gripper seems to be holding something. Carefully remove it from the
gripper and put it back where it came from. Then click ok."

   If NOT SIM Then Pipettor.LightCurtain.Resume

End If
```

Script

Description:

Execute the following script code:

```
PG.StallUntilETSIs 0

LeftPod.Gripper.Retract
```

End

Else

Script

Description:

Execute the following script code:

PG.StallUntilETSIs 0

LeftPod.Gripper.Retract

End

Instrument Setup

Deck: FX1_RotatingALP

Items:

Cyto1: Nothing

Cyto2: Nothing

Orbital1: Nothing

P10: BD_50ml_1 named Blood with an unknown amount of an unknown liquid.

P11: Nothing

P2: db_4Qtr_45ml named Parked with an unknown amount of an unknown liquid.

P3: rm4_tubeholder named Cryo2 with an unknown amount of an unknown liquid.tipboxlid_lowgrip with an unknown amount of an unknown liquid.

P4: PlateRotator with an unknown amount of an unknown liquid.IM_6W_Portrait named Buffer with an unknown amount of an unknown liquid.

P5: Span_8_1000uL_LLS named Parked. Discard the tips to "<Tipbox>". When done, move the box to "<Home>". Use these tips "1" times.

P6: Span_8_200uL named TB1. Discard the tips to "<Tipbox>". When done, move the box to

"<Home>". Use these tips "1" times. tipboxlid_lowgrip with an unknown amount of an unknown liquid.

P7: Nothing

P8: Span_8_1000uL_LLS. Use these tips 1 time.

P9: Span_8_1000uL_LLS named TB2. Discard the tips to "<Tipbox>". When done, move the box to "<Home>". Use these tips "1" times.

Pelt1: RM4_Magnet named Magnet with an unknown amount of an unknown liquid.

R1: rm4_tubeholder named Cryo1 with an unknown amount of an unknown liquid.tipboxlid_lowgrip with an unknown amount of an unknown liquid.

TL1: AP96_20uL named StoreLid. Discard the tips to "<Tipbox>". When done, move the box to "<Home>". Use these tips "1" times.

TR1: Nothing

TR2: Nothing

TS1: Nothing

W1: Nothing

Script

Description:

Execute the following script code:

```
PG.StallUntilETSIs 0
If IsRunning AND NOT SIM Then Pipettor.LightCurtain.Pause
```

Script

Description:

Execute the following script code:

```
'MFX.DefineDefault ParameterName, Value


If IsRunning Then
   MFX.WorkOffline = WorkOffline
```

```
MFX.ShowDialog

If "" = MFX.P4ShakeTime Then

   If NOT SIM Then Pipettor.LightCurtain.Resume

   World.Globals.ErrorGenerator.AbortRun 0, LeftPod, True

Else

   Select Case MFX.NextStep

      Case 1,2,3:

         PosCryo1="R1"

         PosCryo2="P3"

         Cryo1HasLid = True

         Cryo2HasLid = True

      Case 4,5,6,10,11,12,13,17,18:

         PosCryo1="Orbital1"

         PosCryo2="P3"

         Cryo1HasLid = True

         Cryo2HasLid = True

      Case 7,8,9,14,15,16:

         PosCryo1="Pelt1"

         PosCryo2="P3"

         Cryo1HasLid = True

         Cryo2HasLid = True

      Case 19,20,21:

         PosCryo1="Orbital1"

         PosCryo2="P3"

         Cryo1HasLid = True

         Cryo2HasLid = True

      Case 22, 23:

         PosCryo1="Pelt1"
```

```
    PosCryo2="P3"
    Cryo1HasLid = True
    Cryo2HasLid = False
Case 24,25,29:
    PosCryo1="Pelt1"
    PosCryo2="P3"
    Cryo1HasLid = False
    Cryo2HasLid = False
Case 26,27,28,30,31:
    PosCryo1="Orbital1"
    PosCryo2="P3"
    Cryo1HasLid = False
    Cryo2HasLid = False
Case 32:
    PosCryo1="P6"
    PosCryo2="P3"
    Cryo1HasLid = False
    Cryo2HasLid = False
Case 33,34,35,39,40:
    PosCryo1="P6"
    PosCryo2="Pelt1"
    Cryo1HasLid = False
    Cryo2HasLid = False
Case 36,37,38:
    PosCryo1="P6"
    PosCryo2="Orbital1"
    Cryo1HasLid = True
    Cryo2HasLid = True
```

```
    Case Else ' something's fishy - abort
        TLS.Msg "Illegal state - NextStep = " & MFX.NextStep & vblf & "Method will
abort."
        World.Globals.ErrorGenerator.AbortRun 0, LeftPod, True
End Select

' TB1 and Cryo1: Remove tipbox lid so that all volume
' manipulations are applied to the correct labware
' but store the lid type

POS = Pipettor.Deck.FindLabwarePosition("TB1")
Set LW = Positions(POS).Labware
Lid_TB1 = LW.Class.Name
Positions(POS).RemoveLabware ' lid
Pipettor.Deck.LabwareRemovedFromPosition LW, POS

POS = Pipettor.Deck.FindLabwarePosition("Cryo1")
Set LW = Positions(POS).Labware
Lid_Cryo1 = LW.Class.Name
Positions(POS).RemoveLabware ' lid
Pipettor.Deck.LabwareRemovedFromPosition LW, POS

POS = Pipettor.Deck.FindLabwarePosition("Cryo2")
Set LW = Positions(POS).Labware
Lid_Cryo2 = LW.Class.Name
Positions(POS).RemoveLabware ' lid
Pipettor.Deck.LabwareRemovedFromPosition LW, POS
```

```
Set LW = Positions(Labware.Location("TB1")).Labware ' 200 ul tips
For n = 1 to 96
   LW.ConfigureWellAmount n, MFX.TB1.HasTip(n)
Next

Set LW = Positions(Labware.Location("TB2")).Labware ' 1 ml tips
For n = 1 to 96
   LW.ConfigureWellAmount n, MFX.TB2.HasTip(n)
Next

' Now: Put the vials in the correct positions.
' C1 and C2 are the CURRENT positions
C1 = Pipettor.Deck.FindLabwarePosition("Cryo1") ' start position
C2 = Pipettor.Deck.FindLabwarePosition("Cryo2") ' start position

' record the vial rack types
LW_C1=Positions(C1).Labware.Class.Name
LW_C2=Positions(C2).Labware.Class.Name

' replace Cryo1 and put the new one in position PosCryo1
Set LW = Positions(C1).Labware
LWName = LW.Properties.Name
Positions(C1).RemoveLabware
Pipettor.Deck.LabwareRemovedFromPosition LW, C1
Positions(PosCryo1).AddNewLabware LW_C1, World
Pipettor.Deck.LabwareAddedToPosition LW, PosCryo1
Set LW = Positions(PosCryo1).Labware
LW.Properties.Name = LWName
```

```
' and Cryo2
Set LW = Positions(C2).Labware
LWName = LW.Properties.Name
Positions(C2).RemoveLabware
Pipettor.Deck.LabwareRemovedFromPosition LW, C2
Positions(PosCryo2).AddNewLabware LW_C2, World
Pipettor.Deck.LabwareAddedToPosition LW, PosCryo2
Set LW = Positions(PosCryo2).Labware
LW.Properties.Name = LWName

' put tipbox lids back on TB1, Cryo1, and Cryo2 (based on status from above)
POS = Pipettor.Deck.FindLabwarePosition("TB1")
Set LW = Positions(POS).Labware
Positions(POS).AddNewLabware Lid_TB1, World
Pipettor.Deck.LabwareAddedToPosition LW, POS

If Cryo1HasLid Then
   Set LW = Positions(PosCryo1).Labware
   Positions(PosCryo1).AddNewLabware Lid_Cryo1, World
   Pipettor.Deck.LabwareAddedToPosition LW, PosCryo1
End If

If Cryo2HasLid Then
   Set LW = Positions(PosCryo2).Labware
   Positions(PosCryo2).AddNewLabware Lid_Cryo2, World
   Pipettor.Deck.LabwareAddedToPosition LW, PosCryo2
End If
```

```
      If NOT SIM Then Pipettor.LightCurtain.Resume
   End If ' ShakeTime = ""
End If

'PC.SetGlobal "TipWashColUpper", 0
'PC.SetGlobal "TipWashColLower", 0

'If MFX.S3bDone Then
'   MoveTopLW Pipettor.Deck.FindLabwarePosition("Cryo2"), "TR2"
   'In this case the lid is not automatically removed from TR2
'   Positions.TR2.RemoveAllLabware
'End If

Sub MoveTopLW(From, PosTo)
      Set LW = Positions(From).Labware
      ' record the labwareclass
      LWClass = LW.Class.Name
      Positions(From).RemoveLabware
      Pipettor.Deck.LabwareRemovedFromPosition LW, From

      Positions(PosTo).AddNewLabware LWClass, World
      Pipettor.Deck.LabwareAddedToPosition LW, PosTo
End Sub
```

If

If "MFX.UsePeltier":

Then

Peltier1: Initialize

Incubate Pelt1 at =MFX.TempPeltierC for 00:00:01

Position: Pelt1

Command: Incubate

Module Name: Peltier1

Set Temperature?: -1

Temperature: =MFX.TempPeltier

Total Time: 00:00:01

TShake1: Initialize

Incubate TS1 at =MFX.TempPeltierC for 00:00:01

Position: TS1

_Commented: -1

Total Time: 00:00:01

Continue Shaking: -1

Deluxe Shake: 0

Shake RPM: 700

Shake Style: Diagonal (NE to SW)

Shake Style1: Horizontal

Shake Style2: Vertical

Shake Time1: 5

Shake Time2: 5

Temp Set Point: =MFX.TempPeltier

Use Shake: 0

Use Temp: -1

End

Else

End

Instrument Setup

Deck: FX1_RotatingALP

Pause to confirm layout.

Items:

Cyto1: As Is.

Cyto2: As Is.

Orbital1: As Is.

P10: As Is.

P11: As Is.

P2: As Is.

P3: As Is.

P4: As Is.

P5: As Is.

P6: As Is.

P7: As Is.

P8: As Is.

P9: As Is.

Pelt1: As Is.

R1: As Is.

TL1: As Is.

TR1: As Is.

TR2: As Is.

TS1: As Is.

W1: As Is.

Group:

Purge System

Run droptips

Script

Description:

Disp&TouchOff

Execute the following script code:

```
   Rightpod.MoveZ  Rightpod.Max.Z-0.5, False

   Rightpod.AbsoluteMoveAxes "s", 6.3 ' close all the way


   Set WS = Pipettor.Deck.Positions.W1

   RightPod.ApproachPosition "W1", WS.LabwareX+WS.WasteX, WS.LabwareY


   ZDisp     = WS.Z1 + 1 ' 1 cm above WS


   ArrZ      = array("z1","z2","z3","z4","z5","z6","z7","z8")

   ArrZDisp  = array(ZDisp,ZDisp,ZDisp,ZDisp,ZDisp,ZDisp,ZDisp,ZDisp)

   ArrD      = array("d1","d2","d3","d4","d5","d6","d7","d8")

   ArrDDisp  = array(0,0,0,0,0,0,0,0)


   ' empty all tips

   RightPod.ValvesToOutput 255

   RightPod.AbsoluteMoveAxes ArrZ, ArrZDisp,RightPod.SpeedLimit

   RightPod.AbsoluteMoveAxes ArrD, ArrDDisp,30 ' was 10
```

```
   ' move to flush position
   'ArrZDisp  = array(ZDisp,ZDisp,ZDisp,ZDisp,ZDisp,ZDisp,ZDisp,ZDisp)
   'RightPod.AbsoluteMoveAxes ArrZ, ArrZDisp,RightPod.SpeedLimit
   'RightPod.RelativeMoveAxes  "x", -WS.WasteX,10
```

Loop

Loop from "1" to "4", incrementing by "1".

Script

Description:

Disp&TouchOff

Execute the following script code:

```
RightPod.ValvesToInput 255

ArrD      = array("d1","d2","d3","d4","d5","d6","d7","d8")
ArrDDisp  = array(900,900,900,900,900,900,900,900)
RightPod.AbsoluteMoveAxes ArrD, ArrDDisp,100'DSpeed ' was 10
```

Script

Description:

Disp&TouchOff

Execute the following script code:

```
RightPod.ValvesToOutput 255

ArrD      = array("d1","d2","d3","d4","d5","d6","d7","d8")
ArrDDisp  = array(0,0,0,0,0,0,0,0)
RightPod.AbsoluteMoveAxes ArrD, ArrDDisp,100'DSpeed ' was 10
```

End Loop

Loop

Loop from "1" to "10", incrementing by "1".

Script

Description:

Execute the following script code:

```
PG.StallUntilETSIs 0

PC.SetGlobal "DonePurging", False

If IsRunning Then

   ' 7 = vbNo, 4 = vbYesNo

   If 7 = TLS.MsgBx("Click" & vblf & _

                "- Yes to continue purging" & vblf & _

                "- No to stop purging", 4, "Continue Purging?") Then _

          PC.SetGlobal "DonePurging", True

End If 'IsRunning

If Not SIM Then Pipettor.LightCurtain.Resume
```

If

If "DonePurging = True":

Then

Script

Description:

Disp&TouchOff

Execute the following script code:

```
   ArrD      = array("d1","d2","d3","d4","d5","d6","d7","d8")

   Air       = RightPod.SystemTrailingAirgap

   ArrAirgap = array(Air,Air,Air,Air,Air,Air,Air,Air)


   RightPod.AbsoluteMoveAxes ArrD, ArrAirgap,RightPod.STAGSpeed
```

Break out of 1 loop(s).

End

Else

Loop

Loop from "1" to "4", incrementing by "1".

Script

Description:

Disp&TouchOff

Execute the following script code:

```
RightPod.ValvesToInput 255


ArrD      = array("d1","d2","d3","d4","d5","d6","d7","d8")

ArrDDisp  = array(900,900,900,900,900,900,900,900)

RightPod.AbsoluteMoveAxes ArrD, ArrDDisp,100'DSpeed ' was 10
```

Script

Description:

Disp&TouchOff

Execute the following script code:

```
RightPod.ValvesToOutput 255


ArrD      = array("d1","d2","d3","d4","d5","d6","d7","d8")

ArrDDisp  = array(0,0,0,0,0,0,0,0)

RightPod.AbsoluteMoveAxes ArrD, ArrDDisp,100'DSpeed ' was 10
```

End Loop

End

End Loop

End Group

Group:

Init Tips

Run droptips

Span-8 Wash

Wash all probes of Pod2 at W1; do a passive wash by dispensing 0 mL while in the wells and 6 mL to waste. Delay for 300 ms after each dispense.

Wash only the probes that have been used.

End Group

If

If "Labware.Location("Cryo1") = "Orbital1"":

Then

Device Action

Send the following command to "OrbitalShakerALP0": "Clamp".

Parameters:

End

Else

End

End Group

Group:

Steps

Group

If

If "MFX.NextStep = 1":

Then

Script

Description:

Labware Pickup from Cryo1

Execute the following script code:

```
Option Explicit

' This script duplicates the functionality of the first half of the Move Labware step.

' Modified 3/7/2003 by MAG to lift the labware to a specific height off the deck

' (height measured at top of the labware) which was useful for a specific customer

' reading barcodes via their own reader, with labels always a certain distance from

' the top of the labware.


Set e = CreateObject("World.EngineObject")


Dim Source, Target, PodName

Dim Pod, CurDeck, OrigPodSpeed, LWSpeedLimit


' PodName        The name of the pod to use

PodName = StepDictionary.Let.PodName

if IsEmpty(PodName) or PodName = "" then err.raise 1050, , "PodName must be defined"

Set Pod = World.Devices.Pipettor1(PodName)

OrigPodSpeed = Pod.CurrentSpeed


Set CurDeck = World.Devices.Pipettor1.Deck


' Source         Name of the source position (string)

' Need to use Deck.FindLabwarePosition,
```

```
' in case labware is referred to by name instead of location
Source = CurDeck.FindLabwarePosition(e.EvaluateExpression(StepDictionary.Let.Source))
'tls.msg MFX.NextStep & " " & source
if IsEmpty(Source) or Source = "" then err.raise 1050, , "Source must be defined"

' Get the depth to grab a piece of labware at.
Dim Depth
Depth = e.EvaluateExpression(StepDictionary.Let.Depth)
if IsEmpty(Depth)         then err.raise 1050, , "Depth must be defined"
If Depth < 1              then err.raise 1050, , "Depth must be at least 1"

' Convert Source and Target names into objects
Dim Src
Set Src = Pipettor.Deck.Positions(Source)

' Get the Source Labware
Dim srcLabware
World.Volatile.Depth = Depth
Set srcLabware = Src.GetLabwareAtDepth(Depth)

' Get the source labware height  [DON'T NEED THIS ANYMORE]
' Dim srcLwHeight
' srcLwHeight = srcLabware.Class.Height

Dim Z
Z = CDbl(Src.Z(Pod))

' Get offsets from position to the labware at source position
Dim sx, sy, sz
```

```
sx = CDbl(Src.GetOneLabwareOffsetAtDepth(Depth, "X"))
sy = CDbl(Src.GetOneLabwareOffsetAtDepth(Depth, "Y"))
sz = CDbl(Src.GetOneLabwareOffsetAtDepth(Depth, "Z"))

pd = CDbl(Pod.VariantDictionary.GetDefault("GripperDOffset", 0.0))

' Get the labware gripper offsets and squeeze values
Dim gx, gy, gz, pd
Dim Squeeze, Unsqueeze
Select Case Pod.PodType
   Case "ATTILA"
      gx = CDbl(srcLabware.Class.GripperInfo.MultiChannel.GripperXOffset)
      gy = CDbl(srcLabware.Class.GripperInfo.MultiChannel.GripperYOffset)
      gz = CDbl(srcLabware.Class.GripperInfo.MultiChannel.GripperZOffset)
      Squeeze = CDbl(Pod.DAxisFromGripper(CDbl(pd) +
CDbl(srcLabware.Class.GripperInfo.MultiChannel.Squeeze)))
      Unsqueeze = CDbl(Pod.DAxisFromGripper(CDbl(pd) +
CDbl(srcLabware.Class.GripperInfo.MultiChannel.Unsqueeze)))
'tls.msg pod.podtype
'tls.msg gx & " " & gy & " " & gz & " " & Squeeze & " " & Unsqueeze
   Case Else ' need to fill in code for NX Gripper
End Select

' Get the Pod gripper offsets
Dim px, py, pz
px = CDbl(Pod.GripperXOffset)
py = CDbl(Pod.GripperYOffset)
pz = CDbl(Pod.GripperZOffset)
```

```
Dim LiftHeight, e

LiftHeight = e.EvaluateExpression(StepDictionary.Let.LiftHeight)

' Temporarily save the height of the move
World.Volatile.LiftHeight = LiftHeight

'Reserve the resources used, i.e., the source position and the Pod
World.Globals.ResourceReserver.ReserveResources Array(Src, Pod)

' Open the source position
Src.Open True, Depth

' Start Moving
Pod.ApproachPosition Source, sx+gx-px, sy+gy-py, Z + sz+gz-pz, False, Unsqueeze

' Extend the grippers
Pod.GripperExtend

' Grab Labware
Pod.AbsMove ,,,Squeeze

' Enforce the labware's speed limit
LWSpeedLimit = srcLabware.Class.SpeedLimit
If LWSpeedLimit < OrigPodSpeed Then Pod.SetSpeed LWSpeedLimit

' Notify software that the source labware is now in the grippers
' Version 2.x syntax: Pod.GetLabware Source, Depth
```

```
Pod.Gripper.GetLabwareObject Source, Depth

' Move to a safe height above the position (original function commented out)
' Pod.MoveToSafe

If LiftHeight < 0 Then err.raise 1050, ,  "Lift Height too low ; would result in downward
motion"

Pod.RelativeMoveAxes "Z", LiftHeight

' Close the source position
' Src.Close True, Depth

'Restore original pod speed now that the move is finished
Pod.SetSpeed OrigPodSpeed
```

RotatingALP

  Position: R1

  Rotate to Orientation: Portrait (90∞ counterclockwise)

Loop

Loop from "RoundsCompleted" = "=MFX.RoundsCompleted(1) " to "=MFX.TotalRounds(1)",
incrementing by "1".

If

If "MFX.RoundsCompleted(1)=MFX.TotalRounds(1)":

Then

Break out of 1 loop(s).

End

Else

End

Swap Labware, Configure Blood Step

Bitmap: C:\Program Files\Biomek Software\Bitmaps\Vials.bmp

Description:

Caption: Swap Labware, Configure Blood Step

```
Code: ALP = Labware.Location("Blood")

Set POS = Positions(ALP)

Set LW = POS.Labware


LWName = LW.Properties.Name

POS.RemoveLabware

Pipettor.Deck.LabwareRemovedFromPosition LW, ALP

POS.AddNewLabware "BD_50ml_" & (1+RoundsCompleted), World

Pipettor.Deck.LabwareAddedToPosition LW, ALP

Set LW = POS.Labware

LW.Properties.Name = LWName


LW.ConfigureAmounts (MFX.P1VolBlood * 6) ' for 6 vials


nTrips = TLS.NumTrips(MFX.P1VolBlood, 950)
```

Extend "NumTrips", nTrips

Extend "VolTrip", MFX.P1VolBlood/nTrips

Prompt: [IDispatch]

Tooltip: Scripted Let

Span-8 Discard Tips

Discard tips from all probes on Pod2.

Span-8 Wash

Wash all probes of Pod2 at the closest wash station; do a passive wash by dispensing 1 mL while in the wells and 1 mL to waste. Delay for 300 ms after each dispense.

Wash only the probes that have been used.

Span-8 New Tips

Get new Span_8_1000uL_LLS tips for probes 1-6 on Pod2.

Loop

Loop from "Trip" = "1" to "=NumTrips", incrementing by "1".

Calculate Asp & Disp Heights

Bitmap: C:\Program Files\Biomek Software\Bitmaps\Vials.bmp

Description:

Caption: Calculate Asp & Disp Heights

Code: ' Asp Height

Set LW = Positions(Labware.Location("Blood")).Labware

VolAfterAsp = 6 * (MFX.P1VolBlood - (Trip * VolTrip))

AspHeightCM = LW.HeightFromVolume(VolAfterAsp, 1)

```
' start aspirating at 3 mm below liqlevel

' then follow down


Extend "ZAsp", (AspHeightCM * 10) - 3


'------------------------------------------------------


' Disp Height

Set LW = Positions(Labware.Location("Cryo1")).Labware

VolBeforeDisp = VolTrip *(Trip-1)

DispHeightCM = LW.HeightFromVolume(VolBeforeDisp, 1)


' start dispensing at 2 mm below liqlevel

' then follow up & blowout at dispheight


Extend "ZDisp", (DispHeightCM * 10) - 2
```

Prompt: [IDispatch]

Tooltip: Scripted Let

Transfer

Using Pod2, execute the following transfer:

From: Blood, with the following pattern:

```
   1
 A ><
```

, Blood_HighVol

Proceed down first, then left to right.

Start from the beginning of the selection.

Set the mark at the last well transferred.

Use the following custom technique:

Use the following pipetting template: OS Span-8 MultiDispense Slow Retract FillVials

Calibration Offset: 0

Calibration Slope: 1.05

Minimum Pipetting Height: 1.5 mm

Prewet: False

Aspirate Blowout: True

Follow Liquid: True

Height: 0 mm from the top

Mix: False

Mix Aspirate Speed: 100µL/s

Mix Aspirate Height: 0 mm from the top

Mix Dispense Speed: 100µL/s

Mix Dispense Height: 0 mm from the top

Mix Count: 1

Mix Volume: 10 µL

Operation speed: 70µL/s

Tip Touch: False

Trailing Air Gap: True

Override Liquid Type Settings

Aspirate Delay: 200 ms

Aspirate Speed: 200 µL/s

Blowout Delay: 0 ms

Blowout Volume: 20 µL

Dispense Delay: 0 ms

Dispense Speed: 200 µL/s

Prewet Delay: 0 ms

Prewet Overage: 0 µL

Tip Touch Delay: 0 ms

Tip Touch Speed: 50 µL/s

Trailing Air Gap Volume: 5 µL

Override the technique height by moving to =ZAsp mm from the bottom.

To: Cryo1, =VolTripµL, sections specified by "=ColWells()(1+RoundsCompleted)", Blood_HighVol

Proceed down first, then left to right.

Start from the beginning of the selection.

Set the mark at the last well transferred.

Use the following custom technique:

Use the following pipetting template: OS Span-8 P1000 TouchOffOnLiquid 2

Calibration Offset: 0

Calibration Slope: 1

Minimum Pipetting Height: 1.5 mm

Prewet: False

Blowout: True

Follow Liquid: True

Height: 0 mm from the top

Mix: False

Mix Aspirate Speed: 100µL/s

Mix Aspirate Height: 0 mm from the liquid

Mix Dispense Speed: 100µL/s

Mix Dispense Height: 0 mm from the liquid

Mix Count: 1

Mix Volume: 10 µL

Operation speed: 70µL/s

Tip Touch: False

Override Liquid Type Settings

Aspirate Delay: 200 ms

Aspirate Speed: 50 µL/s

Blowout Delay: 200 ms

Blowout Volume: 20 µL

Dispense Delay: 5000 ms

Dispense Speed: 200 µL/s

Prewet Delay: 0 ms

Prewet Overage: 0 µL

Tip Touch Delay: 0 ms

Tip Touch Speed: 50 µL/s

Trailing Air Gap Volume: 10 µL

Override the technique height by moving to =ZDisp mm from the bottom.

Dispense up to 1 time(s) per draw.

Create 1 replicate(s) of each source well.

Keep the current tips

Keep the tips when finished.

Stop when finished with Destinations.

Probes 1-6 will be used.

End Scripted Let

End Loop

Span-8 Discard Tips

Discard tips from all probes on Pod2.

```
End Scripted Let

---------------------------------------------------------------------------

Run setstepdone

  wait = true

---------------------------------------------------------------------------

End Loop

---------------------------------------------------------------------------

RotatingALP

  Position: R1

  Rotate to Orientation: Landscape

---------------------------------------------------------------------------

Script

Description:

Labware Place to Cryo1

Execute the following script code:

Option Explicit

' This script duplicates the functionality of the Move Labware step.

' It has been modified to only place the labware after another step has picked it up.

Dim e

Set e = CreateObject("World.EngineObject")

Dim Target, PodName

Dim Pod, CurDeck, OrigPodSpeed, LWSpeedLimit


' PodName        The name of the pod to use

PodName = StepDictionary.Let.PodName

if IsEmpty(PodName) or PodName = "" then err.raise 1050, , "PodName must be defined"

Set Pod = World.Devices.Pipettor1(PodName)

OrigPodSpeed = Pod.CurrentSpeed
```

```
Set CurDeck = World.Devices.Pipettor1.Deck

' Target         Name of the target position (string)
' Need to use Deck.FindLabwarePosition,
' in case labware is referred to by name instead of location
Target = CurDeck.FindLabwarePosition(e.EvaluateExpression(StepDictionary.Let.Target))

if IsEmpty(Target) or Target = "" then err.raise 1050, , "Target must be defined"

If Not IsObject(Pod.GrippedLabware) Then err.raise 1050, , "There is no labware in the
gripper."
Dim glw
set glw = Pod.GrippedLabware(Pod.GrippedLabware.Count - 1) ' The gripped labware is an
array with the bottom piece at the highest index

' Convert Source and Target names into objects
Dim Dst
Set Dst = Pipettor.Deck.Positions(Target)

Dim Z
Dim sx, sy, sz
' Dim gx, gy, gz, pd
'pd = CDbl(Pod.VariantDictionary.GetDefault("GripperDOffset", 0.0))

' Get the squeeze values
'Dim Unsqueeze
'Unsqueeze = CDbl(Pod.DAxisFromGripper(CDbl(pd) + CDbl(glw.Class.Unsqueeze)))
```

```
' Get the Pod gripper offsets
Dim px, py, pz
px = CDbl(Pod.GripperXOffset)
py = CDbl(Pod.GripperYOffset)
pz = CDbl(Pod.GripperZOffset)

' Enforce the labware's speed limit
'LWSpeedLimit = srcLabware.Class.SpeedLimit
'If LWSpeedLimit < OrigPodSpeed Then Pod.SetSpeed LWSpeedLimit

'Get height of destination position
Z = CDbl(Dst.Z(Pod))

' Get offsets from position to the labware at target position
Dim tx, ty, tz, dx, dy, dz
tx = CDbl(Dst.GetOneLabwareOffsetAtDepth(1, "X"))
ty = CDbl(Dst.GetOneLabwareOffsetAtDepth(1, "Y"))
tz = CDbl(Dst.GetOneLabwareOffsetAtDepth(1, "Z"))

' Get the Target Labware
Dim dstLabware
dx = 0
dy = 0
dz = 0
dstLabware = Null
if (Dst.StackDepth > 0) then
  Set dstLabware = Dst.GetLabwareAtDepth(1)
end if
```

```
' Get Destination Stack Offsets (really Per-Labware Offsets)
dx = CDbl(Dst.GetOneStackOffset(glw, dstLabware, "X"))
dy = CDbl(Dst.GetOneStackOffset(glw, dstLabware, "Y"))
dz = CDbl(Dst.GetOneStackOffset(glw, dstLabware, "Z"))

' Get the labware gripper offsets (added-in by MAG 3/7/2003)
World.Volatile.MyDest = Dst

'Dim gx, gy, gz, pd
'gx = CDbl(glw.Class.GripperXOffset)
'gy = CDbl(glw.Class.GripperYOffset)
'gz = CDbl(glw.Class.GripperZOffset)

pd = CDbl(Pod.VariantDictionary.GetDefault("GripperDOffset", 0.0))

' Get the labware gripper offsets and squeeze values
Dim gx, gy, gz, pd
Dim Unsqueeze
Select Case Pod.PodType
   Case "ATTILA"
      gx = CDbl(glw.Class.GripperInfo.MultiChannel.GripperXOffset)
      gy = CDbl(glw.Class.GripperInfo.MultiChannel.GripperYOffset)
      gz = CDbl(glw.Class.GripperInfo.MultiChannel.GripperZOffset)
      Unsqueeze = CDbl(Pod.DAxisFromGripper(CDbl(pd) +
CDbl(glw.Class.GripperInfo.MultiChannel.Unsqueeze)))
'tls.msg pod.podtype
'tls.msg gx & " " & gy & " " & gz & " "  & " " & Unsqueeze
   Case Else ' need to fill in code for NX Gripper
```

```
End Select




' Open Destination
Dst.Open True, 0

' Start Moving
Pod.ApproachPosition Target, CDbl(tx+dx+gx-px), CDbl(ty+dy+gy-py), CDbl(Z + tz+dz+gz-pz),
True

Pod.MoveZ CDbl(Z + tz + dz + gz - pz)

' Put Labware
Pod.AbsMove ,,,Unsqueeze

' Notify software the labware that was in the grippers is now at the target
' old v. 2.x syntax Pod.PutLabware Target
Pod.Gripper.PutLabwareObject Target

' Retract grippers
Pod.GripperRetract

' Close the destination position
Dst.Close True, 0

'Restore original pod speed now that the move is finished
'Pod.SetSpeed OrigPodSpeed
```

' Move to a safe height

Pod.MoveToSafe

End

Else

End

End Group

Group

If

If "MFX.NextStep = 2":

Then

Move Labware

Move the top "1" plates at "Cryo2" to "StoreLid" using pod "Pod1".

Loop

Loop from "RoundsCompleted" = "=MFX.RoundsCompleted(2) " to "=MFX.TotalRounds(2)",

incrementing by "1".

If

If "MFX.RoundsCompleted(2)=MFX.TotalRounds(2)":

Then

Break out of 1 loop(s).

End

Else

End

Span-8 Wash

Wash all probes of Pod2 at the closest wash station; do a passive wash by dispensing 1 mL

while in the wells and 2 mL to waste. Delay for 300 ms after each dispense.

Wash only the probes that have been used.

Script

Description:

Labware Pickup from TB1

Execute the following script code:

```
Option Explicit

' This script duplicates the functionality of the first half of the Move Labware step.

' Modified 3/7/2003 by MAG to lift the labware to a specific height off the deck

' (height measured at top of the labware) which was useful for a specific customer

' reading barcodes via their own reader, with labels always a certain distance from

' the top of the labware.


Set e = CreateObject("World.EngineObject")
```

```
Dim Source, Target, PodName
Dim Pod, CurDeck, OrigPodSpeed, LWSpeedLimit

' PodName        The name of the pod to use
PodName = StepDictionary.Let.PodName
if IsEmpty(PodName) or PodName = "" then err.raise 1050, , "PodName must be defined"
Set Pod = World.Devices.Pipettor1(PodName)
OrigPodSpeed = Pod.CurrentSpeed

Set CurDeck = World.Devices.Pipettor1.Deck

' Source         Name of the source position (string)
' Need to use Deck.FindLabwarePosition,
' in case labware is referred to by name instead of location
Source = CurDeck.FindLabwarePosition(e.EvaluateExpression(StepDictionary.Let.Source))
'tls.msg MFX.NextStep & " " & source
if IsEmpty(Source) or Source = "" then err.raise 1050, , "Source must be defined"

' Get the depth to grab a piece of labware at.
Dim Depth
Depth = e.EvaluateExpression(StepDictionary.Let.Depth)
if IsEmpty(Depth)         then err.raise 1050, , "Depth must be defined"
If Depth < 1              then err.raise 1050, , "Depth must be at least 1"

' Convert Source and Target names into objects
Dim Src
Set Src = Pipettor.Deck.Positions(Source)
```

```
' Get the Source Labware
Dim srcLabware
World.Volatile.Depth = Depth
Set srcLabware = Src.GetLabwareAtDepth(Depth)

' Get the source labware height  [DON'T NEED THIS ANYMORE]
' Dim srcLwHeight
' srcLwHeight = srcLabware.Class.Height

Dim Z
Z = CDbl(Src.Z(Pod))

' Get offsets from position to the labware at source position
Dim sx, sy, sz
sx = CDbl(Src.GetOneLabwareOffsetAtDepth(Depth, "X"))
sy = CDbl(Src.GetOneLabwareOffsetAtDepth(Depth, "Y"))
sz = CDbl(Src.GetOneLabwareOffsetAtDepth(Depth, "Z"))

pd = CDbl(Pod.VariantDictionary.GetDefault("GripperDOffset", 0.0))

' Get the labware gripper offsets and squeeze values
Dim gx, gy, gz, pd
Dim Squeeze, Unsqueeze
Select Case Pod.PodType
   Case "ATTILA"
      gx = CDbl(srcLabware.Class.GripperInfo.MultiChannel.GripperXOffset)
      gy = CDbl(srcLabware.Class.GripperInfo.MultiChannel.GripperYOffset)
      gz = CDbl(srcLabware.Class.GripperInfo.MultiChannel.GripperZOffset)
      Squeeze = CDbl(Pod.DAxisFromGripper(CDbl(pd) +
```

```
CDbl(srcLabware.Class.GripperInfo.MultiChannel.Squeeze)))
      Unsqueeze = CDbl(Pod.DAxisFromGripper(CDbl(pd) +
CDbl(srcLabware.Class.GripperInfo.MultiChannel.Unsqueeze)))
'tls.msg pod.podtype
'tls.msg gx & " " & gy & " " & gz & " " & Squeeze & " " & Unsqueeze
   Case Else ' need to fill in code for NX Gripper
End Select

' Get the Pod gripper offsets
Dim px, py, pz
px = CDbl(Pod.GripperXOffset)
py = CDbl(Pod.GripperYOffset)
pz = CDbl(Pod.GripperZOffset)

Dim LiftHeight, e

LiftHeight = e.EvaluateExpression(StepDictionary.Let.LiftHeight)

' Temporarily save the height of the move
World.Volatile.LiftHeight = LiftHeight

'Reserve the resources used, i.e., the source position and the Pod
World.Globals.ResourceReserver.ReserveResources Array(Src, Pod)

' Open the source position
Src.Open True, Depth

' Start Moving
```

```
Pod.ApproachPosition Source, sx+gx-px, sy+gy-py, Z + sz+gz-pz, False, Unsqueeze

' Extend the grippers
Pod.GripperExtend

' Grab Labware
Pod.AbsMove ,,,Squeeze

' Enforce the labware's speed limit
LWSpeedLimit = srcLabware.Class.SpeedLimit
If LWSpeedLimit < OrigPodSpeed Then Pod.SetSpeed LWSpeedLimit

' Notify software that the source labware is now in the grippers
' Version 2.x syntax: Pod.GetLabware Source, Depth
Pod.Gripper.GetLabwareObject Source, Depth

' Move to a safe height above the position (original function commented out)
' Pod.MoveToSafe

If LiftHeight < 0 Then err.raise 1050, ,  "Lift Height too low ; would result in downward
motion"

Pod.RelativeMoveAxes "Z", LiftHeight

' Close the source position
' Src.Close True, Depth

'Restore original pod speed now that the move is finished
Pod.SetSpeed OrigPodSpeed
```

Span-8 New Tips

Get new TB1 tips for probes 1-6 on Pod2.

Script

Description:

Labware Place to TB1

Execute the following script code:

```
Option Explicit

' This script duplicates the functionality of the Move Labware step.

' It has been modified to only place the labware after another step has picked it up.

Dim e

Set e = CreateObject("World.EngineObject")

Dim Target, PodName

Dim Pod, CurDeck, OrigPodSpeed, LWSpeedLimit


' PodName        The name of the pod to use

PodName = StepDictionary.Let.PodName

if IsEmpty(PodName) or PodName = "" then err.raise 1050, , "PodName must be defined"

Set Pod = World.Devices.Pipettor1(PodName)

OrigPodSpeed = Pod.CurrentSpeed


Set CurDeck = World.Devices.Pipettor1.Deck


' Target         Name of the target position (string)

' Need to use Deck.FindLabwarePosition,

' in case labware is referred to by name instead of location

Target = CurDeck.FindLabwarePosition(e.EvaluateExpression(StepDictionary.Let.Target))
```

```
if IsEmpty(Target) or Target = "" then err.raise 1050, , "Target must be defined"

If Not IsObject(Pod.GrippedLabware) Then err.raise 1050, , "There is no labware in the
gripper."
Dim glw
set glw = Pod.GrippedLabware(Pod.GrippedLabware.Count - 1) ' The gripped labware is an
array with the bottom piece at the highest index

' Convert Source and Target names into objects
Dim Dst
Set Dst = Pipettor.Deck.Positions(Target)

Dim Z
Dim sx, sy, sz
' Dim gx, gy, gz, pd
'pd = CDbl(Pod.VariantDictionary.GetDefault("GripperDOffset", 0.0))

' Get the squeeze values
'Dim Unsqueeze
'Unsqueeze = CDbl(Pod.DAxisFromGripper(CDbl(pd) + CDbl(glw.Class.Unsqueeze)))

' Get the Pod gripper offsets
Dim px, py, pz
px = CDbl(Pod.GripperXOffset)
py = CDbl(Pod.GripperYOffset)
pz = CDbl(Pod.GripperZOffset)

' Enforce the labware's speed limit
```

```
'LWSpeedLimit = srcLabware.Class.SpeedLimit
'If LWSpeedLimit < OrigPodSpeed Then Pod.SetSpeed LWSpeedLimit

'Get height of destination position
Z = CDbl(Dst.Z(Pod))

' Get offsets from position to the labware at target position
Dim tx, ty, tz, dx, dy, dz
tx = CDbl(Dst.GetOneLabwareOffsetAtDepth(1, "X"))
ty = CDbl(Dst.GetOneLabwareOffsetAtDepth(1, "Y"))
tz = CDbl(Dst.GetOneLabwareOffsetAtDepth(1, "Z"))

' Get the Target Labware
Dim dstLabware
dx = 0
dy = 0
dz = 0
dstLabware = Null
if (Dst.StackDepth > 0) then
  Set dstLabware = Dst.GetLabwareAtDepth(1)
end if

' Get Destination Stack Offsets (really Per-Labware Offsets)
dx = CDbl(Dst.GetOneStackOffset(glw, dstLabware, "X"))
dy = CDbl(Dst.GetOneStackOffset(glw, dstLabware, "Y"))
dz = CDbl(Dst.GetOneStackOffset(glw, dstLabware, "Z"))

' Get the labware gripper offsets (added-in by MAG 3/7/2003)
```

```
World.Volatile.MyDest = Dst


'Dim gx, gy, gz, pd

'gx = CDbl(glw.Class.GripperXOffset)

'gy = CDbl(glw.Class.GripperYOffset)

'gz = CDbl(glw.Class.GripperZOffset)


pd = CDbl(Pod.VariantDictionary.GetDefault("GripperDOffset", 0.0))


' Get the labware gripper offsets and squeeze values

Dim gx, gy, gz, pd

Dim Unsqueeze

Select Case Pod.PodType

   Case "ATTILA"

      gx = CDbl(glw.Class.GripperInfo.MultiChannel.GripperXOffset)

      gy = CDbl(glw.Class.GripperInfo.MultiChannel.GripperYOffset)

      gz = CDbl(glw.Class.GripperInfo.MultiChannel.GripperZOffset)

      Unsqueeze = CDbl(Pod.DAxisFromGripper(CDbl(pd) +
CDbl(glw.Class.GripperInfo.MultiChannel.Unsqueeze)))

'tls.msg pod.podtype

'tls.msg gx & " " & gy & " " & gz & " "  & " " & Unsqueeze

   Case Else ' need to fill in code for NX Gripper

End Select






' Open Destination

Dst.Open True, 0
```

```
' Start Moving

Pod.ApproachPosition Target, CDbl(tx+dx+gx-px), CDbl(ty+dy+gy-py), CDbl(Z + tz+dz+gz-pz),

True


Pod.MoveZ CDbl(Z + tz + dz + gz - pz)


' Put Labware

Pod.AbsMove ,,,Unsqueeze


' Notify software the labware that was in the grippers is now at the target

' old v. 2.x syntax Pod.PutLabware Target

Pod.Gripper.PutLabwareObject Target


' Retract grippers

Pod.GripperRetract


' Close the destination position

Dst.Close True, 0


'Restore original pod speed now that the move is finished

'Pod.SetSpeed OrigPodSpeed


' Move to a safe height

Pod.MoveToSafe

---------------------------------------------------------------------------

Script

Description:
```

Labware Pickup from Cryo1

Execute the following script code:

```
Option Explicit

' This script duplicates the functionality of the first half of the Move Labware step.

' Modified 3/7/2003 by MAG to lift the labware to a specific height off the deck

' (height measured at top of the labware) which was useful for a specific customer

' reading barcodes via their own reader, with labels always a certain distance from

' the top of the labware.


Set e = CreateObject("World.EngineObject")


Dim Source, Target, PodName

Dim Pod, CurDeck, OrigPodSpeed, LWSpeedLimit


' PodName        The name of the pod to use

PodName = StepDictionary.Let.PodName

if IsEmpty(PodName) or PodName = "" then err.raise 1050, , "PodName must be defined"

Set Pod = World.Devices.Pipettor1(PodName)

OrigPodSpeed = Pod.CurrentSpeed


Set CurDeck = World.Devices.Pipettor1.Deck


' Source         Name of the source position (string)

' Need to use Deck.FindLabwarePosition,

' in case labware is referred to by name instead of location

Source = CurDeck.FindLabwarePosition(e.EvaluateExpression(StepDictionary.Let.Source))

'tls.msg MFX.NextStep & " " & source

if IsEmpty(Source) or Source = "" then err.raise 1050, , "Source must be defined"
```

```
' Get the depth to grab a piece of labware at.
Dim Depth
Depth = e.EvaluateExpression(StepDictionary.Let.Depth)
if IsEmpty(Depth)         then err.raise 1050, , "Depth must be defined"
If Depth < 1              then err.raise 1050, , "Depth must be at least 1"

' Convert Source and Target names into objects
Dim Src
Set Src = Pipettor.Deck.Positions(Source)

' Get the Source Labware
Dim srcLabware
World.Volatile.Depth = Depth
Set srcLabware = Src.GetLabwareAtDepth(Depth)

' Get the source labware height  [DON'T NEED THIS ANYMORE]
' Dim srcLwHeight
' srcLwHeight = srcLabware.Class.Height

Dim Z
Z = CDbl(Src.Z(Pod))

' Get offsets from position to the labware at source position
Dim sx, sy, sz
sx = CDbl(Src.GetOneLabwareOffsetAtDepth(Depth, "X"))
sy = CDbl(Src.GetOneLabwareOffsetAtDepth(Depth, "Y"))
sz = CDbl(Src.GetOneLabwareOffsetAtDepth(Depth, "Z"))
```

```
pd = CDbl(Pod.VariantDictionary.GetDefault("GripperDOffset", 0.0))

' Get the labware gripper offsets and squeeze values
Dim gx, gy, gz, pd
Dim Squeeze, Unsqueeze
Select Case Pod.PodType
   Case "ATTILA"
      gx = CDbl(srcLabware.Class.GripperInfo.MultiChannel.GripperXOffset)
      gy = CDbl(srcLabware.Class.GripperInfo.MultiChannel.GripperYOffset)
      gz = CDbl(srcLabware.Class.GripperInfo.MultiChannel.GripperZOffset)
      Squeeze = CDbl(Pod.DAxisFromGripper(CDbl(pd) +
CDbl(srcLabware.Class.GripperInfo.MultiChannel.Squeeze)))
      Unsqueeze = CDbl(Pod.DAxisFromGripper(CDbl(pd) +
CDbl(srcLabware.Class.GripperInfo.MultiChannel.Unsqueeze)))
'tls.msg pod.podtype
'tls.msg gx & " " & gy & " " & gz & " " & Squeeze & " " & Unsqueeze
   Case Else ' need to fill in code for NX Gripper
End Select

' Get the Pod gripper offsets
Dim px, py, pz
px = CDbl(Pod.GripperXOffset)
py = CDbl(Pod.GripperYOffset)
pz = CDbl(Pod.GripperZOffset)

Dim LiftHeight, e

LiftHeight = e.EvaluateExpression(StepDictionary.Let.LiftHeight)
```

```
' Temporarily save the height of the move

World.Volatile.LiftHeight = LiftHeight


'Reserve the resources used, i.e., the source position and the Pod

World.Globals.ResourceReserver.ReserveResources Array(Src, Pod)


' Open the source position

Src.Open True, Depth


' Start Moving

Pod.ApproachPosition Source, sx+gx-px, sy+gy-py, Z + sz+gz-pz, False, Unsqueeze


' Extend the grippers

Pod.GripperExtend


' Grab Labware

Pod.AbsMove ,,,Squeeze


' Enforce the labware's speed limit

LWSpeedLimit = srcLabware.Class.SpeedLimit

If LWSpeedLimit < OrigPodSpeed Then Pod.SetSpeed LWSpeedLimit


' Notify software that the source labware is now in the grippers

' Version 2.x syntax: Pod.GetLabware Source, Depth

Pod.Gripper.GetLabwareObject Source, Depth


' Move to a safe height above the position (original function commented out)

' Pod.MoveToSafe
```

```
If LiftHeight < 0 Then err.raise 1050, ,  "Lift Height too low ; would result in downward
motion"

Pod.RelativeMoveAxes "Z", LiftHeight

' Close the source position
' Src.Close True, Depth

'Restore original pod speed now that the move is finished
Pod.SetSpeed OrigPodSpeed
```

RotatingALP

  Position: R1

  Rotate to Orientation: Portrait (90∞ counterclockwise)

Configure Beads Step

Bitmap: C:\Program Files\Biomek Software\Bitmaps\Vials.bmp

Description:

Caption: Configure Beads Step

```
Code: ' DispHeightBeads
Set LW = Positions(Labware.Location("Cryo1")).Labware

DispHeightCM = LW.HeightFromVolume(MFX.P1VolBlood, (1+RoundsCompleted))

' start dispensing at 2 mm below liqlevel
Extend "ZDispBeads", (DispHeightCM * 10) - 2
```

Prompt: [IDispatch]

Tooltip: Scripted Let

Transfer

Using Pod2, execute the following transfer:

From: Cryo2, with the following pattern:

```
   1  2  3  4  5  6
 A () () () () () ()
 B () () () () () ()
 C () () () () () ()
 D () () () >< () ()
```

, Water

Proceed down first, then left to right.

Start from the beginning of the selection.

Set the mark at the last well transferred.

Use the following custom technique:

Use the following pipetting template: Span-8 P1000 2

Calibration Offset: 0

Calibration Slope: 1

Minimum Pipetting Height: 0.5 mm

Prewet: False

Aspirate Blowout: True

Follow Liquid: False

Height: -2 mm from the liquid

Mix: True

Mix Aspirate Speed: 300µL/s

Mix Aspirate Height: 1 mm from the liquid

Mix Dispense Speed: 300µL/s

Mix Dispense Height: 1 mm from the liquid

Mix Count: 2

Mix Volume: 200 µL

Operation speed: 40µL/s

Tip Touch: False

Trailing Air Gap: False

Override Liquid Type Settings

Aspirate Delay: 10 ms

Aspirate Speed: 100 µL/s

Blowout Delay: 0 ms

Blowout Volume: 20 µL

Dispense Delay: 0 ms

Dispense Speed: 100 µL/s

Prewet Delay: 0 ms

Prewet Overage: 0 µL

Tip Touch Delay: 0 ms

Tip Touch Speed: 50 µL/s

Trailing Air Gap Volume: 1 µL

Override the technique height by moving to 0.5 mm from the bottom.

To: Cryo1, =MFX.P2VolBeadsµL, sections specified by "=ColWells()(1+RoundsCompleted)",

Water

Proceed down first, then left to right.

Start from the beginning of the selection.

Set the mark at the last well transferred.

Use the following custom technique:

Use the following pipetting template: Span-8 MultiDispense

Calibration Offset: 0

Calibration Slope: 1

Minimum Pipetting Height: 1.5 mm

Prewet: False

Blowout: True

Follow Liquid: False

Height: 1.5 mm from the bottom

Mix: False

Mix Aspirate Speed: 100µL/s

Mix Aspirate Height: 0 mm from the liquid

Mix Dispense Speed: 100µL/s

Mix Dispense Height: 0 mm from the liquid

Mix Count: 1

Mix Volume: 10 µL

Operation speed: 40µL/s

Tip Touch: False

Override Liquid Type Settings

Aspirate Delay: 10 ms

Aspirate Speed: 50 µL/s

Blowout Delay: 0 ms

Blowout Volume: 20 µL

Dispense Delay: 0 ms

Dispense Speed: 25 µL/s

Prewet Delay: 0 ms

Prewet Overage: 0 µL

Tip Touch Delay: 0 ms

Tip Touch Speed: 50 µL/s

Trailing Air Gap Volume: 1 µL

Override the technique height by moving to =ZDispBeads mm from the bottom.

Dispense up to 1 time(s) per draw.

Create 1 replicate(s) of each source well.

Keep the current tips

Unload the tips when finished.

Stop when finished with Destinations.

Probes 1-6 will be used.

End Scripted Let

RotatingALP

  Position: R1

  Rotate to Orientation: Landscape

Script

Description:

Labware Place to Cryo1

Execute the following script code:

```
Option Explicit

' This script duplicates the functionality of the Move Labware step.

' It has been modified to only place the labware after another step has picked it up.

Dim e

Set e = CreateObject("World.EngineObject")

Dim Target, PodName

Dim Pod, CurDeck, OrigPodSpeed, LWSpeedLimit


' PodName        The name of the pod to use

PodName = StepDictionary.Let.PodName

if IsEmpty(PodName) or PodName = "" then err.raise 1050, , "PodName must be defined"

Set Pod = World.Devices.Pipettor1(PodName)

OrigPodSpeed = Pod.CurrentSpeed
```

```
Set CurDeck = World.Devices.Pipettor1.Deck

' Target         Name of the target position (string)
' Need to use Deck.FindLabwarePosition,
' in case labware is referred to by name instead of location
Target = CurDeck.FindLabwarePosition(e.EvaluateExpression(StepDictionary.Let.Target))

if IsEmpty(Target) or Target = "" then err.raise 1050, , "Target must be defined"

If Not IsObject(Pod.GrippedLabware) Then err.raise 1050, , "There is no labware in the
gripper."
Dim glw
set glw = Pod.GrippedLabware(Pod.GrippedLabware.Count - 1) ' The gripped labware is an
array with the bottom piece at the highest index

' Convert Source and Target names into objects
Dim Dst
Set Dst = Pipettor.Deck.Positions(Target)

Dim Z
Dim sx, sy, sz
' Dim gx, gy, gz, pd
'pd = CDbl(Pod.VariantDictionary.GetDefault("GripperDOffset", 0.0))

' Get the squeeze values
'Dim Unsqueeze
'Unsqueeze = CDbl(Pod.DAxisFromGripper(CDbl(pd) + CDbl(glw.Class.Unsqueeze)))
```

```
' Get the Pod gripper offsets
Dim px, py, pz
px = CDbl(Pod.GripperXOffset)
py = CDbl(Pod.GripperYOffset)
pz = CDbl(Pod.GripperZOffset)

' Enforce the labware's speed limit
'LWSpeedLimit = srcLabware.Class.SpeedLimit
'If LWSpeedLimit < OrigPodSpeed Then Pod.SetSpeed LWSpeedLimit

'Get height of destination position
Z = CDbl(Dst.Z(Pod))

' Get offsets from position to the labware at target position
Dim tx, ty, tz, dx, dy, dz
tx = CDbl(Dst.GetOneLabwareOffsetAtDepth(1, "X"))
ty = CDbl(Dst.GetOneLabwareOffsetAtDepth(1, "Y"))
tz = CDbl(Dst.GetOneLabwareOffsetAtDepth(1, "Z"))

' Get the Target Labware
Dim dstLabware
dx = 0
dy = 0
dz = 0
dstLabware = Null
if (Dst.StackDepth > 0) then
  Set dstLabware = Dst.GetLabwareAtDepth(1)
end if
```

```
' Get Destination Stack Offsets (really Per-Labware Offsets)
dx = CDbl(Dst.GetOneStackOffset(glw, dstLabware, "X"))
dy = CDbl(Dst.GetOneStackOffset(glw, dstLabware, "Y"))
dz = CDbl(Dst.GetOneStackOffset(glw, dstLabware, "Z"))

' Get the labware gripper offsets (added-in by MAG 3/7/2003)
World.Volatile.MyDest = Dst

'Dim gx, gy, gz, pd
'gx = CDbl(glw.Class.GripperXOffset)
'gy = CDbl(glw.Class.GripperYOffset)
'gz = CDbl(glw.Class.GripperZOffset)

pd = CDbl(Pod.VariantDictionary.GetDefault("GripperDOffset", 0.0))

' Get the labware gripper offsets and squeeze values
Dim gx, gy, gz, pd
Dim Unsqueeze
Select Case Pod.PodType
   Case "ATTILA"
      gx = CDbl(glw.Class.GripperInfo.MultiChannel.GripperXOffset)
      gy = CDbl(glw.Class.GripperInfo.MultiChannel.GripperYOffset)
      gz = CDbl(glw.Class.GripperInfo.MultiChannel.GripperZOffset)
      Unsqueeze = CDbl(Pod.DAxisFromGripper(CDbl(pd) +
CDbl(glw.Class.GripperInfo.MultiChannel.Unsqueeze)))
'tls.msg pod.podtype
'tls.msg gx & " " & gy & " " & gz & " "  & " " & Unsqueeze
```

```
   Case Else ' need to fill in code for NX Gripper
End Select

' Open Destination
Dst.Open True, 0

' Start Moving
Pod.ApproachPosition Target, CDbl(tx+dx+gx-px), CDbl(ty+dy+gy-py), CDbl(Z + tz+dz+gz-pz),
True

Pod.MoveZ CDbl(Z + tz + dz + gz - pz)

' Put Labware
Pod.AbsMove ,,,Unsqueeze

' Notify software the labware that was in the grippers is now at the target
' old v. 2.x syntax Pod.PutLabware Target
Pod.Gripper.PutLabwareObject Target

' Retract grippers
Pod.GripperRetract

' Close the destination position
Dst.Close True, 0

'Restore original pod speed now that the move is finished
```

```
'Pod.SetSpeed OrigPodSpeed


' Move to a safe height

Pod.MoveToSafe
```

Run setstepdone

  wait = true

End Loop

Move Labware

Move the top "1" plates at "StoreLid" to "Cryo2" using pod "Pod1".

End

Else

End

End Group

Group

If

If "MFX.NextStep = 3":

Then

Move To

Move "Pod2" to deck position "W1" at offset (0,0).

Move Labware

Move the top "2" plates at "Cryo1" to "Orbital1" using pod "Pod1".

Move To

Move "Pod1" to deck position "P2" at offset (0,0).

Run setstepdone

  wait = true

End

Else

End

Loop

Loop from "=MFX.RoundsCompleted(4)" to "=MFX.TotalRounds(4)", incrementing by "1".

If

If "MFX.NextStep = 4":

Then

Script

Description:

Labware Pickup from Cryo1

Execute the following script code:

```
Option Explicit

' This script duplicates the functionality of the first half of the Move Labware step.

' Modified 3/7/2003 by MAG to lift the labware to a specific height off the deck

' (height measured at top of the labware) which was useful for a specific customer

' reading barcodes via their own reader, with labels always a certain distance from

' the top of the labware.


Set e = CreateObject("World.EngineObject")


Dim Source, Target, PodName

Dim Pod, CurDeck, OrigPodSpeed, LWSpeedLimit


' PodName        The name of the pod to use

PodName = StepDictionary.Let.PodName

if IsEmpty(PodName) or PodName = "" then err.raise 1050, , "PodName must be defined"

Set Pod = World.Devices.Pipettor1(PodName)

OrigPodSpeed = Pod.CurrentSpeed


Set CurDeck = World.Devices.Pipettor1.Deck


' Source         Name of the source position (string)

' Need to use Deck.FindLabwarePosition,

' in case labware is referred to by name instead of location

Source = CurDeck.FindLabwarePosition(e.EvaluateExpression(StepDictionary.Let.Source))

'tls.msg MFX.NextStep & " " & source

if IsEmpty(Source) or Source = "" then err.raise 1050, , "Source must be defined"
```

```
' Get the depth to grab a piece of labware at.
Dim Depth
Depth = e.EvaluateExpression(StepDictionary.Let.Depth)
if IsEmpty(Depth)         then err.raise 1050, , "Depth must be defined"
If Depth < 1              then err.raise 1050, , "Depth must be at least 1"

' Convert Source and Target names into objects
Dim Src
Set Src = Pipettor.Deck.Positions(Source)

' Get the Source Labware
Dim srcLabware
World.Volatile.Depth = Depth
Set srcLabware = Src.GetLabwareAtDepth(Depth)

' Get the source labware height  [DON'T NEED THIS ANYMORE]
' Dim srcLwHeight
' srcLwHeight = srcLabware.Class.Height

Dim Z
Z = CDbl(Src.Z(Pod))

' Get offsets from position to the labware at source position
Dim sx, sy, sz
sx = CDbl(Src.GetOneLabwareOffsetAtDepth(Depth, "X"))
sy = CDbl(Src.GetOneLabwareOffsetAtDepth(Depth, "Y"))
sz = CDbl(Src.GetOneLabwareOffsetAtDepth(Depth, "Z"))

pd = CDbl(Pod.VariantDictionary.GetDefault("GripperDOffset", 0.0))
```

```
' Get the labware gripper offsets and squeeze values
Dim gx, gy, gz, pd
Dim Squeeze, Unsqueeze
Select Case Pod.PodType
   Case "ATTILA"
      gx = CDbl(srcLabware.Class.GripperInfo.MultiChannel.GripperXOffset)
      gy = CDbl(srcLabware.Class.GripperInfo.MultiChannel.GripperYOffset)
      gz = CDbl(srcLabware.Class.GripperInfo.MultiChannel.GripperZOffset)
      Squeeze = CDbl(Pod.DAxisFromGripper(CDbl(pd) +
CDbl(srcLabware.Class.GripperInfo.MultiChannel.Squeeze)))
      Unsqueeze = CDbl(Pod.DAxisFromGripper(CDbl(pd) +
CDbl(srcLabware.Class.GripperInfo.MultiChannel.Unsqueeze)))
'tls.msg pod.podtype
'tls.msg gx & " " & gy & " " & gz & " " & Squeeze & " " & Unsqueeze
   Case Else ' need to fill in code for NX Gripper
End Select

' Get the Pod gripper offsets
Dim px, py, pz
px = CDbl(Pod.GripperXOffset)
py = CDbl(Pod.GripperYOffset)
pz = CDbl(Pod.GripperZOffset)

Dim LiftHeight, e

LiftHeight = e.EvaluateExpression(StepDictionary.Let.LiftHeight)
```

```
' Temporarily save the height of the move
World.Volatile.LiftHeight = LiftHeight

'Reserve the resources used, i.e., the source position and the Pod
World.Globals.ResourceReserver.ReserveResources Array(Src, Pod)

' Open the source position
Src.Open True, Depth

' Start Moving
Pod.ApproachPosition Source, sx+gx-px, sy+gy-py, Z + sz+gz-pz, False, Unsqueeze

' Extend the grippers
Pod.GripperExtend

' Grab Labware
Pod.AbsMove ,,,Squeeze

' Enforce the labware's speed limit
LWSpeedLimit = srcLabware.Class.SpeedLimit
If LWSpeedLimit < OrigPodSpeed Then Pod.SetSpeed LWSpeedLimit

' Notify software that the source labware is now in the grippers
' Version 2.x syntax: Pod.GetLabware Source, Depth
Pod.Gripper.GetLabwareObject Source, Depth

' Move to a safe height above the position (original function commented out)
' Pod.MoveToSafe
```

```
If LiftHeight < 0 Then err.raise 1050, ,  "Lift Height too low ; would result in downward
motion"

Pod.RelativeMoveAxes "Z", LiftHeight

' Close the source position
' Src.Close True, Depth

'Restore original pod speed now that the move is finished
Pod.SetSpeed OrigPodSpeed
---------------------------------------------------------------------------
Run getnewtips
  pattern = =TwoTips()(MFX.NumSamples)
---------------------------------------------------------------------------
Run starttimer
  index = =""
  timername = ="Shaking/Incubating"
  waittime = =MFX.P4ShakeTime
---------------------------------------------------------------------------
Configure
Bitmap: C:\Program Files\Biomek Software\Bitmaps\Vials.bmp
Description:
Caption: Configure
Code: Set LW = Positions(Labware.Location("Cryo1")).Labware
LW.ConfigureAmounts VolMinSB

' calculate the asp & dispense height for the mix step
' so we don't need to use LLS
```

```
' assume it's the same for all wells

AspHeightCM = LW.HeightFromVolume(VolMinSB, 1)
' this is the height in CM
' needs to be multiplied by 10 because the step expects MM

Vol = VolMinSB - MFX.P4MixVolume ' this is the volume
                                 ' that is left in the well
                                 ' after the aspiration

DispHeightCM = LW.HeightFromVolume(Vol, 1)

Extend "ZAsp",  AspHeightCM * 10 ' convert cm to mm
Extend "ZDisp", 0.6  'DispHeightCM * 10 'was 0.2 before ver. 0.07
Extend "Delay", 0
Prompt: [IDispatch]
Tooltip: Scripted Let
---------------------------------------------------------------------------
Transfer
Using Pod2, execute the following transfer:
From: Cryo1, sections specified by "=Wells()(MFX.NumSamples)", BeadsplusCells
Proceed down first, then left to right.
Start from the beginning of the selection.
Do not set the mark.
Use the following custom technique:
  Use the following pipetting template: OS Span-8 P1000 LongTravelMix 2
  Calibration Offset: 2.54
  Calibration Slope: 1.033
  Minimum Pipetting Height: =0.5 mm
```

Prewet: False

Aspirate Blowout: True

Follow Liquid: False

Height: 0 mm from the liquid

Mix: True

Mix Aspirate Speed: =MFX.P4MixSpeedµL/s

Mix Aspirate Height: =ZAsp mm from the liquid

Mix Dispense Speed: =MFX.P4MixSpeedµL/s

Mix Dispense Height: =ZDisp mm from the liquid

Mix Count: =MFX.P4MixCycles

Mix Volume: =MFX.P4MixVolume µL

Operation speed: 70µL/s

Tip Touch: True

Trailing Air Gap: False

Override Liquid Type Settings

Aspirate Delay: 10 ms

Aspirate Speed: 300 µL/s

Blowout Delay: =Delay ms

Blowout Volume: 20 µL

Dispense Delay: 20 ms

Dispense Speed: 200 µL/s

Prewet Delay: 200 ms

Prewet Overage: 0 µL

Tip Touch Delay: 300 ms

Tip Touch Speed: 70 µL/s

Trailing Air Gap Volume: 10 µL

Override the technique height by moving to =ZAsp mm from the bottom.

To: Cryo1, 0µL, sections specified by "=Wells()(MFX.NumSamples)", BeadsplusCells

Proceed down first, then left to right.

Start from the beginning of the selection.

Do not set the mark.

Use the following custom technique:

  Use the following pipetting template: OS Span-8 P1000 MixOnly 2

  Calibration Offset: 2.54

  Calibration Slope: 1.033

  Minimum Pipetting Height: =0.5 mm

  Prewet: False

  Blowout: False

  Follow Liquid: False

  Height: 0 mm from the liquid

  Mix: False

  Mix Aspirate Speed: 100µL/s

  Mix Aspirate Height: 0 mm from the liquid

  Mix Dispense Speed: 100µL/s

  Mix Dispense Height: 0 mm from the liquid

  Mix Count: 1

  Mix Volume: 900 µL

  Operation speed: 70µL/s

  Tip Touch: False

  Override Liquid Type Settings

    Aspirate Delay: 10 ms

    Aspirate Speed: 300 µL/s

    Blowout Delay: 10 ms

    Blowout Volume: 20 µL

    Dispense Delay: 20 ms

    Dispense Speed: 200 µL/s

    Prewet Delay: 0 ms

    Prewet Overage: 0 µL

    Tip Touch Delay: 300 ms

    Tip Touch Speed: 70 µL/s

    Trailing Air Gap Volume: 10 µL

Override the technique height by moving to =ZDisp mm from the bottom.

Dispense up to 1 time(s) per draw.

Create 1 replicate(s) of each source well.

Keep the current tips

Keep the tips when finished.

Stop when finished with Destinations.

Probes specified by "=OneTip()(MFX.NumSamples)" will be used.

End Scripted Let

Script

Description:

Labware Place to Cryo1

Execute the following script code:

```
Option Explicit

' This script duplicates the functionality of the Move Labware step.

' It has been modified to only place the labware after another step has picked it up.

Dim e

Set e = CreateObject("World.EngineObject")

Dim Target, PodName

Dim Pod, CurDeck, OrigPodSpeed, LWSpeedLimit
```

```
' PodName        The name of the pod to use
PodName = StepDictionary.Let.PodName
if IsEmpty(PodName) or PodName = "" then err.raise 1050, , "PodName must be defined"
Set Pod = World.Devices.Pipettor1(PodName)
OrigPodSpeed = Pod.CurrentSpeed

Set CurDeck = World.Devices.Pipettor1.Deck

' Target         Name of the target position (string)
' Need to use Deck.FindLabwarePosition,
' in case labware is referred to by name instead of location
Target = CurDeck.FindLabwarePosition(e.EvaluateExpression(StepDictionary.Let.Target))

if IsEmpty(Target) or Target = "" then err.raise 1050, , "Target must be defined"

If Not IsObject(Pod.GrippedLabware) Then err.raise 1050, , "There is no labware in the
gripper."
Dim glw
set glw = Pod.GrippedLabware(Pod.GrippedLabware.Count - 1) ' The gripped labware is an
array with the bottom piece at the highest index

' Convert Source and Target names into objects
Dim Dst
Set Dst = Pipettor.Deck.Positions(Target)

Dim Z
Dim sx, sy, sz
' Dim gx, gy, gz, pd
'pd = CDbl(Pod.VariantDictionary.GetDefault("GripperDOffset", 0.0))
```

```
' Get the squeeze values
'Dim Unsqueeze
'Unsqueeze = CDbl(Pod.DAxisFromGripper(CDbl(pd) + CDbl(glw.Class.Unsqueeze)))

' Get the Pod gripper offsets
Dim px, py, pz
px = CDbl(Pod.GripperXOffset)
py = CDbl(Pod.GripperYOffset)
pz = CDbl(Pod.GripperZOffset)

' Enforce the labware's speed limit
'LWSpeedLimit = srcLabware.Class.SpeedLimit
'If LWSpeedLimit < OrigPodSpeed Then Pod.SetSpeed LWSpeedLimit

'Get height of destination position
Z = CDbl(Dst.Z(Pod))

' Get offsets from position to the labware at target position
Dim tx, ty, tz, dx, dy, dz
tx = CDbl(Dst.GetOneLabwareOffsetAtDepth(1, "X"))
ty = CDbl(Dst.GetOneLabwareOffsetAtDepth(1, "Y"))
tz = CDbl(Dst.GetOneLabwareOffsetAtDepth(1, "Z"))

' Get the Target Labware
Dim dstLabware
dx = 0
dy = 0
```

```
dz = 0

dstLabware = Null

if (Dst.StackDepth > 0) then

  Set dstLabware = Dst.GetLabwareAtDepth(1)

end if


' Get Destination Stack Offsets (really Per-Labware Offsets)

dx = CDbl(Dst.GetOneStackOffset(glw, dstLabware, "X"))

dy = CDbl(Dst.GetOneStackOffset(glw, dstLabware, "Y"))

dz = CDbl(Dst.GetOneStackOffset(glw, dstLabware, "Z"))


' Get the labware gripper offsets (added-in by MAG 3/7/2003)

World.Volatile.MyDest = Dst


'Dim gx, gy, gz, pd

'gx = CDbl(glw.Class.GripperXOffset)

'gy = CDbl(glw.Class.GripperYOffset)

'gz = CDbl(glw.Class.GripperZOffset)


pd = CDbl(Pod.VariantDictionary.GetDefault("GripperDOffset", 0.0))


' Get the labware gripper offsets and squeeze values

Dim gx, gy, gz, pd

Dim Unsqueeze

Select Case Pod.PodType

   Case "ATTILA"

      gx = CDbl(glw.Class.GripperInfo.MultiChannel.GripperXOffset)

      gy = CDbl(glw.Class.GripperInfo.MultiChannel.GripperYOffset)

      gz = CDbl(glw.Class.GripperInfo.MultiChannel.GripperZOffset)
```

```
      Unsqueeze = CDbl(Pod.DAxisFromGripper(CDbl(pd) +
CDbl(glw.Class.GripperInfo.MultiChannel.Unsqueeze)))
'tls.msg pod.podtype
'tls.msg gx & " " & gy & " " & gz & " "  & " " & Unsqueeze
   Case Else ' need to fill in code for NX Gripper
End Select




' Open Destination
Dst.Open True, 0

' Start Moving
Pod.ApproachPosition Target, CDbl(tx+dx+gx-px), CDbl(ty+dy+gy-py), CDbl(Z + tz+dz+gz-pz),
True

Pod.MoveZ CDbl(Z + tz + dz + gz - pz)

' Put Labware
Pod.AbsMove ,,,Unsqueeze

' Notify software the labware that was in the grippers is now at the target
' old v. 2.x syntax Pod.PutLabware Target
Pod.Gripper.PutLabwareObject Target

' Retract grippers
Pod.GripperRetract
```

```
' Close the destination position
Dst.Close True, 0

'Restore original pod speed now that the move is finished
'Pod.SetSpeed OrigPodSpeed

' Move to a safe height
Pod.MoveToSafe
---------------------------------------------------------------------------
Run parkinwashstation
  fromtop = -0.2
  rule = 3
  withtipwash = False
---------------------------------------------------------------------------
Run setstepdone
  wait = true
---------------------------------------------------------------------------
End
---------------------------------------------------------------------------
Else
---------------------------------------------------------------------------
End
---------------------------------------------------------------------------
If
If "MFX.NextStep = 5":
---------------------------------------------------------------------------
Then
---------------------------------------------------------------------------
```

Run starttimer

  index = =""

  timername = ="Shaking/Incubating"

  waittime = =MFX.P4ShakeTime

Device Action

Send the following command to "OrbitalShakerALP0": "Shake".

Parameters: =MFX.P4ShakerSpeed, 1, CounterClockwise

Run waittimer

  index = =""

  settimer = False

  timername = ="Shaking/Incubating"

  waittime = ""

Device Action

Send the following command to "OrbitalShakerALP0": "Stop".

Parameters: 1

Run setstepdone

  wait = true

End

Else

Run waittimer

  index = =""

```
  settimer = False

  timername = ="Shaking/Incubating"

  waittime = ""
--------------------------------------------------------------------------
End
--------------------------------------------------------------------------
End Loop
--------------------------------------------------------------------------
If

If "MFX.UsePeltier":
--------------------------------------------------------------------------
Then
--------------------------------------------------------------------------
TShake1: Initialize
--------------------------------------------------------------------------
End
--------------------------------------------------------------------------
Else
--------------------------------------------------------------------------
End
--------------------------------------------------------------------------
End Group
--------------------------------------------------------------------------
Group
--------------------------------------------------------------------------
If

If "MFX.NextStep = 6":
--------------------------------------------------------------------------
Then
```

Move To

Move "Pod2" to deck position "W1" at offset (0,0).

Move Labware

Move the top "2" plates at "Cryo1" to "Pelt1" using pod "Pod1".

Move To

Move "Pod1" to deck position "P2" at offset (0,0).

Run parkinwashstation

  rule = 3

  withtipwash = False

Run setstepdone

  wait = true

End

Else

End

If

If "MFX.NextStep = 7":

Then

```
Run waittimer

  index = =""

  settimer = = True

  timername = ="Settling..."

  waittime = =SettleTimeBlood

---------------------------------------------------------------------------

Run setstepdone

  wait = true

---------------------------------------------------------------------------

End

---------------------------------------------------------------------------

Else

---------------------------------------------------------------------------

End

---------------------------------------------------------------------------

If

If "MFX.NextStep = 8":

---------------------------------------------------------------------------

Then

---------------------------------------------------------------------------

Script

Description:

Labware Pickup from Cryo1

Execute the following script code:

Option Explicit

' This script duplicates the functionality of the first half of the Move Labware step.

' Modified 3/7/2003 by MAG to lift the labware to a specific height off the deck

' (height measured at top of the labware) which was useful for a specific customer

' reading barcodes via their own reader, with labels always a certain distance from
```

```
' the top of the labware.

Set e = CreateObject("World.EngineObject")

Dim Source, Target, PodName
Dim Pod, CurDeck, OrigPodSpeed, LWSpeedLimit

' PodName        The name of the pod to use
PodName = StepDictionary.Let.PodName
if IsEmpty(PodName) or PodName = "" then err.raise 1050, , "PodName must be defined"
Set Pod = World.Devices.Pipettor1(PodName)
OrigPodSpeed = Pod.CurrentSpeed

Set CurDeck = World.Devices.Pipettor1.Deck

' Source         Name of the source position (string)
' Need to use Deck.FindLabwarePosition,
' in case labware is referred to by name instead of location
Source = CurDeck.FindLabwarePosition(e.EvaluateExpression(StepDictionary.Let.Source))
'tls.msg MFX.NextStep & " " & source
if IsEmpty(Source) or Source = "" then err.raise 1050, , "Source must be defined"

' Get the depth to grab a piece of labware at.
Dim Depth
Depth = e.EvaluateExpression(StepDictionary.Let.Depth)
if IsEmpty(Depth)         then err.raise 1050, , "Depth must be defined"
If Depth < 1              then err.raise 1050, , "Depth must be at least 1"
```

```
' Convert Source and Target names into objects
Dim Src
Set Src = Pipettor.Deck.Positions(Source)

' Get the Source Labware
Dim srcLabware
World.Volatile.Depth = Depth
Set srcLabware = Src.GetLabwareAtDepth(Depth)

' Get the source labware height  [DON'T NEED THIS ANYMORE]
' Dim srcLwHeight
' srcLwHeight = srcLabware.Class.Height

Dim Z
Z = CDbl(Src.Z(Pod))

' Get offsets from position to the labware at source position
Dim sx, sy, sz
sx = CDbl(Src.GetOneLabwareOffsetAtDepth(Depth, "X"))
sy = CDbl(Src.GetOneLabwareOffsetAtDepth(Depth, "Y"))
sz = CDbl(Src.GetOneLabwareOffsetAtDepth(Depth, "Z"))

pd = CDbl(Pod.VariantDictionary.GetDefault("GripperDOffset", 0.0))

' Get the labware gripper offsets and squeeze values
Dim gx, gy, gz, pd
Dim Squeeze, Unsqueeze
Select Case Pod.PodType
   Case "ATTILA"
```

```
      gx = CDbl(srcLabware.Class.GripperInfo.MultiChannel.GripperXOffset)

      gy = CDbl(srcLabware.Class.GripperInfo.MultiChannel.GripperYOffset)

      gz = CDbl(srcLabware.Class.GripperInfo.MultiChannel.GripperZOffset)

      Squeeze = CDbl(Pod.DAxisFromGripper(CDbl(pd) +
CDbl(srcLabware.Class.GripperInfo.MultiChannel.Squeeze)))

      Unsqueeze = CDbl(Pod.DAxisFromGripper(CDbl(pd) +
CDbl(srcLabware.Class.GripperInfo.MultiChannel.Unsqueeze)))

'tls.msg pod.podtype

'tls.msg gx & " " & gy & " " & gz & " " & Squeeze & " " & Unsqueeze

   Case Else ' need to fill in code for NX Gripper

End Select


' Get the Pod gripper offsets

Dim px, py, pz

px = CDbl(Pod.GripperXOffset)

py = CDbl(Pod.GripperYOffset)

pz = CDbl(Pod.GripperZOffset)


Dim LiftHeight, e


LiftHeight = e.EvaluateExpression(StepDictionary.Let.LiftHeight)


' Temporarily save the height of the move

World.Volatile.LiftHeight = LiftHeight


'Reserve the resources used, i.e., the source position and the Pod

World.Globals.ResourceReserver.ReserveResources Array(Src, Pod)
```

```
' Open the source position
Src.Open True, Depth

' Start Moving
Pod.ApproachPosition Source, sx+gx-px, sy+gy-py, Z + sz+gz-pz, False, Unsqueeze

' Extend the grippers
Pod.GripperExtend

' Grab Labware
Pod.AbsMove ,,,Squeeze

' Enforce the labware's speed limit
LWSpeedLimit = srcLabware.Class.SpeedLimit
If LWSpeedLimit < OrigPodSpeed Then Pod.SetSpeed LWSpeedLimit

' Notify software that the source labware is now in the grippers
' Version 2.x syntax: Pod.GetLabware Source, Depth
Pod.Gripper.GetLabwareObject Source, Depth

' Move to a safe height above the position (original function commented out)
' Pod.MoveToSafe

If LiftHeight < 0 Then err.raise 1050, ,  "Lift Height too low ; would result in downward
motion"

Pod.RelativeMoveAxes "Z", LiftHeight

' Close the source position
```

```
' Src.Close True, Depth


'Restore original pod speed now that the move is finished

Pod.SetSpeed OrigPodSpeed

---------------------------------------------------------------------------

Run getnewtips

  pattern = =TwoTips()(MFX.NumSamples)

---------------------------------------------------------------------------

Configure Trips

Bitmap: C:\Program Files\Biomek Software\Bitmaps\Vials.bmp

Description:

Caption: Configure Trips

Code: ' --- trip calculation for SN ---

nTripsSN = TLS.NumTrips(VolMaxSB, MaxVolInTips)

Extend "NumTripsSN", nTripsSN

Extend "VolTripSN",  VolMaxSB/nTripsSN


Extend "VolSN", VolMaxSB


CurrentVol = VolMaxSB+1 ' +1 takes care of a rounding error in RemoveSN

                       ' (cannot asp 866.667 because the well only has 866.667)


Set LW = Positions(Labware.Location("Cryo1")).Labware

LW.ConfigureAmounts CurrentVol




'tls.msg "SampleSN = " & ntripssn & " * " & (VolMaxSB/nTripsSN) & " µl = " & volmaxsb
```

Prompt: [IDispatch]

Tooltip: Scripted Let

Loop

Loop from "Trip" = "1" to "=NumTripsSN", incrementing by "1".

Calc Asp Height

Bitmap: C:\Program Files\Biomek Software\Bitmaps\Vials.bmp

Description:

Caption: Calc Asp Height

```
Code: ' calculate the asp height so we don't need to use LLS

' assume it's the same for all wells

' first calculate the current volume in the well

vol = VolSN - (VolTripSN * (Trip - 1))


Set LW = Positions(Labware.Location("Cryo1")).Labware

AspHeightCM = LW.HeightFromVolume(vol, 1) - SNStartBelowCM ' start below liq level

' this is the height in CM

' needs to be multiplied by 10 because the step expects MM


Extend "AspHeight",  AspHeightCM * 10 ' convert cm to mm


'tls.msg "Trip " & trip & " of " & numtripssn & vblf & "AspHeight = " & AspHeightCM * 10
```

Prompt: [IDispatch]

Tooltip: Scripted Let

Loop

Loop from "ColPair" = "1" to "3", incrementing by "1".

Set Well Pattern

Bitmap: C:\Program Files\Biomek Software\Bitmaps\Vials.bmp

Description:

Caption: Set Well Pattern

Code: Select Case ColPair

```
   Case 1

      Extend "Wells", Col()(1)(MFX.NumSamples) & "," & Col()(2)(MFX.NumSamples)

   Case 2

      Extend "Wells", Col()(3)(MFX.NumSamples) & "," & Col()(4)(MFX.NumSamples)

   Case 3

      Extend "Wells", Col()(5)(MFX.NumSamples) & "," & Col()(6)(MFX.NumSamples)

End Select
```

Prompt: [IDispatch]

Tooltip: Scripted Let

Transfer

Using Pod2, execute the following transfer:

From: Cryo1, sections specified by "=Wells", BeadsplusCells

Proceed down first, then left to right.

Start from the beginning of the selection.

Do not set the mark.

Use the following custom technique:

  Use the following pipetting template: OS Span-8 P1000 SlowRetract 2

  Calibration Offset: 2.54

  Calibration Slope: 1.033

  Minimum Pipetting Height: -0.5 mm

  Prewet: False

Aspirate Blowout: True

Follow Liquid: True

Height: -2 mm from the liquid

Mix: False

Mix Aspirate Speed: 100µL/s

Mix Aspirate Height: 0 mm from the liquid

Mix Dispense Speed: 100µL/s

Mix Dispense Height: 0 mm from the liquid

Mix Count: 1

Mix Volume: 10 µL

Operation speed: 20µL/s

Tip Touch: False

Trailing Air Gap: False

Override Liquid Type Settings

Aspirate Delay: 10 ms

Aspirate Speed: 300 µL/s

Blowout Delay: 0 ms

Blowout Volume: 5 µL

Dispense Delay: 0 ms

Dispense Speed: 50 µL/s

Prewet Delay: 0 ms

Prewet Overage: 0 µL

Tip Touch Delay: 0 ms

Tip Touch Speed: 50 µL/s

Trailing Air Gap Volume: 1 µL

Override the technique height by moving to =AspHeight mm from the bottom.

To: Cryo1, =VolTripSNµL, sections specified by "=Wells", BeadsplusCells

Proceed down first, then left to right.

Start from the beginning of the selection.

Set the mark at the last well transferred.

Use the following custom technique:

  Use the following pipetting template: Do Nothing

  Calibration Offset: 2.54

  Calibration Slope: 1.033

  Minimum Pipetting Height: 0 mm

  Prewet: False

  Blowout: True

  Follow Liquid: False

  Height: 1.5 mm from the bottom

  Mix: False

  Mix Aspirate Speed: 100µL/s

  Mix Aspirate Height: 0 mm from the liquid

  Mix Dispense Speed: 100µL/s

  Mix Dispense Height: 0 mm from the liquid

  Mix Count: 1

  Mix Volume: 10 µL

  Operation speed: 5µL/s

  Tip Touch: False

  Override Liquid Type Settings

    Aspirate Delay: 10 ms

    Aspirate Speed: 50 µL/s

    Blowout Delay: 0 ms

    Blowout Volume: 5 µL

    Dispense Delay: 0 ms

    Dispense Speed: 50 µL/s

    Prewet Delay: 0 ms

    Prewet Overage: 0 µL

    Tip Touch Delay: 0 ms

    Tip Touch Speed: 50 µL/s

    Trailing Air Gap Volume: 1 µL

Override the technique height by moving to 39.3 mm from the bottom.

Dispense up to 1 time(s) per draw.

Create 1 replicate(s) of each source well.

Keep the current tips

Keep the tips when finished.

Stop when finished with Destinations.

Probes specified by "=TwoTips()(MFX.NumSamples)" will be used.

Run emptytipstowaste

End Scripted Let

End Loop

End Scripted Let

End Loop

Script

Description:

Labware Place to Cryo1

Execute the following script code:

Option Explicit

' This script duplicates the functionality of the Move Labware step.

```
' It has been modified to only place the labware after another step has picked it up.
Dim e
Set e = CreateObject("World.EngineObject")
Dim Target, PodName
Dim Pod, CurDeck, OrigPodSpeed, LWSpeedLimit

' PodName        The name of the pod to use
PodName = StepDictionary.Let.PodName
if IsEmpty(PodName) or PodName = "" then err.raise 1050, , "PodName must be defined"
Set Pod = World.Devices.Pipettor1(PodName)
OrigPodSpeed = Pod.CurrentSpeed

Set CurDeck = World.Devices.Pipettor1.Deck

' Target         Name of the target position (string)
' Need to use Deck.FindLabwarePosition,
' in case labware is referred to by name instead of location
Target = CurDeck.FindLabwarePosition(e.EvaluateExpression(StepDictionary.Let.Target))

if IsEmpty(Target) or Target = "" then err.raise 1050, , "Target must be defined"

If Not IsObject(Pod.GrippedLabware) Then err.raise 1050, , "There is no labware in the
gripper."
Dim glw
set glw = Pod.GrippedLabware(Pod.GrippedLabware.Count - 1) ' The gripped labware is an
array with the bottom piece at the highest index

' Convert Source and Target names into objects
```

```
Dim Dst
Set Dst = Pipettor.Deck.Positions(Target)

Dim Z
Dim sx, sy, sz
' Dim gx, gy, gz, pd
'pd = CDbl(Pod.VariantDictionary.GetDefault("GripperDOffset", 0.0))

' Get the squeeze values
'Dim Unsqueeze
'Unsqueeze = CDbl(Pod.DAxisFromGripper(CDbl(pd) + CDbl(glw.Class.Unsqueeze)))

' Get the Pod gripper offsets
Dim px, py, pz
px = CDbl(Pod.GripperXOffset)
py = CDbl(Pod.GripperYOffset)
pz = CDbl(Pod.GripperZOffset)

' Enforce the labware's speed limit
'LWSpeedLimit = srcLabware.Class.SpeedLimit
'If LWSpeedLimit < OrigPodSpeed Then Pod.SetSpeed LWSpeedLimit

'Get height of destination position
Z = CDbl(Dst.Z(Pod))

' Get offsets from position to the labware at target position
Dim tx, ty, tz, dx, dy, dz
tx = CDbl(Dst.GetOneLabwareOffsetAtDepth(1, "X"))
ty = CDbl(Dst.GetOneLabwareOffsetAtDepth(1, "Y"))
```

```
tz = CDbl(Dst.GetOneLabwareOffsetAtDepth(1, "Z"))

' Get the Target Labware
Dim dstLabware
dx = 0
dy = 0
dz = 0
dstLabware = Null
if (Dst.StackDepth > 0) then
  Set dstLabware = Dst.GetLabwareAtDepth(1)
end if

' Get Destination Stack Offsets (really Per-Labware Offsets)
dx = CDbl(Dst.GetOneStackOffset(glw, dstLabware, "X"))
dy = CDbl(Dst.GetOneStackOffset(glw, dstLabware, "Y"))
dz = CDbl(Dst.GetOneStackOffset(glw, dstLabware, "Z"))

' Get the labware gripper offsets (added-in by MAG 3/7/2003)
World.Volatile.MyDest = Dst

'Dim gx, gy, gz, pd
'gx = CDbl(glw.Class.GripperXOffset)
'gy = CDbl(glw.Class.GripperYOffset)
'gz = CDbl(glw.Class.GripperZOffset)

pd = CDbl(Pod.VariantDictionary.GetDefault("GripperDOffset", 0.0))

' Get the labware gripper offsets and squeeze values
```

```
Dim gx, gy, gz, pd

Dim Unsqueeze

Select Case Pod.PodType

   Case "ATTILA"

      gx = CDbl(glw.Class.GripperInfo.MultiChannel.GripperXOffset)

      gy = CDbl(glw.Class.GripperInfo.MultiChannel.GripperYOffset)

      gz = CDbl(glw.Class.GripperInfo.MultiChannel.GripperZOffset)

      Unsqueeze = CDbl(Pod.DAxisFromGripper(CDbl(pd) +
CDbl(glw.Class.GripperInfo.MultiChannel.Unsqueeze)))

'tls.msg pod.podtype

'tls.msg gx & " " & gy & " " & gz & " "  & " " & Unsqueeze

   Case Else ' need to fill in code for NX Gripper

End Select




' Open Destination

Dst.Open True, 0


' Start Moving

Pod.ApproachPosition Target, CDbl(tx+dx+gx-px), CDbl(ty+dy+gy-py), CDbl(Z + tz+dz+gz-pz),
True


Pod.MoveZ CDbl(Z + tz + dz + gz - pz)


' Put Labware

Pod.AbsMove ,,,Unsqueeze
```

```
' Notify software the labware that was in the grippers is now at the target
' old v. 2.x syntax Pod.PutLabware Target
Pod.Gripper.PutLabwareObject Target

' Retract grippers
Pod.GripperRetract

' Close the destination position
Dst.Close True, 0

'Restore original pod speed now that the move is finished
'Pod.SetSpeed OrigPodSpeed

' Move to a safe height
Pod.MoveToSafe
---------------------------------------------------------------------------
Run droptips
---------------------------------------------------------------------------
Run cleanwashstation
  dspeed = 200
  fromtop = 0.6
  yoffset = 0.35
---------------------------------------------------------------------------
Run setstepdone
  wait = false
---------------------------------------------------------------------------
End Scripted Let
---------------------------------------------------------------------------
```

End

Else

End

If

If "MFX.NextStep = 9":

Then

Move To

Move "Pod2" to deck position "W1" at offset (0,0).

Move Labware

Move the top "2" plates at "Cryo1" to "Orbital1" using pod "Pod1".

Move To

Move "Pod1" to deck position "P2" at offset (0,0).

Run setstepdone

  wait = true

End

Else

End

End Group

Loop

Loop from "WashCycle" = "=MFX.RoundsCompleted(6)" to "=MFX.TotalRounds(6)", incrementing by "1".

If

If "MFX.NextStep = 10":

Then

Run getnewtips

  pattern = =TwoTips()(MFX.NumSamples)

Script

Description:

Labware Pickup from Cryo1

Execute the following script code:

```
Option Explicit

' This script duplicates the functionality of the first half of the Move Labware step.

' Modified 3/7/2003 by MAG to lift the labware to a specific height off the deck

' (height measured at top of the labware) which was useful for a specific customer

' reading barcodes via their own reader, with labels always a certain distance from

' the top of the labware.

Set e = CreateObject("World.EngineObject")

Dim Source, Target, PodName
```

```
Dim Pod, CurDeck, OrigPodSpeed, LWSpeedLimit

' PodName        The name of the pod to use
PodName = StepDictionary.Let.PodName
if IsEmpty(PodName) or PodName = "" then err.raise 1050, , "PodName must be defined"
Set Pod = World.Devices.Pipettor1(PodName)
OrigPodSpeed = Pod.CurrentSpeed

Set CurDeck = World.Devices.Pipettor1.Deck

' Source         Name of the source position (string)
' Need to use Deck.FindLabwarePosition,
' in case labware is referred to by name instead of location
Source = CurDeck.FindLabwarePosition(e.EvaluateExpression(StepDictionary.Let.Source))
'tls.msg MFX.NextStep & " " & source
if IsEmpty(Source) or Source = "" then err.raise 1050, , "Source must be defined"

' Get the depth to grab a piece of labware at.
Dim Depth
Depth = e.EvaluateExpression(StepDictionary.Let.Depth)
if IsEmpty(Depth)         then err.raise 1050, , "Depth must be defined"
If Depth < 1              then err.raise 1050, , "Depth must be at least 1"

' Convert Source and Target names into objects
Dim Src
Set Src = Pipettor.Deck.Positions(Source)

' Get the Source Labware
Dim srcLabware
```

```
World.Volatile.Depth = Depth
Set srcLabware = Src.GetLabwareAtDepth(Depth)

' Get the source labware height  [DON'T NEED THIS ANYMORE]
' Dim srcLwHeight
' srcLwHeight = srcLabware.Class.Height

Dim Z
Z = CDbl(Src.Z(Pod))

' Get offsets from position to the labware at source position
Dim sx, sy, sz
sx = CDbl(Src.GetOneLabwareOffsetAtDepth(Depth, "X"))
sy = CDbl(Src.GetOneLabwareOffsetAtDepth(Depth, "Y"))
sz = CDbl(Src.GetOneLabwareOffsetAtDepth(Depth, "Z"))

pd = CDbl(Pod.VariantDictionary.GetDefault("GripperDOffset", 0.0))

' Get the labware gripper offsets and squeeze values
Dim gx, gy, gz, pd
Dim Squeeze, Unsqueeze
Select Case Pod.PodType
   Case "ATTILA"
      gx = CDbl(srcLabware.Class.GripperInfo.MultiChannel.GripperXOffset)
      gy = CDbl(srcLabware.Class.GripperInfo.MultiChannel.GripperYOffset)
      gz = CDbl(srcLabware.Class.GripperInfo.MultiChannel.GripperZOffset)
      Squeeze = CDbl(Pod.DAxisFromGripper(CDbl(pd) +
CDbl(srcLabware.Class.GripperInfo.MultiChannel.Squeeze)))
```

```
      Unsqueeze = CDbl(Pod.DAxisFromGripper(CDbl(pd) +
CDbl(srcLabware.Class.GripperInfo.MultiChannel.Unsqueeze)))
'tls.msg pod.podtype
'tls.msg gx & " " & gy & " " & gz & " " & Squeeze & " " & Unsqueeze
   Case Else ' need to fill in code for NX Gripper
End Select

' Get the Pod gripper offsets
Dim px, py, pz
px = CDbl(Pod.GripperXOffset)
py = CDbl(Pod.GripperYOffset)
pz = CDbl(Pod.GripperZOffset)

Dim LiftHeight, e

LiftHeight = e.EvaluateExpression(StepDictionary.Let.LiftHeight)

' Temporarily save the height of the move
World.Volatile.LiftHeight = LiftHeight

'Reserve the resources used, i.e., the source position and the Pod
World.Globals.ResourceReserver.ReserveResources Array(Src, Pod)

' Open the source position
Src.Open True, Depth

' Start Moving
Pod.ApproachPosition Source, sx+gx-px, sy+gy-py, Z + sz+gz-pz, False, Unsqueeze
```

```
' Extend the grippers
Pod.GripperExtend

' Grab Labware
Pod.AbsMove ,,,Squeeze

' Enforce the labware's speed limit
LWSpeedLimit = srcLabware.Class.SpeedLimit
If LWSpeedLimit < OrigPodSpeed Then Pod.SetSpeed LWSpeedLimit

' Notify software that the source labware is now in the grippers
' Version 2.x syntax: Pod.GetLabware Source, Depth
Pod.Gripper.GetLabwareObject Source, Depth

' Move to a safe height above the position (original function commented out)
' Pod.MoveToSafe

If LiftHeight < 0 Then err.raise 1050, ,  "Lift Height too low ; would result in downward
motion"

Pod.RelativeMoveAxes "Z", LiftHeight

' Close the source position
' Src.Close True, Depth

'Restore original pod speed now that the move is finished
Pod.SetSpeed OrigPodSpeed
---------------------------------------------------------------------------
```

Configure Step

Bitmap: C:\Program Files\Biomek Software\Bitmaps\Vials.bmp

Description:

Caption: Configure Step

Code: Select Case WashCycle

```
   Case 0,1,2
      VolWB  = MFX.P6VolWash(WashCycle+1) ' + 1 because WashCycle is 0 in the first round,
1 in the second etc.
   Case Else
      VolWB  = MFX.P6VolWash(4)
End Select

' --- trip calculation for WB addition ---
nTripsWB = TLS.NumTrips(VolWB, MaxVolInTips)
Extend "NumTripsWB", nTripsWB
Extend "VolTripWB",  VolWB/nTripsWB

' set well volumes to empty
Positions(Labware.Location("Cryo1")).Labware.ConfigureAmounts 0
```

Prompt: [IDispatch]

Tooltip: Scripted Let

Loop

Loop from "Trip" = "1" to "=NumTripsWB", incrementing by "1".

Loop

Loop from "ColPair" = "1" to "3", incrementing by "1".

Set Well Pattern; Refill Buffer

Bitmap: C:\Program Files\Biomek Software\Bitmaps\Vials.bmp

Description:

Caption: Set Well Pattern; Refill Buffer

```
Code: Select Case ColPair

   Case 1

      Extend "Wells", Col()(1)(MFX.NumSamples) & "," & Col()(2)(MFX.NumSamples)

   Case 2

      Extend "Wells", Col()(3)(MFX.NumSamples) & "," & Col()(4)(MFX.NumSamples)

   Case 3

      Extend "Wells", Col()(5)(MFX.NumSamples) & "," & Col()(6)(MFX.NumSamples)

End Select


Set LW = Positions(Pipettor.Deck.FindLabwarePosition("Buffer")).Labware

LW.ConfigureAmounts 20000
```

Prompt: [IDispatch]

Tooltip: Scripted Let

Transfer

Using Pod2, execute the following transfer:

From: Buffer, sections specified by "=OneTip()(MFX.NumSamples)", Water

Proceed down first, then left to right.

Start from the beginning of the selection.

Set the mark at the last well transferred.

Use the following custom technique:

  Use the following pipetting template: OS Span-8 P1000 SlowRetract 2

  Calibration Offset: 2.54

  Calibration Slope: 1.033

  Minimum Pipetting Height: 1.5 mm

Prewet: False

Aspirate Blowout: False

Follow Liquid: True

Height: -2 mm from the liquid

Mix: False

Mix Aspirate Speed: 100µL/s

Mix Aspirate Height: 0 mm from the liquid

Mix Dispense Speed: 100µL/s

Mix Dispense Height: 0 mm from the liquid

Mix Count: 1

Mix Volume: 10 µL

Operation speed: 70µL/s

Tip Touch: False

Trailing Air Gap: False

Override Liquid Type Settings

Aspirate Delay: 10 ms

Aspirate Speed: 300 µL/s

Blowout Delay: 0 ms

Blowout Volume: 5 µL

Dispense Delay: 0 ms

Dispense Speed: 50 µL/s

Prewet Delay: 0 ms

Prewet Overage: 0 µL

Tip Touch Delay: 0 ms

Tip Touch Speed: 50 µL/s

Trailing Air Gap Volume: 1 µL

Override the technique height by moving to 1 mm from the bottom.

To: Cryo1, =VolTripWBµL, sections specified by "=Wells", Water

Proceed down first, then left to right.

Start from the beginning of the selection.

Set the mark at the last well transferred.

Use the following custom technique:

Use the following pipetting template: Span-8 P1000 2

Calibration Offset: 0

Calibration Slope: 1

Minimum Pipetting Height: 1.5 mm

Prewet: False

Blowout: True

Follow Liquid: False

Height: 1.5 mm from the bottom

Mix: False

Mix Aspirate Speed: 100µL/s

Mix Aspirate Height: 0 mm from the liquid

Mix Dispense Speed: 100µL/s

Mix Dispense Height: 0 mm from the liquid

Mix Count: 1

Mix Volume: 10 µL

Operation speed: 70µL/s

Tip Touch: False

Override Liquid Type Settings

Aspirate Delay: 10 ms

Aspirate Speed: 300 µL/s

Blowout Delay: 0 ms

Blowout Volume: 5 µL

Dispense Delay: 0 ms

Dispense Speed: 300 µL/s

Prewet Delay: 0 ms

Prewet Overage: 0 µL

Tip Touch Delay: 0 ms

Tip Touch Speed: 50 µL/s

Trailing Air Gap Volume: 1 µL

Override the technique height by moving to -5 mm from the top.

Dispense up to 1 time(s) per draw.

Create 1 replicate(s) of each source well.

Keep the current tips

Keep the tips when finished.

Stop when finished with Destinations.

Probes specified by "=TwoTips()(MFX.NumSamples)" will be used.

End Scripted Let

End Loop

End Loop

End Scripted Let

Script

Description:

Labware Place to Cryo1

Execute the following script code:

Option Explicit

' This script duplicates the functionality of the Move Labware step.

' It has been modified to only place the labware after another step has picked it up.

```
Dim e
Set e = CreateObject("World.EngineObject")
Dim Target, PodName
Dim Pod, CurDeck, OrigPodSpeed, LWSpeedLimit

' PodName        The name of the pod to use
PodName = StepDictionary.Let.PodName
if IsEmpty(PodName) or PodName = "" then err.raise 1050, , "PodName must be defined"
Set Pod = World.Devices.Pipettor1(PodName)
OrigPodSpeed = Pod.CurrentSpeed

Set CurDeck = World.Devices.Pipettor1.Deck

' Target         Name of the target position (string)
' Need to use Deck.FindLabwarePosition,
' in case labware is referred to by name instead of location
Target = CurDeck.FindLabwarePosition(e.EvaluateExpression(StepDictionary.Let.Target))

if IsEmpty(Target) or Target = "" then err.raise 1050, , "Target must be defined"

If Not IsObject(Pod.GrippedLabware) Then err.raise 1050, , "There is no labware in the
gripper."
Dim glw
set glw = Pod.GrippedLabware(Pod.GrippedLabware.Count - 1) ' The gripped labware is an
array with the bottom piece at the highest index

' Convert Source and Target names into objects
Dim Dst
```

```
Set Dst = Pipettor.Deck.Positions(Target)

Dim Z
Dim sx, sy, sz
' Dim gx, gy, gz, pd
'pd = CDbl(Pod.VariantDictionary.GetDefault("GripperDOffset", 0.0))

' Get the squeeze values
'Dim Unsqueeze
'Unsqueeze = CDbl(Pod.DAxisFromGripper(CDbl(pd) + CDbl(glw.Class.Unsqueeze)))

' Get the Pod gripper offsets
Dim px, py, pz
px = CDbl(Pod.GripperXOffset)
py = CDbl(Pod.GripperYOffset)
pz = CDbl(Pod.GripperZOffset)

' Enforce the labware's speed limit
'LWSpeedLimit = srcLabware.Class.SpeedLimit
'If LWSpeedLimit < OrigPodSpeed Then Pod.SetSpeed LWSpeedLimit

'Get height of destination position
Z = CDbl(Dst.Z(Pod))

' Get offsets from position to the labware at target position
Dim tx, ty, tz, dx, dy, dz
tx = CDbl(Dst.GetOneLabwareOffsetAtDepth(1, "X"))
ty = CDbl(Dst.GetOneLabwareOffsetAtDepth(1, "Y"))
tz = CDbl(Dst.GetOneLabwareOffsetAtDepth(1, "Z"))
```

```
' Get the Target Labware
Dim dstLabware
dx = 0
dy = 0
dz = 0
dstLabware = Null
if (Dst.StackDepth > 0) then
  Set dstLabware = Dst.GetLabwareAtDepth(1)
end if

' Get Destination Stack Offsets (really Per-Labware Offsets)
dx = CDbl(Dst.GetOneStackOffset(glw, dstLabware, "X"))
dy = CDbl(Dst.GetOneStackOffset(glw, dstLabware, "Y"))
dz = CDbl(Dst.GetOneStackOffset(glw, dstLabware, "Z"))

' Get the labware gripper offsets (added-in by MAG 3/7/2003)
World.Volatile.MyDest = Dst

'Dim gx, gy, gz, pd
'gx = CDbl(glw.Class.GripperXOffset)
'gy = CDbl(glw.Class.GripperYOffset)
'gz = CDbl(glw.Class.GripperZOffset)

pd = CDbl(Pod.VariantDictionary.GetDefault("GripperDOffset", 0.0))

' Get the labware gripper offsets and squeeze values
Dim gx, gy, gz, pd
```

```
Dim Unsqueeze

Select Case Pod.PodType

   Case "ATTILA"

      gx = CDbl(glw.Class.GripperInfo.MultiChannel.GripperXOffset)

      gy = CDbl(glw.Class.GripperInfo.MultiChannel.GripperYOffset)

      gz = CDbl(glw.Class.GripperInfo.MultiChannel.GripperZOffset)

      Unsqueeze = CDbl(Pod.DAxisFromGripper(CDbl(pd) +
CDbl(glw.Class.GripperInfo.MultiChannel.Unsqueeze)))

'tls.msg pod.podtype

'tls.msg gx & " " & gy & " " & gz & " "  & " " & Unsqueeze

   Case Else ' need to fill in code for NX Gripper

End Select




' Open Destination

Dst.Open True, 0


' Start Moving

Pod.ApproachPosition Target, CDbl(tx+dx+gx-px), CDbl(ty+dy+gy-py), CDbl(Z + tz+dz+gz-pz),
True


Pod.MoveZ CDbl(Z + tz + dz + gz - pz)


' Put Labware

Pod.AbsMove ,,,Unsqueeze


' Notify software the labware that was in the grippers is now at the target
```

```
' old v. 2.x syntax Pod.PutLabware Target

Pod.Gripper.PutLabwareObject Target


' Retract grippers

Pod.GripperRetract


' Close the destination position

Dst.Close True, 0


'Restore original pod speed now that the move is finished

'Pod.SetSpeed OrigPodSpeed


' Move to a safe height

Pod.MoveToSafe
```

Run parkinwashstation

  withtipwash = False

Run setstepdone

  wait = False

End

Else

End

If

If "MFX.NextStep = 11":

Then

Configure Shake Time

Bitmap: C:\Program Files\Biomek Software\Bitmaps\Vials.bmp

Description:

Caption: Configure Shake Time

```
Code: Select Case WashCycle
   Case 0,1,2
      Extend "P6ShakeTime", MFX.P6ShakeTime(WashCycle+1)
   Case Else
      Extend "P6ShakeTime", MFX.P6ShakeTime(4)
End Select
```

Prompt: [IDispatch]

Tooltip: Scripted Let

Device Action

Send the following command to "OrbitalShakerALP0": "Shake".

Parameters: =MFX.P6ShakerSpeed, 1, CounterClockwise

Run waittimer

```
  index = =""
  settimer = True
  timername = = "Shaking..."
  waittime = = P6ShakeTime
```

Device Action

Send the following command to "OrbitalShakerALP0": "Stop".

Parameters: 1

End Scripted Let

Run setstepdone

  wait = False

End

Else

End

If

If "MFX.NextStep = 12":

Then

Script

Description:

Labware Pickup from Cryo1

Execute the following script code:

```
Option Explicit

' This script duplicates the functionality of the first half of the Move Labware step.

' Modified 3/7/2003 by MAG to lift the labware to a specific height off the deck

' (height measured at top of the labware) which was useful for a specific customer

' reading barcodes via their own reader, with labels always a certain distance from

' the top of the labware.
```

```
Set e = CreateObject("World.EngineObject")

Dim Source, Target, PodName
Dim Pod, CurDeck, OrigPodSpeed, LWSpeedLimit

' PodName        The name of the pod to use
PodName = StepDictionary.Let.PodName
if IsEmpty(PodName) or PodName = "" then err.raise 1050, , "PodName must be defined"
Set Pod = World.Devices.Pipettor1(PodName)
OrigPodSpeed = Pod.CurrentSpeed

Set CurDeck = World.Devices.Pipettor1.Deck

' Source         Name of the source position (string)
' Need to use Deck.FindLabwarePosition,
' in case labware is referred to by name instead of location
Source = CurDeck.FindLabwarePosition(e.EvaluateExpression(StepDictionary.Let.Source))

if IsEmpty(Source) or Source = "" then err.raise 1050, , "Source must be defined"

' Get the depth to grab a piece of labware at.
Dim Depth
Depth = e.EvaluateExpression(StepDictionary.Let.Depth)
if IsEmpty(Depth)         then err.raise 1050, , "Depth must be defined"
If Depth < 1              then err.raise 1050, , "Depth must be at least 1"

' Convert Source and Target names into objects
Dim Src
```

```
Set Src = Pipettor.Deck.Positions(Source)

' Get the Source Labware
Dim srcLabware
World.Volatile.Depth = Depth
Set srcLabware = Src.GetLabwareAtDepth(Depth)

' Get the source labware height  [DON'T NEED THIS ANYMORE]
' Dim srcLwHeight
' srcLwHeight = srcLabware.Class.Height

Dim Z
Z = CDbl(Src.Z(Pod))

' Get offsets from position to the labware at source position
Dim sx, sy, sz
sx = CDbl(Src.GetOneLabwareOffsetAtDepth(Depth, "X"))
sy = CDbl(Src.GetOneLabwareOffsetAtDepth(Depth, "Y"))
sz = CDbl(Src.GetOneLabwareOffsetAtDepth(Depth, "Z"))

pd = CDbl(Pod.VariantDictionary.GetDefault("GripperDOffset", 0.0))

' Get the labware gripper offsets and squeeze values
Dim gx, gy, gz, pd
Dim Squeeze, Unsqueeze
Select Case Pod.PodType
   Case "ATTILA"
      gx = CDbl(srcLabware.Class.GripperInfo.MultiChannel.GripperXOffset)
```

```
      gy = CDbl(srcLabware.Class.GripperInfo.MultiChannel.GripperYOffset)
      gz = CDbl(srcLabware.Class.GripperInfo.MultiChannel.GripperZOffset)
      Squeeze = CDbl(Pod.DAxisFromGripper(CDbl(pd) +
CDbl(srcLabware.Class.GripperInfo.MultiChannel.Squeeze)))
      Unsqueeze = CDbl(Pod.DAxisFromGripper(CDbl(pd) +
CDbl(srcLabware.Class.GripperInfo.MultiChannel.Unsqueeze)))
'tls.msg pod.podtype
'tls.msg gx & " " & gy & " " & gz & " " & Squeeze & " " & Unsqueeze
   Case Else ' need to fill in code for NX Gripper
End Select

' Get the Pod gripper offsets
Dim px, py, pz
px = CDbl(Pod.GripperXOffset)
py = CDbl(Pod.GripperYOffset)
pz = CDbl(Pod.GripperZOffset)

Dim LiftHeight, e

LiftHeight = e.EvaluateExpression(StepDictionary.Let.LiftHeight)

' Temporarily save the height of the move
World.Volatile.LiftHeight = LiftHeight

'Reserve the resources used, i.e., the source position and the Pod
World.Globals.ResourceReserver.ReserveResources Array(Src, Pod)

' Open the source position
Src.Open True, Depth
```

```
' Start Moving
Pod.ApproachPosition Source, sx+gx-px, sy+gy-py, Z + sz+gz-pz, False, Unsqueeze

' Extend the grippers
Pod.GripperExtend

' Grab Labware
Pod.AbsMove ,,,Squeeze

' Enforce the labware's speed limit
LWSpeedLimit = srcLabware.Class.SpeedLimit
If LWSpeedLimit < OrigPodSpeed Then Pod.SetSpeed LWSpeedLimit

' Notify software that the source labware is now in the grippers
' Version 2.x syntax: Pod.GetLabware Source, Depth
Pod.Gripper.GetLabwareObject Source, Depth

' Move to a safe height above the position (original function commented out)
' Pod.MoveToSafe

If LiftHeight < 0 Then err.raise 1050, ,  "Lift Height too low ; would result in downward
motion"

Pod.RelativeMoveAxes "Z", LiftHeight

' Close the source position
' Src.Close True, Depth
```

```
'Restore original pod speed now that the move is finished

Pod.SetSpeed OrigPodSpeed

---------------------------------------------------------------------------

Run getnewtips

  pattern = =TwoTips()(MFX.NumSamples)

---------------------------------------------------------------------------

Loop

Loop from "ColPair" = "1" to "3", incrementing by "1".

---------------------------------------------------------------------------

Set Well Pattern

Bitmap: C:\Program Files\Biomek Software\Bitmaps\Vials.bmp

Description:

Caption: Set Well Pattern

Code: Select Case ColPair

   Case 1

      Extend "Wells", Col()(1)(MFX.NumSamples) & "," & Col()(2)(MFX.NumSamples)

   Case 2

      Extend "Wells", Col()(3)(MFX.NumSamples) & "," & Col()(4)(MFX.NumSamples)

   Case 3

      Extend "Wells", Col()(5)(MFX.NumSamples) & "," & Col()(6)(MFX.NumSamples)

End Select


Extend "Delay", 0 '


' New in 0.15: Start aspirating at 0.5 * liquid height, follow liquid

' down to 2 mm above bottom. Dispense at 2 mm above bottom and follow the liquid up.


' determine the liquid height
```

```
Select Case WashCycle
   Case 0,1,2
      vol = MFX.P6VolWash(WashCycle+1)
   Case Else
      vol = MFX.P6VolWash(4)
End Select

Extend "VolTipMix", TLS.LesserOf(MaxVolInTips, vol)

Set LW = Positions(Labware.Location("Cryo1")).Labware
zliqCM = 0.5 * LW.HeightFromVolume(vol, 1)
' this is the height in CM
' needs to be multiplied by 10 because the step expects MM
' also, ZOffset needs to be added

Extend "ZLiqHalf",  ZOffset + (zliqCM * 10) ' convert cm to mm

Prompt: [IDispatch]
Tooltip: Scripted Let
---------------------------------------------------------------------------
Transfer
Using Pod2, execute the following transfer:
From: Cryo1, sections specified by "=Wells", Water
Proceed down first, then left to right.
Start from the beginning of the selection.
Do not set the mark.
Use the following custom technique:
  Use the following pipetting template: OS Span-8 P1000 MixOnly 2
```

Calibration Offset: 2.54

Calibration Slope: 1.033

Minimum Pipetting Height: 2 mm

Prewet: False

Aspirate Blowout: True

Follow Liquid: True

Height: 0 mm from the liquid

Mix: True

Mix Aspirate Speed: =MFX.P6MixSpeedµL/s

Mix Aspirate Height: =ZLiqHalf mm from the liquid

Mix Dispense Speed: =MFX.P6MixSpeedµL/s

Mix Dispense Height: 2 mm from the liquid

Mix Count: =MFX.P6MixCycles

Mix Volume: =VolTipMix µL

Operation speed: 20µL/s

Tip Touch: False

Trailing Air Gap: False

Override Liquid Type Settings

  Aspirate Delay: 10 ms

  Aspirate Speed: 300 µL/s

  Blowout Delay: =Delay ms

  Blowout Volume: 20 µL

  Dispense Delay: 20 ms

  Dispense Speed: 200 µL/s

  Prewet Delay: 200 ms

  Prewet Overage: 0 µL

  Tip Touch Delay: 300 ms

  Tip Touch Speed: 70 µL/s

  Trailing Air Gap Volume: 10 µL

Override the technique height by moving to 5 mm from the bottom.

To: Cryo1, 0µL, sections specified by "=Wells", BeadsplusCells

Proceed down first, then left to right.

Start from the beginning of the selection.

Do not set the mark.

Use the following custom technique:

Use the following pipetting template: OS Span-8 P1000 MixOnly 2

Calibration Offset: 2.54

Calibration Slope: 1.033

Minimum Pipetting Height: 0.5 mm

Prewet: False

Blowout: False

Follow Liquid: False

Height: 0 mm from the liquid

Mix: False

Mix Aspirate Speed: 100µL/s

Mix Aspirate Height: 0 mm from the liquid

Mix Dispense Speed: 100µL/s

Mix Dispense Height: 0 mm from the liquid

Mix Count: 1

Mix Volume: 900 µL

Operation speed: 70µL/s

Tip Touch: False

Override Liquid Type Settings

Aspirate Delay: 10 ms

Aspirate Speed: 300 µL/s

Blowout Delay: 10 ms

Blowout Volume: 20 µL

Dispense Delay: 20 ms

Dispense Speed: 200 µL/s

Prewet Delay: 0 ms

Prewet Overage: 0 µL

Tip Touch Delay: 300 ms

Tip Touch Speed: 70 µL/s

Trailing Air Gap Volume: 10 µL

Override the technique height by moving to 2 mm from the bottom.

Dispense up to 1 time(s) per draw.

Create 1 replicate(s) of each source well.

Keep the current tips

Keep the tips when finished.

Stop when finished with Destinations.

Probes specified by "=OneTip()(MFX.NumSamples)" will be used.

End Scripted Let

End Loop

Script

Description:

Labware Place to Cryo1

Execute the following script code:

Option Explicit

' This script duplicates the functionality of the Move Labware step.

' It has been modified to only place the labware after another step has picked it up.

Dim e

```
Set e = CreateObject("World.EngineObject")
Dim Target, PodName
Dim Pod, CurDeck, OrigPodSpeed, LWSpeedLimit

' PodName        The name of the pod to use
PodName = StepDictionary.Let.PodName
if IsEmpty(PodName) or PodName = "" then err.raise 1050, , "PodName must be defined"
Set Pod = World.Devices.Pipettor1(PodName)
OrigPodSpeed = Pod.CurrentSpeed

Set CurDeck = World.Devices.Pipettor1.Deck

' Target         Name of the target position (string)
' Need to use Deck.FindLabwarePosition,
' in case labware is referred to by name instead of location
Target = CurDeck.FindLabwarePosition(e.EvaluateExpression(StepDictionary.Let.Target))

if IsEmpty(Target) or Target = "" then err.raise 1050, , "Target must be defined"

If Not IsObject(Pod.GrippedLabware) Then err.raise 1050, , "There is no labware in the
gripper."
Dim glw
set glw = Pod.GrippedLabware(Pod.GrippedLabware.Count - 1) ' The gripped labware is an
array with the bottom piece at the highest index

' Convert Source and Target names into objects
Dim Dst
Set Dst = Pipettor.Deck.Positions(Target)
```

```
Dim Z

Dim sx, sy, sz

' Dim gx, gy, gz, pd

'pd = CDbl(Pod.VariantDictionary.GetDefault("GripperDOffset", 0.0))


' Get the squeeze values

'Dim Unsqueeze

'Unsqueeze = CDbl(Pod.DAxisFromGripper(CDbl(pd) + CDbl(glw.Class.Unsqueeze)))


' Get the Pod gripper offsets

Dim px, py, pz

px = CDbl(Pod.GripperXOffset)

py = CDbl(Pod.GripperYOffset)

pz = CDbl(Pod.GripperZOffset)


' Enforce the labware's speed limit

'LWSpeedLimit = srcLabware.Class.SpeedLimit

'If LWSpeedLimit < OrigPodSpeed Then Pod.SetSpeed LWSpeedLimit


'Get height of destination position

Z = CDbl(Dst.Z(Pod))


' Get offsets from position to the labware at target position

Dim tx, ty, tz, dx, dy, dz

tx = CDbl(Dst.GetOneLabwareOffsetAtDepth(1, "X"))

ty = CDbl(Dst.GetOneLabwareOffsetAtDepth(1, "Y"))

tz = CDbl(Dst.GetOneLabwareOffsetAtDepth(1, "Z"))
```

```
' Get the Target Labware
Dim dstLabware
dx = 0
dy = 0
dz = 0
dstLabware = Null
if (Dst.StackDepth > 0) then
  Set dstLabware = Dst.GetLabwareAtDepth(1)
end if

' Get Destination Stack Offsets (really Per-Labware Offsets)
dx = CDbl(Dst.GetOneStackOffset(glw, dstLabware, "X"))
dy = CDbl(Dst.GetOneStackOffset(glw, dstLabware, "Y"))
dz = CDbl(Dst.GetOneStackOffset(glw, dstLabware, "Z"))

' Get the labware gripper offsets (added-in by MAG 3/7/2003)
World.Volatile.MyDest = Dst

'Dim gx, gy, gz, pd
'gx = CDbl(glw.Class.GripperXOffset)
'gy = CDbl(glw.Class.GripperYOffset)
'gz = CDbl(glw.Class.GripperZOffset)

pd = CDbl(Pod.VariantDictionary.GetDefault("GripperDOffset", 0.0))

' Get the labware gripper offsets and squeeze values
Dim gx, gy, gz, pd
Dim Unsqueeze
```

```
Select Case Pod.PodType
   Case "ATTILA"
      gx = CDbl(glw.Class.GripperInfo.MultiChannel.GripperXOffset)
      gy = CDbl(glw.Class.GripperInfo.MultiChannel.GripperYOffset)
      gz = CDbl(glw.Class.GripperInfo.MultiChannel.GripperZOffset)
      Unsqueeze = CDbl(Pod.DAxisFromGripper(CDbl(pd) +
CDbl(glw.Class.GripperInfo.MultiChannel.Unsqueeze)))
'tls.msg pod.podtype
'tls.msg gx & " " & gy & " " & gz & " "  & " " & Unsqueeze
   Case Else ' need to fill in code for NX Gripper
End Select




' Open Destination
Dst.Open True, 0

' Start Moving
Pod.ApproachPosition Target, CDbl(tx+dx+gx-px), CDbl(ty+dy+gy-py), CDbl(Z + tz+dz+gz-pz),
True

Pod.MoveZ CDbl(Z + tz + dz + gz - pz)

' Put Labware
Pod.AbsMove ,,,Unsqueeze

' Notify software the labware that was in the grippers is now at the target
' old v. 2.x syntax Pod.PutLabware Target
```

```
Pod.Gripper.PutLabwareObject Target

' Retract grippers
Pod.GripperRetract

' Close the destination position
Dst.Close True, 0

'Restore original pod speed now that the move is finished
'Pod.SetSpeed OrigPodSpeed

' Move to a safe height
Pod.MoveToSafe
---------------------------------------------------------------------------
Run parkinwashstation
  withtipwash = False
---------------------------------------------------------------------------
Run setstepdone
  wait = False
---------------------------------------------------------------------------
End
---------------------------------------------------------------------------
Else
---------------------------------------------------------------------------
End
---------------------------------------------------------------------------
If
If "MFX.NextStep = 13":
```

```
--------------------------------------------------------------------------
Then
--------------------------------------------------------------------------
Move To
Move "Pod2" to deck position "W1" at offset (0,0).
--------------------------------------------------------------------------
Move Labware
Move the top "2" plates at "Cryo1" to "Pelt1" using pod "Pod1".
--------------------------------------------------------------------------
Move To
Move "Pod1" to deck position "P2" at offset (0,0).
--------------------------------------------------------------------------
Run parkinwashstation
  rule = 3
  withtipwash = False
--------------------------------------------------------------------------
Run setstepdone
  wait = true
--------------------------------------------------------------------------
End
--------------------------------------------------------------------------
Else
--------------------------------------------------------------------------
End
--------------------------------------------------------------------------
If
If "MFX.NextStep = 14":
--------------------------------------------------------------------------
Then
```

Run waittimer

  index = =""

  settimer = True

  timername = ="Settling..."

  waittime = =SettleTime

Run setstepdone

  wait = true

End

Else

End

If

If "MFX.NextStep = 15":

Then

Script

Description:

Labware Pickup from Cryo1

Execute the following script code:

Option Explicit

' This script duplicates the functionality of the first half of the Move Labware step.

' Modified 3/7/2003 by MAG to lift the labware to a specific height off the deck

```
' (height measured at top of the labware) which was useful for a specific customer
' reading barcodes via their own reader, with labels always a certain distance from
' the top of the labware.

Set e = CreateObject("World.EngineObject")

Dim Source, Target, PodName
Dim Pod, CurDeck, OrigPodSpeed, LWSpeedLimit

' PodName        The name of the pod to use
PodName = StepDictionary.Let.PodName
if IsEmpty(PodName) or PodName = "" then err.raise 1050, , "PodName must be defined"
Set Pod = World.Devices.Pipettor1(PodName)
OrigPodSpeed = Pod.CurrentSpeed

Set CurDeck = World.Devices.Pipettor1.Deck

' Source         Name of the source position (string)
' Need to use Deck.FindLabwarePosition,
' in case labware is referred to by name instead of location
Source = CurDeck.FindLabwarePosition(e.EvaluateExpression(StepDictionary.Let.Source))

if IsEmpty(Source) or Source = "" then err.raise 1050, , "Source must be defined"

' Get the depth to grab a piece of labware at.
Dim Depth
Depth = e.EvaluateExpression(StepDictionary.Let.Depth)
if IsEmpty(Depth)         then err.raise 1050, , "Depth must be defined"
If Depth < 1              then err.raise 1050, , "Depth must be at least 1"
```

```
' Convert Source and Target names into objects
Dim Src
Set Src = Pipettor.Deck.Positions(Source)

' Get the Source Labware
Dim srcLabware
World.Volatile.Depth = Depth
Set srcLabware = Src.GetLabwareAtDepth(Depth)

' Get the source labware height  [DON'T NEED THIS ANYMORE]
' Dim srcLwHeight
' srcLwHeight = srcLabware.Class.Height

Dim Z
Z = CDbl(Src.Z(Pod))

' Get offsets from position to the labware at source position
Dim sx, sy, sz
sx = CDbl(Src.GetOneLabwareOffsetAtDepth(Depth, "X"))
sy = CDbl(Src.GetOneLabwareOffsetAtDepth(Depth, "Y"))
sz = CDbl(Src.GetOneLabwareOffsetAtDepth(Depth, "Z"))

pd = CDbl(Pod.VariantDictionary.GetDefault("GripperDOffset", 0.0))

' Get the labware gripper offsets and squeeze values
Dim gx, gy, gz, pd
Dim Squeeze, Unsqueeze
```

```
Select Case Pod.PodType
   Case "ATTILA"
      gx = CDbl(srcLabware.Class.GripperInfo.MultiChannel.GripperXOffset)
      gy = CDbl(srcLabware.Class.GripperInfo.MultiChannel.GripperYOffset)
      gz = CDbl(srcLabware.Class.GripperInfo.MultiChannel.GripperZOffset)
      Squeeze = CDbl(Pod.DAxisFromGripper(CDbl(pd) +
CDbl(srcLabware.Class.GripperInfo.MultiChannel.Squeeze)))
      Unsqueeze = CDbl(Pod.DAxisFromGripper(CDbl(pd) +
CDbl(srcLabware.Class.GripperInfo.MultiChannel.Unsqueeze)))
'tls.msg pod.podtype
'tls.msg gx & " " & gy & " " & gz & " " & Squeeze & " " & Unsqueeze
   Case Else ' need to fill in code for NX Gripper
End Select

' Get the Pod gripper offsets
Dim px, py, pz
px = CDbl(Pod.GripperXOffset)
py = CDbl(Pod.GripperYOffset)
pz = CDbl(Pod.GripperZOffset)

Dim LiftHeight, e

LiftHeight = e.EvaluateExpression(StepDictionary.Let.LiftHeight)

' Temporarily save the height of the move
World.Volatile.LiftHeight = LiftHeight

'Reserve the resources used, i.e., the source position and the Pod
World.Globals.ResourceReserver.ReserveResources Array(Src, Pod)
```

```
' Open the source position
Src.Open True, Depth

' Start Moving
Pod.ApproachPosition Source, sx+gx-px, sy+gy-py, Z + sz+gz-pz, False, Unsqueeze

' Extend the grippers
Pod.GripperExtend

' Grab Labware
Pod.AbsMove ,,,Squeeze

' Enforce the labware's speed limit
LWSpeedLimit = srcLabware.Class.SpeedLimit
If LWSpeedLimit < OrigPodSpeed Then Pod.SetSpeed LWSpeedLimit

' Notify software that the source labware is now in the grippers
' Version 2.x syntax: Pod.GetLabware Source, Depth
Pod.Gripper.GetLabwareObject Source, Depth

' Move to a safe height above the position (original function commented out)
' Pod.MoveToSafe

If LiftHeight < 0 Then err.raise 1050, ,  "Lift Height too low ; would result in downward
motion"

Pod.RelativeMoveAxes "Z", LiftHeight
```

```
' Close the source position
' Src.Close True, Depth

'Restore original pod speed now that the move is finished
Pod.SetSpeed OrigPodSpeed
---------------------------------------------------------------------------
Run getnewtips
  pattern = =TwoTips()(MFX.NumSamples)
---------------------------------------------------------------------------
Configure  Trips
Bitmap: C:\Program Files\Biomek Software\Bitmaps\Vials.bmp
Description:
Caption: Configure  Trips
Code: Select Case WashCycle
   Case 0,1,2
      volsup = MFX.P6VolWash(WashCycle+1) * 1.05
   Case Else
      volsup = MFX.P6VolWash(4) * 1.05
End Select

' --- trip calculation for SN (WB) ---
nTripsSN = TLS.NumTrips(volsup, MaxVolInTips)
Extend "NumTripsSN", nTripsSN
Extend "VolTripSN",  volsup/nTripsSN

Extend "VolSN", volsup
```

Prompt: [IDispatch]

_Commented: 0

Tooltip: Scripted Let

Loop

Loop from "Trip" = "1" to "=NumTripsSN", incrementing by "1".

Calc Asp Height

Bitmap: C:\Program Files\Biomek Software\Bitmaps\Vials.bmp

Description:

Caption: Calc Asp Height

```
Code: ' calculate the asp height so we don't need to use LLS
' assume it's the same for all wells
' first calculate the current volume in the well
vol = VolSN - (VolTripSN * (Trip - 1))

Set LW = Positions(Labware.Location("Cryo1")).Labware
AspHeightCM = LW.HeightFromVolume(vol, 1) - SNStartBelowCM ' start below liq level
' this is the height in CM
' needs to be multiplied by 10 because the step expects MM

Extend "AspHeight",  AspHeightCM * 10 ' convert cm to mm
```

```
'tls.msg "Trip " & trip & " of " & numtripssn & vblf & "AspHeight = " & AspHeightCM * 10

Positions(Labware.Location("Cryo1")).Labware.ConfigureAmounts vol+1
                                                                 ' +1 takes care of a
rounding error
                                                                 ' (cannot asp 866.667
because the well only has 866.667)
```

Prompt: [IDispatch]

Tooltip: Scripted Let

Loop

Loop from "ColPair" = "1" to "3", incrementing by "1".

Set Well Pattern

Bitmap: C:\Program Files\Biomek Software\Bitmaps\Vials.bmp

Description:

Caption: Set Well Pattern

```
Code: Select Case ColPair

   Case 1

      Extend "Wells", Col()(1)(MFX.NumSamples) & "," & Col()(2)(MFX.NumSamples)

   Case 2

      Extend "Wells", Col()(3)(MFX.NumSamples) & "," & Col()(4)(MFX.NumSamples)

   Case 3

      Extend "Wells", Col()(5)(MFX.NumSamples) & "," & Col()(6)(MFX.NumSamples)

End Select
```

Prompt: [IDispatch]

Tooltip: Scripted Let

Transfer

Using Pod2, execute the following transfer:

From: Cryo1, sections specified by "=Wells", BeadsplusCells

Proceed down first, then left to right.

Start from the beginning of the selection.

Do not set the mark.

Use the following custom technique:

Use the following pipetting template: OS Span-8 P1000 SlowRetract 2

Calibration Offset: 2.54

Calibration Slope: 1.033

Minimum Pipetting Height: -0.5 mm

Prewet: False

Aspirate Blowout: True

Follow Liquid: True

Height: -2 mm from the liquid

Mix: False

Mix Aspirate Speed: 100µL/s

Mix Aspirate Height: 0 mm from the liquid

Mix Dispense Speed: 100µL/s

Mix Dispense Height: 0 mm from the liquid

Mix Count: 1

Mix Volume: 10 µL

Operation speed: 20µL/s

Tip Touch: False

Trailing Air Gap: False

Override Liquid Type Settings

Aspirate Delay: 10 ms

Aspirate Speed: 300 µL/s

Blowout Delay: 0 ms

Blowout Volume: 5 µL

Dispense Delay: 0 ms

Dispense Speed: 50 µL/s

Prewet Delay: 0 ms

Prewet Overage: 0 µL

Tip Touch Delay: 0 ms

Tip Touch Speed: 50 µL/s

Trailing Air Gap Volume: 1 µL

Override the technique height by moving to =AspHeight mm from the bottom.

To: Cryo1, =VolTripSNµL, sections specified by "=Wells", BeadsplusCells

Proceed down first, then left to right.

Start from the beginning of the selection.

Set the mark at the last well transferred.

Use the following custom technique:

Use the following pipetting template: Do Nothing

Calibration Offset: 2.54

Calibration Slope: 1.033

Minimum Pipetting Height: 0 mm

Prewet: False

Blowout: True

Follow Liquid: False

Height: 1.5 mm from the bottom

Mix: False

Mix Aspirate Speed: 100µL/s

Mix Aspirate Height: 0 mm from the liquid

Mix Dispense Speed: 100µL/s

Mix Dispense Height: 0 mm from the liquid

Mix Count: 1

Mix Volume: 10 µL

Operation speed: 5µL/s

Tip Touch: False

Override Liquid Type Settings

Aspirate Delay: 10 ms

Aspirate Speed: 50 µL/s

Blowout Delay: 0 ms

Blowout Volume: 5 µL

Dispense Delay: 0 ms

Dispense Speed: 50 µL/s

Prewet Delay: 0 ms

Prewet Overage: 0 µL

Tip Touch Delay: 0 ms

Tip Touch Speed: 50 µL/s

Trailing Air Gap Volume: 1 µL

Override the technique height by moving to 39.3 mm from the bottom.

Dispense up to 1 time(s) per draw.

Create 1 replicate(s) of each source well.

Keep the current tips

Keep the tips when finished.

Stop when finished with Destinations.

Probes specified by "=TwoTips()(MFX.NumSamples)" will be used.

Run emptytipstowaste

End Scripted Let

End Loop

End Scripted Let

End Loop

End Scripted Let

Script

Description:

Labware Place to Cryo1

Execute the following script code:

```
Option Explicit

' This script duplicates the functionality of the Move Labware step.

' It has been modified to only place the labware after another step has picked it up.

Dim e

Set e = CreateObject("World.EngineObject")

Dim Target, PodName

Dim Pod, CurDeck, OrigPodSpeed, LWSpeedLimit


' PodName        The name of the pod to use

PodName = StepDictionary.Let.PodName

if IsEmpty(PodName) or PodName = "" then err.raise 1050, , "PodName must be defined"

Set Pod = World.Devices.Pipettor1(PodName)

OrigPodSpeed = Pod.CurrentSpeed


Set CurDeck = World.Devices.Pipettor1.Deck


' Target         Name of the target position (string)
```

```
' Need to use Deck.FindLabwarePosition,
' in case labware is referred to by name instead of location
Target = CurDeck.FindLabwarePosition(e.EvaluateExpression(StepDictionary.Let.Target))

if IsEmpty(Target) or Target = "" then err.raise 1050, , "Target must be defined"

If Not IsObject(Pod.GrippedLabware) Then err.raise 1050, , "There is no labware in the
gripper."
Dim glw
set glw = Pod.GrippedLabware(Pod.GrippedLabware.Count - 1) ' The gripped labware is an
array with the bottom piece at the highest index

' Convert Source and Target names into objects
Dim Dst
Set Dst = Pipettor.Deck.Positions(Target)

Dim Z
Dim sx, sy, sz
' Dim gx, gy, gz, pd
'pd = CDbl(Pod.VariantDictionary.GetDefault("GripperDOffset", 0.0))

' Get the squeeze values
'Dim Unsqueeze
'Unsqueeze = CDbl(Pod.DAxisFromGripper(CDbl(pd) + CDbl(glw.Class.Unsqueeze)))

' Get the Pod gripper offsets
Dim px, py, pz
px = CDbl(Pod.GripperXOffset)
```

```
py = CDbl(Pod.GripperYOffset)
pz = CDbl(Pod.GripperZOffset)

' Enforce the labware's speed limit
'LWSpeedLimit = srcLabware.Class.SpeedLimit
'If LWSpeedLimit < OrigPodSpeed Then Pod.SetSpeed LWSpeedLimit

'Get height of destination position
Z = CDbl(Dst.Z(Pod))

' Get offsets from position to the labware at target position
Dim tx, ty, tz, dx, dy, dz
tx = CDbl(Dst.GetOneLabwareOffsetAtDepth(1, "X"))
ty = CDbl(Dst.GetOneLabwareOffsetAtDepth(1, "Y"))
tz = CDbl(Dst.GetOneLabwareOffsetAtDepth(1, "Z"))

' Get the Target Labware
Dim dstLabware
dx = 0
dy = 0
dz = 0
dstLabware = Null
if (Dst.StackDepth > 0) then
  Set dstLabware = Dst.GetLabwareAtDepth(1)
end if

' Get Destination Stack Offsets (really Per-Labware Offsets)
dx = CDbl(Dst.GetOneStackOffset(glw, dstLabware, "X"))
dy = CDbl(Dst.GetOneStackOffset(glw, dstLabware, "Y"))
```

```
dz = CDbl(Dst.GetOneStackOffset(glw, dstLabware, "Z"))

' Get the labware gripper offsets (added-in by MAG 3/7/2003)
World.Volatile.MyDest = Dst

'Dim gx, gy, gz, pd
'gx = CDbl(glw.Class.GripperXOffset)
'gy = CDbl(glw.Class.GripperYOffset)
'gz = CDbl(glw.Class.GripperZOffset)

pd = CDbl(Pod.VariantDictionary.GetDefault("GripperDOffset", 0.0))

' Get the labware gripper offsets and squeeze values
Dim gx, gy, gz, pd
Dim Unsqueeze
Select Case Pod.PodType
   Case "ATTILA"
      gx = CDbl(glw.Class.GripperInfo.MultiChannel.GripperXOffset)
      gy = CDbl(glw.Class.GripperInfo.MultiChannel.GripperYOffset)
      gz = CDbl(glw.Class.GripperInfo.MultiChannel.GripperZOffset)
      Unsqueeze = CDbl(Pod.DAxisFromGripper(CDbl(pd) +
CDbl(glw.Class.GripperInfo.MultiChannel.Unsqueeze)))
'tls.msg pod.podtype
'tls.msg gx & " " & gy & " " & gz & " "  & " " & Unsqueeze
   Case Else ' need to fill in code for NX Gripper
End Select
```

```
' Open Destination
Dst.Open True, 0

' Start Moving
Pod.ApproachPosition Target, CDbl(tx+dx+gx-px), CDbl(ty+dy+gy-py), CDbl(Z + tz+dz+gz-pz),
True

Pod.MoveZ CDbl(Z + tz + dz + gz - pz)

' Put Labware
Pod.AbsMove ,,,Unsqueeze

' Notify software the labware that was in the grippers is now at the target
' old v. 2.x syntax Pod.PutLabware Target
Pod.Gripper.PutLabwareObject Target

' Retract grippers
Pod.GripperRetract

' Close the destination position
Dst.Close True, 0

'Restore original pod speed now that the move is finished
'Pod.SetSpeed OrigPodSpeed

' Move to a safe height
Pod.MoveToSafe
```

Run droptips

Run cleanwashstation

  dspeed = 200

  fromtop = 0.6

  yoffset = 0.35

Run setstepdone

  wait = false

End

Else

End

If

If "MFX.NextStep = 16":

Then

Move To

Move "Pod2" to deck position "W1" at offset (0,0).

Move Labware

Move the top "2" plates at "Cryo1" to "Orbital1" using pod "Pod1".

Move To

Move "Pod1" to deck position "P2" at offset (0,0).

Run setstepdone

  wait = true

End

Else

End

End Loop

Group

If

If "MFX.UsePeltier":

Then

Incubate Pelt1 at =23C for 00:00:01

Position: Pelt1

Command: Incubate

Module Name: Peltier1

Set Temperature?: -1

Temperature: =23

Total Time: 00:00:01

End

Else

End

If

If "MFX.NextStep = 17":

Then

Script

Description:

Labware Pickup from Cryo1

Execute the following script code:

```
Option Explicit

' This script duplicates the functionality of the first half of the Move Labware step.

' Modified 3/7/2003 by MAG to lift the labware to a specific height off the deck

' (height measured at top of the labware) which was useful for a specific customer

' reading barcodes via their own reader, with labels always a certain distance from

' the top of the labware.


Set e = CreateObject("World.EngineObject")


Dim Source, Target, PodName

Dim Pod, CurDeck, OrigPodSpeed, LWSpeedLimit


' PodName        The name of the pod to use
```

```
PodName = StepDictionary.Let.PodName

if IsEmpty(PodName) or PodName = "" then err.raise 1050, , "PodName must be defined"

Set Pod = World.Devices.Pipettor1(PodName)

OrigPodSpeed = Pod.CurrentSpeed


Set CurDeck = World.Devices.Pipettor1.Deck


' Source         Name of the source position (string)

' Need to use Deck.FindLabwarePosition,

' in case labware is referred to by name instead of location

Source = CurDeck.FindLabwarePosition(e.EvaluateExpression(StepDictionary.Let.Source))


if IsEmpty(Source) or Source = "" then err.raise 1050, , "Source must be defined"


' Get the depth to grab a piece of labware at.

Dim Depth

Depth = e.EvaluateExpression(StepDictionary.Let.Depth)

if IsEmpty(Depth)         then err.raise 1050, , "Depth must be defined"

If Depth < 1              then err.raise 1050, , "Depth must be at least 1"


' Convert Source and Target names into objects

Dim Src

Set Src = Pipettor.Deck.Positions(Source)


' Get the Source Labware

Dim srcLabware

World.Volatile.Depth = Depth

Set srcLabware = Src.GetLabwareAtDepth(Depth)
```

```
' Get the source labware height  [DON'T NEED THIS ANYMORE]
' Dim srcLwHeight
' srcLwHeight = srcLabware.Class.Height

Dim Z
Z = CDbl(Src.Z(Pod))

' Get offsets from position to the labware at source position
Dim sx, sy, sz
sx = CDbl(Src.GetOneLabwareOffsetAtDepth(Depth, "X"))
sy = CDbl(Src.GetOneLabwareOffsetAtDepth(Depth, "Y"))
sz = CDbl(Src.GetOneLabwareOffsetAtDepth(Depth, "Z"))

pd = CDbl(Pod.VariantDictionary.GetDefault("GripperDOffset", 0.0))

' Get the labware gripper offsets and squeeze values
Dim gx, gy, gz, pd
Dim Squeeze, Unsqueeze
Select Case Pod.PodType
   Case "ATTILA"
      gx = CDbl(srcLabware.Class.GripperInfo.MultiChannel.GripperXOffset)
      gy = CDbl(srcLabware.Class.GripperInfo.MultiChannel.GripperYOffset)
      gz = CDbl(srcLabware.Class.GripperInfo.MultiChannel.GripperZOffset)
      Squeeze = CDbl(Pod.DAxisFromGripper(CDbl(pd) +
CDbl(srcLabware.Class.GripperInfo.MultiChannel.Squeeze)))
      Unsqueeze = CDbl(Pod.DAxisFromGripper(CDbl(pd) +
CDbl(srcLabware.Class.GripperInfo.MultiChannel.Unsqueeze)))
'tls.msg pod.podtype
```

```
'tls.msg gx & " " & gy & " " & gz & " " & Squeeze & " " & Unsqueeze
   Case Else ' need to fill in code for NX Gripper
End Select

' Get the Pod gripper offsets
Dim px, py, pz
px = CDbl(Pod.GripperXOffset)
py = CDbl(Pod.GripperYOffset)
pz = CDbl(Pod.GripperZOffset)

Dim LiftHeight, e

LiftHeight = e.EvaluateExpression(StepDictionary.Let.LiftHeight)

' Temporarily save the height of the move
World.Volatile.LiftHeight = LiftHeight

'Reserve the resources used, i.e., the source position and the Pod
World.Globals.ResourceReserver.ReserveResources Array(Src, Pod)

' Open the source position
Src.Open True, Depth

' Start Moving
Pod.ApproachPosition Source, sx+gx-px, sy+gy-py, Z + sz+gz-pz, False, Unsqueeze

' Extend the grippers
Pod.GripperExtend
```

```
' Grab Labware
Pod.AbsMove ,,,Squeeze

' Enforce the labware's speed limit
LWSpeedLimit = srcLabware.Class.SpeedLimit
If LWSpeedLimit < OrigPodSpeed Then Pod.SetSpeed LWSpeedLimit

' Notify software that the source labware is now in the grippers
' Version 2.x syntax: Pod.GetLabware Source, Depth
Pod.Gripper.GetLabwareObject Source, Depth

' Move to a safe height above the position (original function commented out)
' Pod.MoveToSafe

If LiftHeight < 0 Then err.raise 1050, ,  "Lift Height too low ; would result in downward
motion"

Pod.RelativeMoveAxes "Z", LiftHeight

' Close the source position
' Src.Close True, Depth

'Restore original pod speed now that the move is finished
Pod.SetSpeed OrigPodSpeed
```

Script

Description:

Execute the following script code:

Positions(Labware.Location("Cryo1")).Labware.ConfigureAmounts 0

Transfer

Using Pod2, execute the following transfer:

From: Buffer, with the following pattern:

```
   1
 A ()
 B ()
 C ()
 D ()
 E ()
 F ><
```

, Media

Proceed down first, then left to right.

Start from the beginning of the selection.

Set the mark at the last well transferred.

Use the following custom technique:

  Use the following pipetting template: OS Span-8 MultiDispense Slow Retract 2

  Calibration Offset: 2.54

  Calibration Slope: 1.033

  Minimum Pipetting Height: 0.25 mm

  Prewet: False

  Aspirate Blowout: True

  Follow Liquid: False

  Height: 0 mm from the liquid

  Mix: False

  Mix Aspirate Speed: 250µL/s

  Mix Aspirate Height: 1 mm from the liquid

  Mix Dispense Speed: 250µL/s

Mix Dispense Height: 25 mm from the liquid

Mix Count: 1

Mix Volume: 800 µL

Operation speed: 70µL/s

Tip Touch: False

Trailing Air Gap: True

Override Liquid Type Settings

Aspirate Delay: 10 ms

Aspirate Speed: 300 µL/s

Blowout Delay: 20 ms

Blowout Volume: 10 µL

Dispense Delay: 10 ms

Dispense Speed: 200 µL/s

Prewet Delay: 0 ms

Prewet Overage: 0 µL

Tip Touch Delay: 300 ms

Tip Touch Speed: 85 µL/s

Trailing Air Gap Volume: 0 µL

Override the technique height by moving to 1 mm from the bottom.

To: Cryo1, =MFX.P7VolMediaµL, sections specified by "=Wells()(MFX.NumSamples)", Media

Proceed down first, then left to right.

Start from the beginning of the selection.

Set the mark at the last well transferred.

Use the following custom technique:

Use the following pipetting template: OS Span-8 P1000 Blowout at LiqLevel 2

Calibration Offset: 2.54

Calibration Slope: 1.033

Minimum Pipetting Height: 0.5 mm

Prewet: False

Blowout: True

Follow Liquid: True

Height: 0 mm from the liquid

Mix: False

Mix Aspirate Speed: 100µL/s

Mix Aspirate Height: 2 mm from the bottom

Mix Dispense Speed: 100µL/s

Mix Dispense Height: 0.5 mm from the bottom

Mix Count: 1

Mix Volume: 10 µL

Operation speed: 70µL/s

Tip Touch: False

Override Liquid Type Settings

Aspirate Delay: 10 ms

Aspirate Speed: 30 µL/s

Blowout Delay: 10 ms

Blowout Volume: 10 µL

Dispense Delay: 10 ms

Dispense Speed: 200 µL/s

Prewet Delay: 0 ms

Prewet Overage: 0 µL

Tip Touch Delay: 300 ms

Tip Touch Speed: 85 µL/s

Trailing Air Gap Volume: 0 µL

Override the technique height by moving to 1 mm from the bottom.

Aspirate at most =MaxVolInTips µL per transfer for repeated dispensing.

Create 1 replicate(s) of each source well.

Load Span_8_1000uL_LLS onto the pod.

Keep the tips when finished.

Stop when finished with Destinations.

Probes specified by "=TwoTips()(MFX.NumSamples)" will be used.

Script

Description:

Labware Place to Cryo1

Execute the following script code:

```
Option Explicit

' This script duplicates the functionality of the Move Labware step.

' It has been modified to only place the labware after another step has picked it up.

Dim e

Set e = CreateObject("World.EngineObject")

Dim Target, PodName

Dim Pod, CurDeck, OrigPodSpeed, LWSpeedLimit


' PodName        The name of the pod to use

PodName = StepDictionary.Let.PodName

if IsEmpty(PodName) or PodName = "" then err.raise 1050, , "PodName must be defined"

Set Pod = World.Devices.Pipettor1(PodName)

OrigPodSpeed = Pod.CurrentSpeed


Set CurDeck = World.Devices.Pipettor1.Deck


' Target         Name of the target position (string)

' Need to use Deck.FindLabwarePosition,
```

```
' in case labware is referred to by name instead of location
Target = CurDeck.FindLabwarePosition(e.EvaluateExpression(StepDictionary.Let.Target))

if IsEmpty(Target) or Target = "" then err.raise 1050, , "Target must be defined"

If Not IsObject(Pod.GrippedLabware) Then err.raise 1050, , "There is no labware in the
gripper."
Dim glw
set glw = Pod.GrippedLabware(Pod.GrippedLabware.Count - 1) ' The gripped labware is an
array with the bottom piece at the highest index

' Convert Source and Target names into objects
Dim Dst
Set Dst = Pipettor.Deck.Positions(Target)

Dim Z
Dim sx, sy, sz
' Dim gx, gy, gz, pd
'pd = CDbl(Pod.VariantDictionary.GetDefault("GripperDOffset", 0.0))

' Get the squeeze values
'Dim Unsqueeze
'Unsqueeze = CDbl(Pod.DAxisFromGripper(CDbl(pd) + CDbl(glw.Class.Unsqueeze)))

' Get the Pod gripper offsets
Dim px, py, pz
px = CDbl(Pod.GripperXOffset)
py = CDbl(Pod.GripperYOffset)
pz = CDbl(Pod.GripperZOffset)
```

```
' Enforce the labware's speed limit
'LWSpeedLimit = srcLabware.Class.SpeedLimit
'If LWSpeedLimit < OrigPodSpeed Then Pod.SetSpeed LWSpeedLimit

'Get height of destination position
Z = CDbl(Dst.Z(Pod))

' Get offsets from position to the labware at target position
Dim tx, ty, tz, dx, dy, dz
tx = CDbl(Dst.GetOneLabwareOffsetAtDepth(1, "X"))
ty = CDbl(Dst.GetOneLabwareOffsetAtDepth(1, "Y"))
tz = CDbl(Dst.GetOneLabwareOffsetAtDepth(1, "Z"))

' Get the Target Labware
Dim dstLabware
dx = 0
dy = 0
dz = 0
dstLabware = Null
if (Dst.StackDepth > 0) then
  Set dstLabware = Dst.GetLabwareAtDepth(1)
end if

' Get Destination Stack Offsets (really Per-Labware Offsets)
dx = CDbl(Dst.GetOneStackOffset(glw, dstLabware, "X"))
dy = CDbl(Dst.GetOneStackOffset(glw, dstLabware, "Y"))
dz = CDbl(Dst.GetOneStackOffset(glw, dstLabware, "Z"))
```

```
' Get the labware gripper offsets (added-in by MAG 3/7/2003)
World.Volatile.MyDest = Dst

'Dim gx, gy, gz, pd
'gx = CDbl(glw.Class.GripperXOffset)
'gy = CDbl(glw.Class.GripperYOffset)
'gz = CDbl(glw.Class.GripperZOffset)

pd = CDbl(Pod.VariantDictionary.GetDefault("GripperDOffset", 0.0))

' Get the labware gripper offsets and squeeze values
Dim gx, gy, gz, pd
Dim Unsqueeze
Select Case Pod.PodType
   Case "ATTILA"
      gx = CDbl(glw.Class.GripperInfo.MultiChannel.GripperXOffset)
      gy = CDbl(glw.Class.GripperInfo.MultiChannel.GripperYOffset)
      gz = CDbl(glw.Class.GripperInfo.MultiChannel.GripperZOffset)
      Unsqueeze = CDbl(Pod.DAxisFromGripper(CDbl(pd) +
CDbl(glw.Class.GripperInfo.MultiChannel.Unsqueeze)))
'tls.msg pod.podtype
'tls.msg gx & " " & gy & " " & gz & " "  & " " & Unsqueeze
   Case Else ' need to fill in code for NX Gripper
End Select
```

```
' Open Destination
Dst.Open True, 0

' Start Moving
Pod.ApproachPosition Target, CDbl(tx+dx+gx-px), CDbl(ty+dy+gy-py), CDbl(Z + tz+dz+gz-pz),
True

Pod.MoveZ CDbl(Z + tz + dz + gz - pz)

' Put Labware
Pod.AbsMove ,,,Unsqueeze

' Notify software the labware that was in the grippers is now at the target
' old v. 2.x syntax Pod.PutLabware Target
Pod.Gripper.PutLabwareObject Target

' Retract grippers
Pod.GripperRetract

' Close the destination position
Dst.Close True, 0

'Restore original pod speed now that the move is finished
'Pod.SetSpeed OrigPodSpeed

' Move to a safe height
Pod.MoveToSafe
---------------------------------------------------------------------------
```

```
Run droptips
---------------------------------------------------------------------------
Run setstepdone
  wait = true
---------------------------------------------------------------------------
End
---------------------------------------------------------------------------
Else
---------------------------------------------------------------------------
End
---------------------------------------------------------------------------
If
If "MFX.NextStep = 18":
---------------------------------------------------------------------------
Then
---------------------------------------------------------------------------
Move Labware
Move the top "1" plates at "Cryo2" to "TR2" using pod "Pod1".
---------------------------------------------------------------------------
Run setstepdone
  wait = true
---------------------------------------------------------------------------
End
---------------------------------------------------------------------------
Else
---------------------------------------------------------------------------
End
---------------------------------------------------------------------------
If
```

If "MFX.NextStep = 19":

Then

Script

Description:

Labware Pickup from TB1

Execute the following script code:

```
Option Explicit

' This script duplicates the functionality of the first half of the Move Labware step.

' Modified 3/7/2003 by MAG to lift the labware to a specific height off the deck

' (height measured at top of the labware) which was useful for a specific customer

' reading barcodes via their own reader, with labels always a certain distance from

' the top of the labware.


Set e = CreateObject("World.EngineObject")


Dim Source, Target, PodName

Dim Pod, CurDeck, OrigPodSpeed, LWSpeedLimit


' PodName        The name of the pod to use

PodName = StepDictionary.Let.PodName

if IsEmpty(PodName) or PodName = "" then err.raise 1050, , "PodName must be defined"

Set Pod = World.Devices.Pipettor1(PodName)

OrigPodSpeed = Pod.CurrentSpeed


Set CurDeck = World.Devices.Pipettor1.Deck
```

```
' Source         Name of the source position (string)

' Need to use Deck.FindLabwarePosition,

' in case labware is referred to by name instead of location

Source = CurDeck.FindLabwarePosition(e.EvaluateExpression(StepDictionary.Let.Source))


if IsEmpty(Source) or Source = "" then err.raise 1050, , "Source must be defined"


' Get the depth to grab a piece of labware at.

Dim Depth

Depth = e.EvaluateExpression(StepDictionary.Let.Depth)

if IsEmpty(Depth)         then err.raise 1050, , "Depth must be defined"

If Depth < 1              then err.raise 1050, , "Depth must be at least 1"


' Convert Source and Target names into objects

Dim Src

Set Src = Pipettor.Deck.Positions(Source)


' Get the Source Labware

Dim srcLabware

World.Volatile.Depth = Depth

Set srcLabware = Src.GetLabwareAtDepth(Depth)


' Get the source labware height  [DON'T NEED THIS ANYMORE]

' Dim srcLwHeight

' srcLwHeight = srcLabware.Class.Height


Dim Z

Z = CDbl(Src.Z(Pod))
```

```
' Get offsets from position to the labware at source position
Dim sx, sy, sz
sx = CDbl(Src.GetOneLabwareOffsetAtDepth(Depth, "X"))
sy = CDbl(Src.GetOneLabwareOffsetAtDepth(Depth, "Y"))
sz = CDbl(Src.GetOneLabwareOffsetAtDepth(Depth, "Z"))

pd = CDbl(Pod.VariantDictionary.GetDefault("GripperDOffset", 0.0))

' Get the labware gripper offsets and squeeze values
Dim gx, gy, gz, pd
Dim Squeeze, Unsqueeze
Select Case Pod.PodType
   Case "ATTILA"
      gx = CDbl(srcLabware.Class.GripperInfo.MultiChannel.GripperXOffset)
      gy = CDbl(srcLabware.Class.GripperInfo.MultiChannel.GripperYOffset)
      gz = CDbl(srcLabware.Class.GripperInfo.MultiChannel.GripperZOffset)
      Squeeze = CDbl(Pod.DAxisFromGripper(CDbl(pd) +
CDbl(srcLabware.Class.GripperInfo.MultiChannel.Squeeze)))
      Unsqueeze = CDbl(Pod.DAxisFromGripper(CDbl(pd) +
CDbl(srcLabware.Class.GripperInfo.MultiChannel.Unsqueeze)))
'tls.msg pod.podtype
'tls.msg gx & " " & gy & " " & gz & " " & Squeeze & " " & Unsqueeze
   Case Else ' need to fill in code for NX Gripper
End Select

' Get the Pod gripper offsets
Dim px, py, pz
px = CDbl(Pod.GripperXOffset)
```

```
py = CDbl(Pod.GripperYOffset)
pz = CDbl(Pod.GripperZOffset)

Dim LiftHeight, e

LiftHeight = e.EvaluateExpression(StepDictionary.Let.LiftHeight)

' Temporarily save the height of the move
World.Volatile.LiftHeight = LiftHeight

'Reserve the resources used, i.e., the source position and the Pod
World.Globals.ResourceReserver.ReserveResources Array(Src, Pod)

' Open the source position
Src.Open True, Depth

' Start Moving
Pod.ApproachPosition Source, sx+gx-px, sy+gy-py, Z + sz+gz-pz, False, Unsqueeze

' Extend the grippers
Pod.GripperExtend

' Grab Labware
Pod.AbsMove ,,,Squeeze

' Enforce the labware's speed limit
LWSpeedLimit = srcLabware.Class.SpeedLimit
If LWSpeedLimit < OrigPodSpeed Then Pod.SetSpeed LWSpeedLimit
```

' Notify software that the source labware is now in the grippers

' Version 2.x syntax: Pod.GetLabware Source, Depth

Pod.Gripper.GetLabwareObject Source, Depth

' Move to a safe height above the position (original function commented out)

' Pod.MoveToSafe

If LiftHeight < 0 Then err.raise 1050, , "Lift Height too low ; would result in downward motion"

Pod.RelativeMoveAxes "Z", LiftHeight

' Close the source position

' Src.Close True, Depth

'Restore original pod speed now that the move is finished

Pod.SetSpeed OrigPodSpeed

Span-8 New Tips

Get new TB1 tips for probes specified by "=OneTip()(MFX.NumSamples)" on Pod2.

Script

Description:

Labware Place to TB1

Execute the following script code:

Option Explicit

' This script duplicates the functionality of the Move Labware step.

' It has been modified to only place the labware after another step has picked it up.

```
Dim e

Set e = CreateObject("World.EngineObject")

Dim Target, PodName

Dim Pod, CurDeck, OrigPodSpeed, LWSpeedLimit


' PodName        The name of the pod to use

PodName = StepDictionary.Let.PodName

if IsEmpty(PodName) or PodName = "" then err.raise 1050, , "PodName must be defined"

Set Pod = World.Devices.Pipettor1(PodName)

OrigPodSpeed = Pod.CurrentSpeed


Set CurDeck = World.Devices.Pipettor1.Deck


' Target         Name of the target position (string)

' Need to use Deck.FindLabwarePosition,

' in case labware is referred to by name instead of location

Target = CurDeck.FindLabwarePosition(e.EvaluateExpression(StepDictionary.Let.Target))


if IsEmpty(Target) or Target = "" then err.raise 1050, , "Target must be defined"


If Not IsObject(Pod.GrippedLabware) Then err.raise 1050, , "There is no labware in the
gripper."

Dim glw

set glw = Pod.GrippedLabware(Pod.GrippedLabware.Count - 1) ' The gripped labware is an
array with the bottom piece at the highest index


' Convert Source and Target names into objects

Dim Dst

Set Dst = Pipettor.Deck.Positions(Target)
```

```
Dim Z

Dim sx, sy, sz

' Dim gx, gy, gz, pd

'pd = CDbl(Pod.VariantDictionary.GetDefault("GripperDOffset", 0.0))


' Get the squeeze values

'Dim Unsqueeze

'Unsqueeze = CDbl(Pod.DAxisFromGripper(CDbl(pd) + CDbl(glw.Class.Unsqueeze)))


' Get the Pod gripper offsets

Dim px, py, pz

px = CDbl(Pod.GripperXOffset)

py = CDbl(Pod.GripperYOffset)

pz = CDbl(Pod.GripperZOffset)


' Enforce the labware's speed limit

'LWSpeedLimit = srcLabware.Class.SpeedLimit

'If LWSpeedLimit < OrigPodSpeed Then Pod.SetSpeed LWSpeedLimit


'Get height of destination position

Z = CDbl(Dst.Z(Pod))


' Get offsets from position to the labware at target position

Dim tx, ty, tz, dx, dy, dz

tx = CDbl(Dst.GetOneLabwareOffsetAtDepth(1, "X"))

ty = CDbl(Dst.GetOneLabwareOffsetAtDepth(1, "Y"))

tz = CDbl(Dst.GetOneLabwareOffsetAtDepth(1, "Z"))
```

```
' Get the Target Labware
Dim dstLabware
dx = 0
dy = 0
dz = 0
dstLabware = Null
if (Dst.StackDepth > 0) then
  Set dstLabware = Dst.GetLabwareAtDepth(1)
end if

' Get Destination Stack Offsets (really Per-Labware Offsets)
dx = CDbl(Dst.GetOneStackOffset(glw, dstLabware, "X"))
dy = CDbl(Dst.GetOneStackOffset(glw, dstLabware, "Y"))
dz = CDbl(Dst.GetOneStackOffset(glw, dstLabware, "Z"))

' Get the labware gripper offsets (added-in by MAG 3/7/2003)
World.Volatile.MyDest = Dst

'Dim gx, gy, gz, pd
'gx = CDbl(glw.Class.GripperXOffset)
'gy = CDbl(glw.Class.GripperYOffset)
'gz = CDbl(glw.Class.GripperZOffset)

pd = CDbl(Pod.VariantDictionary.GetDefault("GripperDOffset", 0.0))

' Get the labware gripper offsets and squeeze values
Dim gx, gy, gz, pd
Dim Unsqueeze
```

```
Select Case Pod.PodType
   Case "ATTILA"
      gx = CDbl(glw.Class.GripperInfo.MultiChannel.GripperXOffset)
      gy = CDbl(glw.Class.GripperInfo.MultiChannel.GripperYOffset)
      gz = CDbl(glw.Class.GripperInfo.MultiChannel.GripperZOffset)
      Unsqueeze = CDbl(Pod.DAxisFromGripper(CDbl(pd) +
CDbl(glw.Class.GripperInfo.MultiChannel.Unsqueeze)))
'tls.msg pod.podtype
'tls.msg gx & " " & gy & " " & gz & " "  & " " & Unsqueeze
   Case Else ' need to fill in code for NX Gripper
End Select




' Open Destination
Dst.Open True, 0

' Start Moving
Pod.ApproachPosition Target, CDbl(tx+dx+gx-px), CDbl(ty+dy+gy-py), CDbl(Z + tz+dz+gz-pz),
True

Pod.MoveZ CDbl(Z + tz + dz + gz - pz)

' Put Labware
Pod.AbsMove ,,,Unsqueeze

' Notify software the labware that was in the grippers is now at the target
```

```
' old v. 2.x syntax Pod.PutLabware Target

Pod.Gripper.PutLabwareObject Target


' Retract grippers

Pod.GripperRetract


' Close the destination position

Dst.Close True, 0


'Restore original pod speed now that the move is finished

'Pod.SetSpeed OrigPodSpeed


' Move to a safe height

Pod.MoveToSafe
```

Script

Description:

Labware Pickup from Cryo1

Execute the following script code:

```
Option Explicit

' This script duplicates the functionality of the first half of the Move Labware step.

' Modified 3/7/2003 by MAG to lift the labware to a specific height off the deck

' (height measured at top of the labware) which was useful for a specific customer

' reading barcodes via their own reader, with labels always a certain distance from

' the top of the labware.


Set e = CreateObject("World.EngineObject")


Dim Source, Target, PodName
```

```
Dim Pod, CurDeck, OrigPodSpeed, LWSpeedLimit

' PodName        The name of the pod to use
PodName = StepDictionary.Let.PodName
if IsEmpty(PodName) or PodName = "" then err.raise 1050, , "PodName must be defined"
Set Pod = World.Devices.Pipettor1(PodName)
OrigPodSpeed = Pod.CurrentSpeed

Set CurDeck = World.Devices.Pipettor1.Deck

' Source         Name of the source position (string)
' Need to use Deck.FindLabwarePosition,
' in case labware is referred to by name instead of location
Source = CurDeck.FindLabwarePosition(e.EvaluateExpression(StepDictionary.Let.Source))

if IsEmpty(Source) or Source = "" then err.raise 1050, , "Source must be defined"

' Get the depth to grab a piece of labware at.
Dim Depth
Depth = e.EvaluateExpression(StepDictionary.Let.Depth)
if IsEmpty(Depth)         then err.raise 1050, , "Depth must be defined"
If Depth < 1              then err.raise 1050, , "Depth must be at least 1"

' Convert Source and Target names into objects
Dim Src
Set Src = Pipettor.Deck.Positions(Source)

' Get the Source Labware
```

```
Dim srcLabware

World.Volatile.Depth = Depth

Set srcLabware = Src.GetLabwareAtDepth(Depth)


' Get the source labware height  [DON'T NEED THIS ANYMORE]

' Dim srcLwHeight

' srcLwHeight = srcLabware.Class.Height


Dim Z

Z = CDbl(Src.Z(Pod))


' Get offsets from position to the labware at source position

Dim sx, sy, sz

sx = CDbl(Src.GetOneLabwareOffsetAtDepth(Depth, "X"))

sy = CDbl(Src.GetOneLabwareOffsetAtDepth(Depth, "Y"))

sz = CDbl(Src.GetOneLabwareOffsetAtDepth(Depth, "Z"))


pd = CDbl(Pod.VariantDictionary.GetDefault("GripperDOffset", 0.0))


' Get the labware gripper offsets and squeeze values

Dim gx, gy, gz, pd

Dim Squeeze, Unsqueeze

Select Case Pod.PodType

   Case "ATTILA"

      gx = CDbl(srcLabware.Class.GripperInfo.MultiChannel.GripperXOffset)

      gy = CDbl(srcLabware.Class.GripperInfo.MultiChannel.GripperYOffset)

      gz = CDbl(srcLabware.Class.GripperInfo.MultiChannel.GripperZOffset)

      Squeeze = CDbl(Pod.DAxisFromGripper(CDbl(pd) +

CDbl(srcLabware.Class.GripperInfo.MultiChannel.Squeeze)))
```

```
      Unsqueeze = CDbl(Pod.DAxisFromGripper(CDbl(pd) +
CDbl(srcLabware.Class.GripperInfo.MultiChannel.Unsqueeze)))
'tls.msg pod.podtype
'tls.msg gx & " " & gy & " " & gz & " " & Squeeze & " " & Unsqueeze
   Case Else ' need to fill in code for NX Gripper
End Select

' Get the Pod gripper offsets
Dim px, py, pz
px = CDbl(Pod.GripperXOffset)
py = CDbl(Pod.GripperYOffset)
pz = CDbl(Pod.GripperZOffset)

Dim LiftHeight, e

LiftHeight = e.EvaluateExpression(StepDictionary.Let.LiftHeight)

' Temporarily save the height of the move
World.Volatile.LiftHeight = LiftHeight

'Reserve the resources used, i.e., the source position and the Pod
World.Globals.ResourceReserver.ReserveResources Array(Src, Pod)

' Open the source position
Src.Open True, Depth

' Start Moving
Pod.ApproachPosition Source, sx+gx-px, sy+gy-py, Z + sz+gz-pz, False, Unsqueeze
```

```
' Extend the grippers
Pod.GripperExtend

' Grab Labware
Pod.AbsMove ,,,Squeeze

' Enforce the labware's speed limit
LWSpeedLimit = srcLabware.Class.SpeedLimit
If LWSpeedLimit < OrigPodSpeed Then Pod.SetSpeed LWSpeedLimit

' Notify software that the source labware is now in the grippers
' Version 2.x syntax: Pod.GetLabware Source, Depth
Pod.Gripper.GetLabwareObject Source, Depth

' Move to a safe height above the position (original function commented out)
' Pod.MoveToSafe

If LiftHeight < 0 Then err.raise 1050, ,  "Lift Height too low ; would result in downward
motion"

Pod.RelativeMoveAxes "Z", LiftHeight

' Close the source position
' Src.Close True, Depth

'Restore original pod speed now that the move is finished
Pod.SetSpeed OrigPodSpeed
```

Transfer

Using Pod2, execute the following transfer:

From: Cryo2, with the following pattern:

	1	2	3	4	5	6
A	()	()	()	()	()	()
B	()	()	()	()	()	()
C	()	()	()	()	()	()
D	()	()	()	><	()	()

, detachabead

Proceed down first, then left to right.

Start from the beginning of the selection.

Set the mark at the last well transferred.

Use the following custom technique:

Use the following pipetting template: OS Span-8 MultiDispense Slow Retract 2

Calibration Offset: 0

Calibration Slope: 1

Minimum Pipetting Height: 0 mm

Prewet: False

Aspirate Blowout: False

Follow Liquid: False

Height: 0 mm from the bottom

Mix: False

Mix Aspirate Speed: 200µL/s

Mix Aspirate Height: 0.1 mm from the bottom

Mix Dispense Speed: 200µL/s

Mix Dispense Height: 0.1 mm from the bottom

Mix Count: 1

Mix Volume: 80 µL

Operation speed: 70µL/s

Tip Touch: False

Trailing Air Gap: False

Override Liquid Type Settings

Aspirate Delay: 1 ms

Aspirate Speed: 100 µL/s

Blowout Delay: 1 ms

Blowout Volume: 10 µL

Dispense Delay: 1 ms

Dispense Speed: 250 µL/s

Prewet Delay: 0 ms

Prewet Overage: 10 µL

Tip Touch Delay: 100 ms

Tip Touch Speed: 80 µL/s

Trailing Air Gap Volume: 0 µL

Override the technique height by moving to 0.5 mm from the bottom.

To: Cryo1, =MFX.P7VolDABµL, sections specified by "=Wells()(MFX.NumSamples)", detachabead

Proceed down first, then left to right.

Start from the beginning of the selection.

Set the mark at the last well transferred.

Use the following custom technique:

Use the following pipetting template: Step3_P200 MixXfer 2

Calibration Offset: 0

Calibration Slope: 1

Minimum Pipetting Height: 0.2 mm

Prewet: True

Blowout: False

Follow Liquid: True

Height: 0.5 mm from the bottom

Mix: False

Mix Aspirate Speed: 200µL/s

Mix Aspirate Height: 1 mm from the bottom

Mix Dispense Speed: 200µL/s

Mix Dispense Height: 1 mm from the bottom

Mix Count: 2

Mix Volume: 200 µL

Operation speed: 70µL/s

Tip Touch: False

Override Liquid Type Settings

Aspirate Delay: 1 ms

Aspirate Speed: 400 µL/s

Blowout Delay: 1 ms

Blowout Volume: 10 µL

Dispense Delay: 1 ms

Dispense Speed: 250 µL/s

Prewet Delay: 0 ms

Prewet Overage: 10 µL

Tip Touch Delay: 100 ms

Tip Touch Speed: 80 µL/s

Trailing Air Gap Volume: 0 µL

Override the technique height by moving to 3 mm from the bottom.

Dispense up to 6 time(s) per draw.

Create 1 replicate(s) of each source well.

Keep the current tips

Unload the tips when finished.

Stop when finished with Destinations.

Probes specified by "=OneTip()(MFX.NumSamples)" will be used.

Script

Description:

Labware Place to Cryo1

Execute the following script code:

```
Option Explicit

' This script duplicates the functionality of the Move Labware step.

' It has been modified to only place the labware after another step has picked it up.

Dim e

Set e = CreateObject("World.EngineObject")

Dim Target, PodName

Dim Pod, CurDeck, OrigPodSpeed, LWSpeedLimit


' PodName        The name of the pod to use

PodName = StepDictionary.Let.PodName

if IsEmpty(PodName) or PodName = "" then err.raise 1050, , "PodName must be defined"

Set Pod = World.Devices.Pipettor1(PodName)

OrigPodSpeed = Pod.CurrentSpeed


Set CurDeck = World.Devices.Pipettor1.Deck


' Target         Name of the target position (string)

' Need to use Deck.FindLabwarePosition,

' in case labware is referred to by name instead of location

Target = CurDeck.FindLabwarePosition(e.EvaluateExpression(StepDictionary.Let.Target))


if IsEmpty(Target) or Target = "" then err.raise 1050, , "Target must be defined"
```

```
If Not IsObject(Pod.GrippedLabware) Then err.raise 1050, , "There is no labware in the
gripper."
Dim glw
set glw = Pod.GrippedLabware(Pod.GrippedLabware.Count - 1) ' The gripped labware is an
array with the bottom piece at the highest index

' Convert Source and Target names into objects
Dim Dst
Set Dst = Pipettor.Deck.Positions(Target)

Dim Z
Dim sx, sy, sz
' Dim gx, gy, gz, pd
'pd = CDbl(Pod.VariantDictionary.GetDefault("GripperDOffset", 0.0))

' Get the squeeze values
'Dim Unsqueeze
'Unsqueeze = CDbl(Pod.DAxisFromGripper(CDbl(pd) + CDbl(glw.Class.Unsqueeze)))

' Get the Pod gripper offsets
Dim px, py, pz
px = CDbl(Pod.GripperXOffset)
py = CDbl(Pod.GripperYOffset)
pz = CDbl(Pod.GripperZOffset)

' Enforce the labware's speed limit
'LWSpeedLimit = srcLabware.Class.SpeedLimit
```

```
'If LWSpeedLimit < OrigPodSpeed Then Pod.SetSpeed LWSpeedLimit

'Get height of destination position
Z = CDbl(Dst.Z(Pod))

' Get offsets from position to the labware at target position
Dim tx, ty, tz, dx, dy, dz
tx = CDbl(Dst.GetOneLabwareOffsetAtDepth(1, "X"))
ty = CDbl(Dst.GetOneLabwareOffsetAtDepth(1, "Y"))
tz = CDbl(Dst.GetOneLabwareOffsetAtDepth(1, "Z"))

' Get the Target Labware
Dim dstLabware
dx = 0
dy = 0
dz = 0
dstLabware = Null
if (Dst.StackDepth > 0) then
  Set dstLabware = Dst.GetLabwareAtDepth(1)
end if

' Get Destination Stack Offsets (really Per-Labware Offsets)
dx = CDbl(Dst.GetOneStackOffset(glw, dstLabware, "X"))
dy = CDbl(Dst.GetOneStackOffset(glw, dstLabware, "Y"))
dz = CDbl(Dst.GetOneStackOffset(glw, dstLabware, "Z"))

' Get the labware gripper offsets (added-in by MAG 3/7/2003)
World.Volatile.MyDest = Dst
```

```
'Dim gx, gy, gz, pd

'gx = CDbl(glw.Class.GripperXOffset)

'gy = CDbl(glw.Class.GripperYOffset)

'gz = CDbl(glw.Class.GripperZOffset)


pd = CDbl(Pod.VariantDictionary.GetDefault("GripperDOffset", 0.0))


' Get the labware gripper offsets and squeeze values

Dim gx, gy, gz, pd

Dim Unsqueeze

Select Case Pod.PodType

   Case "ATTILA"

      gx = CDbl(glw.Class.GripperInfo.MultiChannel.GripperXOffset)

      gy = CDbl(glw.Class.GripperInfo.MultiChannel.GripperYOffset)

      gz = CDbl(glw.Class.GripperInfo.MultiChannel.GripperZOffset)

      Unsqueeze = CDbl(Pod.DAxisFromGripper(CDbl(pd) +

CDbl(glw.Class.GripperInfo.MultiChannel.Unsqueeze)))

'tls.msg pod.podtype

'tls.msg gx & " " & gy & " " & gz & " "  & " " & Unsqueeze

   Case Else ' need to fill in code for NX Gripper

End Select






' Open Destination

Dst.Open True, 0
```

```
' Start Moving

Pod.ApproachPosition Target, CDbl(tx+dx+gx-px), CDbl(ty+dy+gy-py), CDbl(Z + tz+dz+gz-pz),
True

Pod.MoveZ CDbl(Z + tz + dz + gz - pz)

' Put Labware
Pod.AbsMove ,,,Unsqueeze

' Notify software the labware that was in the grippers is now at the target
' old v. 2.x syntax Pod.PutLabware Target
Pod.Gripper.PutLabwareObject Target

' Retract grippers
Pod.GripperRetract

' Close the destination position
Dst.Close True, 0

'Restore original pod speed now that the move is finished
'Pod.SetSpeed OrigPodSpeed

' Move to a safe height
Pod.MoveToSafe
---------------------------------------------------------------------------
Device Action
Send the following command to "OrbitalShakerALP0": "Timed Shake".
Parameters: 700, 1, 10, CounterClockwise
---------------------------------------------------------------------------
```

```
Run setstepdone

  wait = true

---------------------------------------------------------------------------

End

---------------------------------------------------------------------------

Else

---------------------------------------------------------------------------

End

---------------------------------------------------------------------------

If

If "MFX.NextStep = 20":

---------------------------------------------------------------------------

Then

---------------------------------------------------------------------------

Configure Tips

Description: Step 3b

Caption: Configure Tips

Code: ' How many tips need to be loaded? Depends on NumSamples

Select Case MFX.NumSamples

   Case 1

      Extend "LoadTips", "1,2,3,4"

   Case 2

      Extend "LoadTips", "1,2,3,4,5,6,7,8"

   Case 3

      Extend "LoadTips", "1,2,3,4,5,6"

End Select

Prompt: [IDispatch]

_Commented: 0
```

Group:

Incubate/Tipmix

Script

Description:

Execute the following script code:

```
If IsRunning Then

   PG.StallUntilETSIs 0

   TLS.SetTimer "Incubating...", MFX.P7IncTime

End If
```

Loop

Loop from "nTipMix" = "1" to "999", incrementing by "1".

If

If "MFX.P7DoTipMix":

Then

Run getnewtips

  pattern = =OneTip()(MFX.NumSamples)

Configure

Bitmap: C:\Program Files\Biomek Software\Bitmaps\Vials.bmp

Description:

Caption: Configure

```
Code: 'what is the mix volume?

mixvol = (MFX.P7VolDAB + MFX.P7VolMedia) * 0.8


'determine the correct height

Set LW = Positions(Labware.Location("Cryo1")).Labware

aspheightcm = LW.HeightFromVolume(mixvol, 1)

              ' this is the height in CM

              ' needs to be multiplied by 10 because the step expects MM


Extend "MixWells", xwells

Extend "UseTips",  tips

Extend "VolMix",   mixvol

Extend "ZAsp",     aspheightcm * 10 ' convert cm to mm

Prompt: [IDispatch]

Tooltip: Scripted Let

---------------------------------------------------------------------------

Script

Description:

Labware Pickup from Cryo1

Execute the following script code:

Option Explicit

' This script duplicates the functionality of the first half of the Move Labware step.

' Modified 3/7/2003 by MAG to lift the labware to a specific height off the deck

' (height measured at top of the labware) which was useful for a specific customer

' reading barcodes via their own reader, with labels always a certain distance from

' the top of the labware.


Set e = CreateObject("World.EngineObject")
```

```
Dim Source, Target, PodName
Dim Pod, CurDeck, OrigPodSpeed, LWSpeedLimit

' PodName        The name of the pod to use
PodName = StepDictionary.Let.PodName
if IsEmpty(PodName) or PodName = "" then err.raise 1050, , "PodName must be defined"
Set Pod = World.Devices.Pipettor1(PodName)
OrigPodSpeed = Pod.CurrentSpeed

Set CurDeck = World.Devices.Pipettor1.Deck

' Source         Name of the source position (string)
' Need to use Deck.FindLabwarePosition,
' in case labware is referred to by name instead of location
Source = CurDeck.FindLabwarePosition(e.EvaluateExpression(StepDictionary.Let.Source))

if IsEmpty(Source) or Source = "" then err.raise 1050, , "Source must be defined"

' Get the depth to grab a piece of labware at.
Dim Depth
Depth = e.EvaluateExpression(StepDictionary.Let.Depth)
if IsEmpty(Depth)         then err.raise 1050, , "Depth must be defined"
If Depth < 1              then err.raise 1050, , "Depth must be at least 1"

' Convert Source and Target names into objects
Dim Src
Set Src = Pipettor.Deck.Positions(Source)
```

```
' Get the Source Labware
Dim srcLabware
World.Volatile.Depth = Depth
Set srcLabware = Src.GetLabwareAtDepth(Depth)

' Get the source labware height  [DON'T NEED THIS ANYMORE]
' Dim srcLwHeight
' srcLwHeight = srcLabware.Class.Height

Dim Z
Z = CDbl(Src.Z(Pod))

' Get offsets from position to the labware at source position
Dim sx, sy, sz
sx = CDbl(Src.GetOneLabwareOffsetAtDepth(Depth, "X"))
sy = CDbl(Src.GetOneLabwareOffsetAtDepth(Depth, "Y"))
sz = CDbl(Src.GetOneLabwareOffsetAtDepth(Depth, "Z"))

pd = CDbl(Pod.VariantDictionary.GetDefault("GripperDOffset", 0.0))

' Get the labware gripper offsets and squeeze values
Dim gx, gy, gz, pd
Dim Squeeze, Unsqueeze
Select Case Pod.PodType
   Case "ATTILA"
      gx = CDbl(srcLabware.Class.GripperInfo.MultiChannel.GripperXOffset)
      gy = CDbl(srcLabware.Class.GripperInfo.MultiChannel.GripperYOffset)
      gz = CDbl(srcLabware.Class.GripperInfo.MultiChannel.GripperZOffset)
```

```
      Squeeze = CDbl(Pod.DAxisFromGripper(CDbl(pd) +
CDbl(srcLabware.Class.GripperInfo.MultiChannel.Squeeze)))
      Unsqueeze = CDbl(Pod.DAxisFromGripper(CDbl(pd) +
CDbl(srcLabware.Class.GripperInfo.MultiChannel.Unsqueeze)))
'tls.msg pod.podtype
'tls.msg gx & " " & gy & " " & gz & " " & Squeeze & " " & Unsqueeze
   Case Else ' need to fill in code for NX Gripper
End Select

' Get the Pod gripper offsets
Dim px, py, pz
px = CDbl(Pod.GripperXOffset)
py = CDbl(Pod.GripperYOffset)
pz = CDbl(Pod.GripperZOffset)

Dim LiftHeight, e

LiftHeight = e.EvaluateExpression(StepDictionary.Let.LiftHeight)

' Temporarily save the height of the move
World.Volatile.LiftHeight = LiftHeight

'Reserve the resources used, i.e., the source position and the Pod
World.Globals.ResourceReserver.ReserveResources Array(Src, Pod)

' Open the source position
Src.Open True, Depth

' Start Moving
```

```
Pod.ApproachPosition Source, sx+gx-px, sy+gy-py, Z + sz+gz-pz, False, Unsqueeze

' Extend the grippers
Pod.GripperExtend

' Grab Labware
Pod.AbsMove ,,,Squeeze

' Enforce the labware's speed limit
LWSpeedLimit = srcLabware.Class.SpeedLimit
If LWSpeedLimit < OrigPodSpeed Then Pod.SetSpeed LWSpeedLimit

' Notify software that the source labware is now in the grippers
' Version 2.x syntax: Pod.GetLabware Source, Depth
Pod.Gripper.GetLabwareObject Source, Depth

' Move to a safe height above the position (original function commented out)
' Pod.MoveToSafe

If LiftHeight < 0 Then err.raise 1050, ,  "Lift Height too low ; would result in downward
motion"

Pod.RelativeMoveAxes "Z", LiftHeight

' Close the source position
' Src.Close True, Depth

'Restore original pod speed now that the move is finished
```

Pod.SetSpeed OrigPodSpeed

Transfer

Using Pod2, execute the following transfer:

From: Cryo1, sections specified by "=Wells()(MFX.NumSamples)", BeadsplusCells

Proceed down first, then left to right.

Start from the beginning of the selection.

Do not set the mark.

Use the following custom technique:

  Use the following pipetting template: OS Span-8 P1000 MixOnly 2

  Calibration Offset: 2.54

  Calibration Slope: 1.033

  Minimum Pipetting Height: =ZMinCryo mm

  Prewet: False

  Aspirate Blowout: True

  Follow Liquid: True

  Height: 0 mm from the liquid

  Mix: True

  Mix Aspirate Speed: =MFX.P7MixSpeedµL/s

  Mix Aspirate Height: =ZAsp mm from the liquid

  Mix Dispense Speed: =MFX.P7MixSpeedµL/s

  Mix Dispense Height: 2 mm from the liquid

  Mix Count: =MFX.P7MixCycles

  Mix Volume: =VolMix µL

  Operation speed: 70µL/s

  Tip Touch: True

  Trailing Air Gap: False

  Override Liquid Type Settings

    Aspirate Delay: 10 ms

Aspirate Speed: 100 µL/s

Blowout Delay: 500 ms

Blowout Volume: 20 µL

Dispense Delay: 20 ms

Dispense Speed: 200 µL/s

Prewet Delay: 200 ms

Prewet Overage: 0 µL

Tip Touch Delay: 300 ms

Tip Touch Speed: 70 µL/s

Trailing Air Gap Volume: 10 µL

Override the technique height by moving to =ZAsp mm from the bottom.

To: Cryo1, 0µL, sections specified by "=Wells()(MFX.NumSamples)", BeadsplusCells

Proceed down first, then left to right.

Start from the beginning of the selection.

Do not set the mark.

Use the following custom technique:

Use the following pipetting template: OS Span-8 P1000 MixOnly 2

Calibration Offset: 2.54

Calibration Slope: 1.033

Minimum Pipetting Height: 0.5 mm

Prewet: False

Blowout: False

Follow Liquid: False

Height: 0 mm from the liquid

Mix: False

Mix Aspirate Speed: 100µL/s

Mix Aspirate Height: 0 mm from the liquid

Mix Dispense Speed: 100µL/s

Mix Dispense Height: 0 mm from the liquid

Mix Count: 1

Mix Volume: 900 µL

Operation speed: 70µL/s

Tip Touch: False

Override Liquid Type Settings

Aspirate Delay: 10 ms

Aspirate Speed: 300 µL/s

Blowout Delay: 10 ms

Blowout Volume: 20 µL

Dispense Delay: 20 ms

Dispense Speed: 200 µL/s

Prewet Delay: 0 ms

Prewet Overage: 0 µL

Tip Touch Delay: 300 ms

Tip Touch Speed: 70 µL/s

Trailing Air Gap Volume: 10 µL

Override the technique height by moving to =ZAsp mm from the bottom.

Dispense up to 1 time(s) per draw.

Create 1 replicate(s) of each source well.

Keep the current tips

Keep the tips when finished.

Stop when finished with Destinations.

Probes specified by "=OneTip()(MFX.NumSamples)" will be used.

Script

Description:

Labware Place to Cryo1

Execute the following script code:

```
Option Explicit

' This script duplicates the functionality of the Move Labware step.

' It has been modified to only place the labware after another step has picked it up.

Dim e

Set e = CreateObject("World.EngineObject")

Dim Target, PodName

Dim Pod, CurDeck, OrigPodSpeed, LWSpeedLimit


' PodName        The name of the pod to use

PodName = StepDictionary.Let.PodName

if IsEmpty(PodName) or PodName = "" then err.raise 1050, , "PodName must be defined"

Set Pod = World.Devices.Pipettor1(PodName)

OrigPodSpeed = Pod.CurrentSpeed


Set CurDeck = World.Devices.Pipettor1.Deck


' Target         Name of the target position (string)

' Need to use Deck.FindLabwarePosition,

' in case labware is referred to by name instead of location

Target = CurDeck.FindLabwarePosition(e.EvaluateExpression(StepDictionary.Let.Target))


if IsEmpty(Target) or Target = "" then err.raise 1050, , "Target must be defined"


If Not IsObject(Pod.GrippedLabware) Then err.raise 1050, , "There is no labware in the
gripper."

Dim glw
```

```
set glw = Pod.GrippedLabware(Pod.GrippedLabware.Count - 1) ' The gripped labware is an
array with the bottom piece at the highest index

' Convert Source and Target names into objects
Dim Dst
Set Dst = Pipettor.Deck.Positions(Target)

Dim Z
Dim sx, sy, sz
' Dim gx, gy, gz, pd
'pd = CDbl(Pod.VariantDictionary.GetDefault("GripperDOffset", 0.0))

' Get the squeeze values
'Dim Unsqueeze
'Unsqueeze = CDbl(Pod.DAxisFromGripper(CDbl(pd) + CDbl(glw.Class.Unsqueeze)))

' Get the Pod gripper offsets
Dim px, py, pz
px = CDbl(Pod.GripperXOffset)
py = CDbl(Pod.GripperYOffset)
pz = CDbl(Pod.GripperZOffset)

' Enforce the labware's speed limit
'LWSpeedLimit = srcLabware.Class.SpeedLimit
'If LWSpeedLimit < OrigPodSpeed Then Pod.SetSpeed LWSpeedLimit

'Get height of destination position
Z = CDbl(Dst.Z(Pod))
```

```
' Get offsets from position to the labware at target position
Dim tx, ty, tz, dx, dy, dz
tx = CDbl(Dst.GetOneLabwareOffsetAtDepth(1, "X"))
ty = CDbl(Dst.GetOneLabwareOffsetAtDepth(1, "Y"))
tz = CDbl(Dst.GetOneLabwareOffsetAtDepth(1, "Z"))

' Get the Target Labware
Dim dstLabware
dx = 0
dy = 0
dz = 0
dstLabware = Null
if (Dst.StackDepth > 0) then
  Set dstLabware = Dst.GetLabwareAtDepth(1)
end if

' Get Destination Stack Offsets (really Per-Labware Offsets)
dx = CDbl(Dst.GetOneStackOffset(glw, dstLabware, "X"))
dy = CDbl(Dst.GetOneStackOffset(glw, dstLabware, "Y"))
dz = CDbl(Dst.GetOneStackOffset(glw, dstLabware, "Z"))

' Get the labware gripper offsets (added-in by MAG 3/7/2003)
World.Volatile.MyDest = Dst

'Dim gx, gy, gz, pd
'gx = CDbl(glw.Class.GripperXOffset)
'gy = CDbl(glw.Class.GripperYOffset)
'gz = CDbl(glw.Class.GripperZOffset)
```

```
pd = CDbl(Pod.VariantDictionary.GetDefault("GripperDOffset", 0.0))

' Get the labware gripper offsets and squeeze values
Dim gx, gy, gz, pd
Dim Unsqueeze
Select Case Pod.PodType
   Case "ATTILA"
      gx = CDbl(glw.Class.GripperInfo.MultiChannel.GripperXOffset)
      gy = CDbl(glw.Class.GripperInfo.MultiChannel.GripperYOffset)
      gz = CDbl(glw.Class.GripperInfo.MultiChannel.GripperZOffset)
      Unsqueeze = CDbl(Pod.DAxisFromGripper(CDbl(pd) +
CDbl(glw.Class.GripperInfo.MultiChannel.Unsqueeze)))
'tls.msg pod.podtype
'tls.msg gx & " " & gy & " " & gz & " "  & " " & Unsqueeze
   Case Else ' need to fill in code for NX Gripper
End Select

' Open Destination
Dst.Open True, 0

' Start Moving
Pod.ApproachPosition Target, CDbl(tx+dx+gx-px), CDbl(ty+dy+gy-py), CDbl(Z + tz+dz+gz-pz),
True

Pod.MoveZ CDbl(Z + tz + dz + gz - pz)
```

```
' Put Labware

Pod.AbsMove ,,,Unsqueeze


' Notify software the labware that was in the grippers is now at the target

' old v. 2.x syntax Pod.PutLabware Target

Pod.Gripper.PutLabwareObject Target


' Retract grippers

Pod.GripperRetract


' Close the destination position

Dst.Close True, 0


'Restore original pod speed now that the move is finished

'Pod.SetSpeed OrigPodSpeed


' Move to a safe height

Pod.MoveToSafe

---------------------------------------------------------------------------

End Scripted Let

---------------------------------------------------------------------------

Run parkinwashstation

  rule = 3

  withtipwash = False

---------------------------------------------------------------------------

End

---------------------------------------------------------------------------
```

Else

End

If

If "IsRunning AND MFX.P7UseShaker":

Then

Group

Device Action

Send the following command to "OrbitalShakerALP0": "Shake".

Parameters: =MFX.P7ShakerSpeed, 1, Clockwise

Run waittimer

  index = =""

  settimer = True

  timername = ="Shake Timer"

  waittime = ="00:00:" & MFX.P7ShakeSeconds/2

Device Action

Send the following command to "OrbitalShakerALP0": "Stop".

Parameters: 1

End Group

Group

Device Action

Send the following command to "OrbitalShakerALP0": "Shake".

Parameters: =MFX.P7ShakerSpeed, 1, CounterClockwise

Run waittimer

  index = =""

  settimer = True

  timername = ="Shake Timer"

  waittime = ="00:00:" & MFX.P7ShakeSeconds/2

Device Action

Send the following command to "OrbitalShakerALP0": "Stop".

Parameters: 1

End Group

End

Else

End

Run waittimer

  index = =""

  settimer = True

  timername = ="Pausing..."

  waittime = =MFX.P7PauseTime

```
If

If "TLS.SecondsLeft("Incubating...") < MFX.P7ShakeSeconds + MFX.P7PauseSeconds":

---------------------------------------------------------------------------

Then

---------------------------------------------------------------------------

Break out of 1 loop(s).

---------------------------------------------------------------------------

End

---------------------------------------------------------------------------

Else

---------------------------------------------------------------------------

End

---------------------------------------------------------------------------

End Loop

---------------------------------------------------------------------------

Run waittimer

  index = =""

  settimer = False

  timername = ="Incubating..."

  waittime = ""

---------------------------------------------------------------------------

End Group

---------------------------------------------------------------------------

Run droptips

---------------------------------------------------------------------------

Run setstepdone

  wait = true

---------------------------------------------------------------------------

End Scripted Let
```

```
--------------------------------------------------------------------------

End

--------------------------------------------------------------------------

Else

--------------------------------------------------------------------------

End

--------------------------------------------------------------------------

End Group

--------------------------------------------------------------------------

Group

--------------------------------------------------------------------------

If

If "MFX.NextStep = 21":

--------------------------------------------------------------------------

Then

--------------------------------------------------------------------------

Move Labware

Move the top "2" plates at "Cryo1" to "Magnet" using pod "Pod1".

--------------------------------------------------------------------------

Move To

Move "Pod1" to deck position "P2" at offset (0,0).

--------------------------------------------------------------------------

Run setstepdone

  wait = true

--------------------------------------------------------------------------

End

--------------------------------------------------------------------------

Else
```

```
--------------------------------------------------------------------------
End
--------------------------------------------------------------------------
If
If "MFX.NextStep = 22":
--------------------------------------------------------------------------
Then
--------------------------------------------------------------------------
Run waittimer
  index = =""
  settimer = True
  timername = ="Settlling..."
  waittime = =SettleTime
--------------------------------------------------------------------------
Run setstepdone
  wait = true
--------------------------------------------------------------------------
End
--------------------------------------------------------------------------
Else
--------------------------------------------------------------------------
End
--------------------------------------------------------------------------
If
If "MFX.NextStep = 23":
--------------------------------------------------------------------------
Then
--------------------------------------------------------------------------
Move Labware
```

Move the top "1" plates at "Cryo1" to "TR2" using pod "Pod1".

Run setstepdone

  wait = true

End

Else

End

If

If "MFX.NextStep = 24":

Then

Run combinecolumns

  transfervolume = = (MFX.P7VolDAB + MFX.P7VolMedia) * 1.1

Run setstepdone

  wait = true

End

Else

End

If

If "MFX.NextStep = 25":

Then

Move Labware

Move the top "1" plates at "Cryo1" to "Orbital1" using pod "Pod1".

Move To

Move "Pod1" to deck position "P2" at offset (0,0).

Run setstepdone

  wait = true

End

Else

End

If

If "MFX.NextStep = 26":

Then

Run getnewtips

  pattern = =TwoTips()(MFX.NumSamples)

Transfer

Using Pod2, execute the following transfer:

From: Buffer, with the following pattern:

```
   1
 A ()
 B ()
 C ()
 D ()
 E ()
 F ><
```

, Media

Proceed down first, then left to right.

Start from the beginning of the selection.

Set the mark at the last well transferred.

Use the following custom technique:

Use the following pipetting template: OS Span-8 MultiDispense Slow Retract 2

Calibration Offset: 2.54

Calibration Slope: 1.033

Minimum Pipetting Height: 0.25 mm

Prewet: False

Aspirate Blowout: True

Follow Liquid: False

Height: 0 mm from the liquid

Mix: False

Mix Aspirate Speed: 250µL/s

Mix Aspirate Height: 1 mm from the liquid

Mix Dispense Speed: 250µL/s

Mix Dispense Height: 25 mm from the liquid

Mix Count: 1

Mix Volume: 800 µL

Operation speed: 70µL/s

Tip Touch: False

Trailing Air Gap: True

Override Liquid Type Settings

Aspirate Delay: 10 ms

Aspirate Speed: 300 µL/s

Blowout Delay: 20 ms

Blowout Volume: 10 µL

Dispense Delay: 10 ms

Dispense Speed: 200 µL/s

Prewet Delay: 0 ms

Prewet Overage: 0 µL

Tip Touch Delay: 300 ms

Tip Touch Speed: 85 µL/s

Trailing Air Gap Volume: 0 µL

Override the technique height by moving to 1 mm from the bottom.

To: Cryo1, =MFX.P8VolMediaµL, sections specified by "=Wells()(MFX.NumSamples)", Media

Proceed down first, then left to right.

Start from the beginning of the selection.

Set the mark at the last well transferred.

Use the following custom technique:

Use the following pipetting template: OS Span-8 P1000 Blowout at LiqLevel 2

Calibration Offset: 2.54

Calibration Slope: 1.033

Minimum Pipetting Height: =0.5 mm

Prewet: False

Blowout: True

Follow Liquid: True

Height: 0 mm from the liquid

Mix: False

Mix Aspirate Speed: 100µL/s

Mix Aspirate Height: 2 mm from the bottom

Mix Dispense Speed: 100µL/s

Mix Dispense Height: 0.5 mm from the bottom

Mix Count: 1

Mix Volume: 10 µL

Operation speed: 70µL/s

Tip Touch: False

Override Liquid Type Settings

Aspirate Delay: 10 ms

Aspirate Speed: 30 µL/s

Blowout Delay: 10 ms

Blowout Volume: 10 µL

Dispense Delay: 10 ms

Dispense Speed: 200 µL/s

Prewet Delay: 0 ms

Prewet Overage: 0 µL

Tip Touch Delay: 300 ms

Tip Touch Speed: 85 µL/s

Trailing Air Gap Volume: 0 µL

Override the technique height by moving to 1 mm from the bottom.

Aspirate at most =MaxVolInTips µL per transfer for repeated dispensing.

Create 1 replicate(s) of each source well.

Keep the current tips

Keep the tips when finished.

Stop when finished with Destinations.

Probes specified by "=TwoTips()(MFX.NumSamples)" will be used.

Run parkinwashstation

  withtipwash = False

Run setstepdone

  wait = true

End

Else

End

If

If "MFX.NextStep = 27":

Then

Device Action

Send the following command to "OrbitalShakerALP0": "Shake".

Parameters: =MFX.P8ShakerSpeed, 1, CounterClockwise

Run waittimer

  index = =""

  settimer = True

  timername = ="Shaking..."

  waittime = =MFX.P8ShakeTime

Device Action

Send the following command to "OrbitalShakerALP0": "Stop".

Parameters: 1

Run setstepdone

  wait = true

End

Else

End

If

If "MFX.NextStep = 28":

Then

Move Labware

Move the top "1" plates at "Cryo1" to "Magnet" using pod "Pod1".

Move To

Move "Pod1" to deck position "P2" at offset (0,0).

Run setstepdone

  wait = true

```
--------------------------------------------------------------------------

End

--------------------------------------------------------------------------

Else

--------------------------------------------------------------------------

End

--------------------------------------------------------------------------

If

If "MFX.NextStep = 29":

--------------------------------------------------------------------------

Then

--------------------------------------------------------------------------

Run waittimer

  index = =""

  settimer = True

  timername = ="Settlling..."

  waittime = =SettleTime

--------------------------------------------------------------------------

Run setstepdone

  wait = true

--------------------------------------------------------------------------

End

--------------------------------------------------------------------------

Else

--------------------------------------------------------------------------

End

--------------------------------------------------------------------------

If

If "MFX.NextStep = 30":
```

```
--------------------------------------------------------------------------

Then

--------------------------------------------------------------------------

Run combinecolumns

  transfervolume = = MFX.P8VolMedia * 1.1

--------------------------------------------------------------------------

Run setstepdone

  wait = true

--------------------------------------------------------------------------

End

--------------------------------------------------------------------------

Else

--------------------------------------------------------------------------

End

--------------------------------------------------------------------------

If

If "MFX.NextStep = 31":

--------------------------------------------------------------------------

Then

--------------------------------------------------------------------------

Move Labware

Move the top "1" plates at "Cryo1" to "P7" using pod "Pod1".

--------------------------------------------------------------------------

Run setstepdone

  wait = true

--------------------------------------------------------------------------

End

--------------------------------------------------------------------------
```

Else

End

If

If "MFX.NextStep = 32":

Then

Move Labware

Move the top "1" plates at "Cryo2" to "Magnet" using pod "Pod1".

Move To

Move "Pod1" to deck position "P2" at offset (0,0).

Run setstepdone

  wait = true

End

Else

End

If

If "MFX.NextStep = 33":

Then

```
Run waittimer

  index = =""

  settimer = True

  timername = ="Settlling..."

  waittime = =SettleTime

--------------------------------------------------------------------------

Run setstepdone

  wait = true

--------------------------------------------------------------------------

End

--------------------------------------------------------------------------

Else

--------------------------------------------------------------------------

End

--------------------------------------------------------------------------

If

If "MFX.NextStep = 34":

--------------------------------------------------------------------------

Then

--------------------------------------------------------------------------

Run getnewtips

  pattern = =OneTip()(MFX.NumSamples)

--------------------------------------------------------------------------

Loop

Loop from "transfer" = "5" to "6", incrementing by "1".

--------------------------------------------------------------------------

Transfer Samples to Fresh Vials (Configure Step)

Bitmap: C:\Program Files\Biomek Software\Bitmaps\Vials.bmp
```

Description:

Caption: Transfer Samples to Fresh Vials (Configure Step)

Code: ' Note: each well in each src column contains 2*(MFX.P7VolDAB + MFX.P7VolMedia + MFX.P8VolMedia))

```
' but since we are doing two transfers the volume in each step is only half that

transvol = (MFX.P7VolDAB + MFX.P7VolMedia + MFX.P8VolMedia) * 1.1

Set LW = Positions(Labware.Location("Cryo2")).Labware
LW.ConfigureAmounts transvol

' volumes for the destination columns (5 & 6) need to be set to 0
LW.SetWellAmounts Array(0,0,0,0,0,0,0,0), Split(Col56,","), LeftPod

nTrips = TLS.NumTrips(transvol, MaxVolInTips)

Extend "NumTrips", nTrips
Extend "VolTrip",  transvol/nTrips
```

Prompt: [IDispatch]

Tooltip: Scripted Let

Loop

Loop from "Trip" = "1" to "=NumTrips", incrementing by "1".

Calculate Disp Height

Bitmap: C:\Program Files\Biomek Software\Bitmaps\Vials.bmp

Description:

Caption: Calculate Disp Height

Code: Set LW = Positions(Labware.Location("Cryo2")).Labware

VolBeforeDisp = VolTrip *(Trip-1)

DispHeightCM = LW.HeightFromVolume(VolBeforeDisp, 1)

' new in 0.25: start dispensing at 2 mm below liqlevel

' then follow up & blowout at dispheight

Extend "ZDisp", (DispHeightCM * 10) - 2

'if isrunning then

'PG.stalluntiletsis 0

'tls.msg (DispHeightCM * 10) - 2

'end if

Prompt: [IDispatch]

Tooltip: Scripted Let

Combine

Using Pod2, execute the following transfer:

From: Cryo2, =VolTripµL, sections specified by "=Col()(1)(MFX.NumSamples)", Water

Proceed down first, then left to right.

Start from the beginning of the selection.

Set the mark at the last well transferred.

Use the following custom technique:

  Use the following pipetting template: OS Span-8 P1000 SlowRetract 2

  Calibration Offset: 2.54

  Calibration Slope: 1.033

Minimum Pipetting Height: 0 mm

Prewet: False

Aspirate Blowout: True

Follow Liquid: True

Height: -2 mm from the liquid

Mix: False

Mix Aspirate Speed: 100µL/s

Mix Aspirate Height: 0 mm from the liquid

Mix Dispense Speed: 100µL/s

Mix Dispense Height: 0 mm from the liquid

Mix Count: 1

Mix Volume: 10 µL

Operation speed: 50µL/s

Tip Touch: False

Trailing Air Gap: True

Override Liquid Type Settings

- Aspirate Delay: 10 ms
- Aspirate Speed: 100 µL/s
- Blowout Delay: 0 ms
- Blowout Volume: 10 µL
- Dispense Delay: 0 ms
- Dispense Speed: 200 µL/s
- Prewet Delay: 0 ms
- Prewet Overage: 0 µL
- Tip Touch Delay: 0 ms
- Tip Touch Speed: 50 µL/s
- Trailing Air Gap Volume: 1 µL

Override the technique height by moving to 0.5 mm from the bottom.

From: Cryo2, =VolTripµL, sections specified by "=Col()(2)(MFX.NumSamples)", Water

Proceed down first, then left to right.

Start from the beginning of the selection.

Set the mark at the last well transferred.

Use the following custom technique:

Use the following pipetting template: OS Span-8 P1000 Blowout at LiqLevel 2

Calibration Offset: 2.54

Calibration Slope: 1.033

Minimum Pipetting Height: 1.5 mm

Prewet: False

Aspirate Blowout: True

Follow Liquid: True

Height: -2 mm from the liquid

Mix: False

Mix Aspirate Speed: 100µL/s

Mix Aspirate Height: 0 mm from the liquid

Mix Dispense Speed: 100µL/s

Mix Dispense Height: 0 mm from the liquid

Mix Count: 1

Mix Volume: 10 µL

Operation speed: 50µL/s

Tip Touch: False

Trailing Air Gap: True

Override Liquid Type Settings

Aspirate Delay: 10 ms

Aspirate Speed: 50 µL/s

Blowout Delay: 0 ms

Blowout Volume: 5 µL

Dispense Delay: 0 ms

Dispense Speed: 50 µL/s

Prewet Delay: 0 ms

Prewet Overage: 0 µL

Tip Touch Delay: 0 ms

Tip Touch Speed: 50 µL/s

Trailing Air Gap Volume: 1 µL

Override the technique height by moving to 0.5 mm from the bottom.

From: Cryo2, =VolTripµL, sections specified by "=Col()(3)(MFX.NumSamples)", Water

Proceed down first, then left to right.

Start from the beginning of the selection.

Set the mark at the last well transferred.

Use the following custom technique:

Use the following pipetting template: OS Span-8 P1000 SlowRetract 2

Calibration Offset: 2.54

Calibration Slope: 1.033

Minimum Pipetting Height: 1.5 mm

Prewet: False

Aspirate Blowout: True

Follow Liquid: True

Height: -2 mm from the liquid

Mix: False

Mix Aspirate Speed: 100µL/s

Mix Aspirate Height: 0 mm from the liquid

Mix Dispense Speed: 100µL/s

Mix Dispense Height: 0 mm from the liquid

Mix Count: 1

Mix Volume: 10 µL

Operation speed: 50µL/s

Tip Touch: False

Trailing Air Gap: True

Override Liquid Type Settings

Aspirate Delay: 10 ms

Aspirate Speed: 50 µL/s

Blowout Delay: 0 ms

Blowout Volume: 5 µL

Dispense Delay: 0 ms

Dispense Speed: 50 µL/s

Prewet Delay: 0 ms

Prewet Overage: 0 µL

Tip Touch Delay: 0 ms

Tip Touch Speed: 50 µL/s

Trailing Air Gap Volume: 1 µL

Override the technique height by moving to 0.5 mm from the bottom.

To: Cryo2, sections specified by "=Col()(transfer)(MFX.NumSamples)", Water

Proceed down first, then left to right.

Start from the beginning of the selection.

Set the mark at the last well transferred.

Use the following custom technique:

Use the following pipetting template: OS Span-8 P1000 Blowout at LiqLevel 2

Calibration Offset: 2.54

Calibration Slope: 1.033

Minimum Pipetting Height: 0.5 mm

Prewet: False

Blowout: True

Follow Liquid: True

Height: 1.5 mm from the bottom

Mix: False

Mix Aspirate Speed: 100µL/s

Mix Aspirate Height: 0 mm from the liquid

Mix Dispense Speed: 100µL/s

Mix Dispense Height: 0 mm from the liquid

Mix Count: 1

Mix Volume: 10 µL

Operation speed: 60µL/s

Tip Touch: True

Override Liquid Type Settings

Aspirate Delay: 10 ms

Aspirate Speed: 50 µL/s

Blowout Delay: 0 ms

Blowout Volume: 5 µL

Dispense Delay: 0 ms

Dispense Speed: 50 µL/s

Prewet Delay: 0 ms

Prewet Overage: 0 µL

Tip Touch Delay: 0 ms

Tip Touch Speed: 50 µL/s

Trailing Air Gap Volume: 1 µL

Override the technique height by moving to =ZDisp mm from the bottom.

Dispense up to 1 time(s) per draw.

Create 1 replicate(s) of each source well.

Keep the current tips

Keep the tips when finished.

```
Stop when finished with Sources.

Probes specified by "=OneTip()(MFX.NumSamples)" will be used.

---------------------------------------------------------------------------

End Scripted Let

---------------------------------------------------------------------------

End Loop

---------------------------------------------------------------------------

End Scripted Let

---------------------------------------------------------------------------

End Loop

---------------------------------------------------------------------------

Run droptips

---------------------------------------------------------------------------

Run setstepdone

  wait = true

---------------------------------------------------------------------------

End

---------------------------------------------------------------------------

Else

---------------------------------------------------------------------------

End

---------------------------------------------------------------------------

If

If "MFX.NextStep = 35":

---------------------------------------------------------------------------

Then

---------------------------------------------------------------------------

Move Labware
```

Move the top "1" plates at "Cryo2" to "Orbital1" using pod "Pod1".

Move To

Move "Pod1" to deck position "P2" at offset (0,0).

Run setstepdone

  wait = true

End

Else

End

If

If "MFX.NextStep = 36":

Then

Run getnewtips

  pattern = =OneTip()(MFX.NumSamples)

Transfer

Using Pod2, execute the following transfer:

From: Buffer, with the following pattern:

```
   1
 A ()
 B ()
 C ()
```

D ()

E ()

F ><

, Media

Proceed down first, then left to right.

Start from the beginning of the selection.

Set the mark at the last well transferred.

Use the following custom technique:

Use the following pipetting template: OS Span-8 MultiDispense Slow Retract 2

Calibration Offset: 2.54

Calibration Slope: 1.033

Minimum Pipetting Height: 0.25 mm

Prewet: False

Aspirate Blowout: True

Follow Liquid: False

Height: 0 mm from the liquid

Mix: False

Mix Aspirate Speed: 250µL/s

Mix Aspirate Height: 1 mm from the liquid

Mix Dispense Speed: 250µL/s

Mix Dispense Height: 25 mm from the liquid

Mix Count: 1

Mix Volume: 800 µL

Operation speed: 70µL/s

Tip Touch: False

Trailing Air Gap: True

Override Liquid Type Settings

Aspirate Delay: 10 ms

Aspirate Speed: 300 µL/s

Blowout Delay: 20 ms

Blowout Volume: 10 µL

Dispense Delay: 10 ms

Dispense Speed: 200 µL/s

Prewet Delay: 0 ms

Prewet Overage: 0 µL

Tip Touch Delay: 300 ms

Tip Touch Speed: 85 µL/s

Trailing Air Gap Volume: 0 µL

Override the technique height by moving to 1 mm from the bottom.

To: Cryo2, =MFX.P8VolMediaµL, sections specified by "=Col()(1)(MFX.NumSamples)", Media

Proceed down first, then left to right.

Start from the beginning of the selection.

Set the mark at the last well transferred.

Use the following custom technique:

Use the following pipetting template: OS Span-8 P1000 Blowout at LiqLevel 2

Calibration Offset: 2.54

Calibration Slope: 1.033

Minimum Pipetting Height: =0.5 mm

Prewet: False

Blowout: True

Follow Liquid: False

Height: 0 mm from the liquid

Mix: False

Mix Aspirate Speed: 100µL/s

Mix Aspirate Height: 2 mm from the bottom

Mix Dispense Speed: 100µL/s

Mix Dispense Height: 0.5 mm from the bottom

Mix Count: 1

Mix Volume: 10 µL

Operation speed: 50µL/s

Tip Touch: False

Override Liquid Type Settings

Aspirate Delay: 10 ms

Aspirate Speed: 30 µL/s

Blowout Delay: 10 ms

Blowout Volume: 10 µL

Dispense Delay: 10 ms

Dispense Speed: 200 µL/s

Prewet Delay: 0 ms

Prewet Overage: 0 µL

Tip Touch Delay: 300 ms

Tip Touch Speed: 85 µL/s

Trailing Air Gap Volume: 0 µL

Override the technique height by moving to 2 mm from the bottom.

To: Cryo2, =MFX.P8VolMediaµL, sections specified by "=Col()(2)(MFX.NumSamples)", Water

Proceed down first, then left to right.

Start from the beginning of the selection.

Set the mark at the last well transferred.

Use the following custom technique:

Use the following pipetting template: Span-8 MultiDispense

Calibration Offset: 2.54

Calibration Slope: 1.033

Minimum Pipetting Height: 1.5 mm

Prewet: False

Blowout: True

Follow Liquid: False

Height: 1.5 mm from the bottom

Mix: False

Mix Aspirate Speed: 100µL/s

Mix Aspirate Height: 0 mm from the liquid

Mix Dispense Speed: 100µL/s

Mix Dispense Height: 0 mm from the liquid

Mix Count: 1

Mix Volume: 10 µL

Operation speed: 50µL/s

Tip Touch: False

Override Liquid Type Settings

Aspirate Delay: 10 ms

Aspirate Speed: 50 µL/s

Blowout Delay: 0 ms

Blowout Volume: 5 µL

Dispense Delay: 0 ms

Dispense Speed: 50 µL/s

Prewet Delay: 0 ms

Prewet Overage: 0 µL

Tip Touch Delay: 0 ms

Tip Touch Speed: 50 µL/s

Trailing Air Gap Volume: 1 µL

Override the technique height by moving to 2 mm from the bottom.

To: Cryo2, =MFX.P8VolMediaµL, sections specified by "=Col()(3)(MFX.NumSamples)", Water

Proceed down first, then left to right.

Start from the beginning of the selection.

Set the mark at the last well transferred.

Use the following custom technique:

Use the following pipetting template: Span-8 MultiDispense

Calibration Offset: 2.54

Calibration Slope: 1.033

Minimum Pipetting Height: 1.5 mm

Prewet: False

Blowout: True

Follow Liquid: False

Height: 1.5 mm from the bottom

Mix: False

Mix Aspirate Speed: 100µL/s

Mix Aspirate Height: 0 mm from the liquid

Mix Dispense Speed: 100µL/s

Mix Dispense Height: 0 mm from the liquid

Mix Count: 1

Mix Volume: 10 µL

Operation speed: 50µL/s

Tip Touch: False

Override Liquid Type Settings

Aspirate Delay: 10 ms

Aspirate Speed: 50 µL/s

Blowout Delay: 0 ms

Blowout Volume: 5 µL

Dispense Delay: 0 ms

Dispense Speed: 50 µL/s

Prewet Delay: 0 ms

    Prewet Overage: 0 µL

    Tip Touch Delay: 0 ms

    Tip Touch Speed: 50 µL/s

    Trailing Air Gap Volume: 1 µL

Override the technique height by moving to 2 mm from the bottom.

Aspirate at most =MaxVolInTips µL per transfer for repeated dispensing.

Create 1 replicate(s) of each source well.

Keep the current tips

Keep the tips when finished.

Stop when finished with Destinations.

Probes specified by "=OneTip()(MFX.NumSamples)" will be used.

Run parkinwashstation

  withtipwash = False

Run setstepdone

  wait = true

End

Else

End

If

If "MFX.NextStep = 37":

Then

Device Action

Send the following command to "OrbitalShakerALP0": "Shake".

Parameters: =MFX.P8ShakerSpeed, 1, CounterClockwise

Run waittimer

index = =""

settimer = True

timername = ="Shaking..."

waittime = =MFX.P8ShakeTime

Device Action

Send the following command to "OrbitalShakerALP0": "Stop".

Parameters: 1

Run setstepdone

wait = true

End

Else

End

If

If "MFX.NextStep = 38":

Then

Move Labware

Move the top "1" plates at "Cryo2" to "Magnet" using pod "Pod1".

Move To

Move "Pod1" to deck position "P2" at offset (0,0).

Run setstepdone

  wait = true

End

Else

End

If

If "MFX.NextStep = 39":

Then

Run waittimer

  index = =""

  settimer = True

  timername = ="Settlling..."

  waittime = =SettleTime

Run setstepdone

  wait = true

End

Else

End

If

If "MFX.NextStep = 40":

Then

Run getnewtips

  pattern = =OneTip()(MFX.NumSamples)

Loop

Loop from "transfer" = "6" to "5", incrementing by "-1".

Transfer Samples to Fresh Vials (Configure Step)

Bitmap: C:\Program Files\Biomek Software\Bitmaps\Vials.bmp

Description:

Caption: Transfer Samples to Fresh Vials (Configure Step)

```
Code: ' this time transvol is the volume in the vial divided by two because
' we are doing two trips (cols. 5 and 6)

transvol = (MFX.P8VolMedia * 1.1)/2
Positions(Labware.Location("Cryo2")).Labware.ConfigureAmounts transvol
```

```
nTrips = TLS.NumTrips(transvol, MaxVolInTips)


Extend "NumTrips", nTrips

Extend "VolTrip",  transvol/nTrips




Prompt: [IDispatch]

Tooltip: Scripted Let

---------------------------------------------------------------------------

Loop

Loop from "Trip" = "1" to "=NumTrips", incrementing by "1".

---------------------------------------------------------------------------

Calculate Disp Height

Bitmap: C:\Program Files\Biomek Software\Bitmaps\Vials.bmp

Description:

Caption: Calculate Disp Height

Code: ' what is already in the wells in col 5/6 from the first transfer?

VolInWell =  2* (MFX.P7VolDAB + MFX.P7VolMedia + MFX.P8VolMedia)


' now add the trip volume

VolBeforeDisp = VolInWell + (VolTrip *(Trip-1))


Set LW = Positions(Labware.Location("Cryo2")).Labware

DispHeightCM = LW.HeightFromVolume(VolBeforeDisp, 1)


' new in 0.25: start dispensing at 2 mm below liqlevel

' then follow up & blowout at dispheight
```

```
Extend "ZDisp", (DispHeightCM * 10) - 2



'if isrunning then
'PG.stalluntiletsis 0
'tls.msg (DispHeightCM * 10) - 2
'end if
Prompt: [IDispatch]
Tooltip: Scripted Let
---------------------------------------------------------------------------
Combine
Using Pod2, execute the following transfer:
From: Cryo2, =VolTripµL, sections specified by "=Col()(1)(MFX.NumSamples)", Water
Proceed down first, then left to right.
Start from the beginning of the selection.
Set the mark at the last well transferred.
Use the following custom technique:
  Use the following pipetting template: OS Span-8 P1000 SlowRetract 2
  Calibration Offset: 2.54
  Calibration Slope: 1.033
  Minimum Pipetting Height: 0 mm
  Prewet: False
  Aspirate Blowout: True
  Follow Liquid: True
  Height: -2 mm from the liquid
  Mix: False
  Mix Aspirate Speed: 100µL/s
  Mix Aspirate Height: 0 mm from the liquid
```

Mix Dispense Speed: 100µL/s

Mix Dispense Height: 0 mm from the liquid

Mix Count: 1

Mix Volume: 10 µL

Operation speed: 50µL/s

Tip Touch: False

Trailing Air Gap: True

Override Liquid Type Settings

Aspirate Delay: 10 ms

Aspirate Speed: 100 µL/s

Blowout Delay: 0 ms

Blowout Volume: 10 µL

Dispense Delay: 0 ms

Dispense Speed: 200 µL/s

Prewet Delay: 0 ms

Prewet Overage: 0 µL

Tip Touch Delay: 0 ms

Tip Touch Speed: 50 µL/s

Trailing Air Gap Volume: 1 µL

Override the technique height by moving to 0.5 mm from the bottom.

From: Cryo2, =VolTripµL, sections specified by "=Col()(2)(MFX.NumSamples)", Water

Proceed down first, then left to right.

Start from the beginning of the selection.

Set the mark at the last well transferred.

Use the following custom technique:

Use the following pipetting template: OS Span-8 P1000 Blowout at LiqLevel 2

Calibration Offset: 2.54

Calibration Slope: 1.033

Minimum Pipetting Height: 1.5 mm

Prewet: False

Aspirate Blowout: True

Follow Liquid: True

Height: -2 mm from the liquid

Mix: False

Mix Aspirate Speed: 100µL/s

Mix Aspirate Height: 0 mm from the liquid

Mix Dispense Speed: 100µL/s

Mix Dispense Height: 0 mm from the liquid

Mix Count: 1

Mix Volume: 10 µL

Operation speed: 50µL/s

Tip Touch: False

Trailing Air Gap: True

Override Liquid Type Settings

- Aspirate Delay: 10 ms
- Aspirate Speed: 50 µL/s
- Blowout Delay: 0 ms
- Blowout Volume: 5 µL
- Dispense Delay: 0 ms
- Dispense Speed: 50 µL/s
- Prewet Delay: 0 ms
- Prewet Overage: 0 µL
- Tip Touch Delay: 0 ms
- Tip Touch Speed: 50 µL/s
- Trailing Air Gap Volume: 1 µL

Override the technique height by moving to 0.5 mm from the bottom.

From: Cryo2, =VolTripµL, sections specified by "=Col()(3)(MFX.NumSamples)", Water

Proceed down first, then left to right.

Start from the beginning of the selection.

Set the mark at the last well transferred.

Use the following custom technique:

Use the following pipetting template: OS Span-8 P1000 SlowRetract 2

Calibration Offset: 2.54

Calibration Slope: 1.033

Minimum Pipetting Height: 1.5 mm

Prewet: False

Aspirate Blowout: True

Follow Liquid: True

Height: -2 mm from the liquid

Mix: False

Mix Aspirate Speed: 100µL/s

Mix Aspirate Height: 0 mm from the liquid

Mix Dispense Speed: 100µL/s

Mix Dispense Height: 0 mm from the liquid

Mix Count: 1

Mix Volume: 10 µL

Operation speed: 50µL/s

Tip Touch: False

Trailing Air Gap: True

Override Liquid Type Settings

Aspirate Delay: 10 ms

Aspirate Speed: 50 µL/s

Blowout Delay: 0 ms

Blowout Volume: 5 µL

Dispense Delay: 0 ms

Dispense Speed: 50 µL/s

Prewet Delay: 0 ms

Prewet Overage: 0 µL

Tip Touch Delay: 0 ms

Tip Touch Speed: 50 µL/s

Trailing Air Gap Volume: 1 µL

Override the technique height by moving to 0.5 mm from the bottom.

To: Cryo2, sections specified by "=Col()(transfer)(MFX.NumSamples)", Water

Proceed down first, then left to right.

Start from the beginning of the selection.

Set the mark at the last well transferred.

Use the following custom technique:

Use the following pipetting template: OS Span-8 P1000 Blowout at LiqLevel 2

Calibration Offset: 2.54

Calibration Slope: 1.033

Minimum Pipetting Height: 0.5 mm

Prewet: False

Blowout: True

Follow Liquid: True

Height: 1.5 mm from the bottom

Mix: False

Mix Aspirate Speed: 100µL/s

Mix Aspirate Height: 0 mm from the liquid

Mix Dispense Speed: 100µL/s

Mix Dispense Height: 0 mm from the liquid

Mix Count: 1

Mix Volume: 10 µL

Operation speed: 50µL/s

Tip Touch: True

Override Liquid Type Settings

Aspirate Delay: 10 ms

Aspirate Speed: 50 µL/s

Blowout Delay: 0 ms

Blowout Volume: 5 µL

Dispense Delay: 0 ms

Dispense Speed: 50 µL/s

Prewet Delay: 0 ms

Prewet Overage: 0 µL

Tip Touch Delay: 0 ms

Tip Touch Speed: 50 µL/s

Trailing Air Gap Volume: 1 µL

Override the technique height by moving to =ZDisp mm from the bottom.

Dispense up to 1 time(s) per draw.

Create 1 replicate(s) of each source well.

Keep the current tips

Keep the tips when finished.

Stop when finished with Sources.

Probes specified by "=OneTip()(MFX.NumSamples)" will be used.

End Scripted Let

End Loop

End Scripted Let

End Loop

Run droptips

Run setstepdone

  wait = true

End

Else

End

If

If "MFX.UsePeltier":

Then

Incubate Pelt1 at 20C for 00:00:01

Position: Pelt1

Command: Incubate

Module Name:

Set Temperature?: -1

Temperature: 20

Total Time: 00:00:01

End

Else

End

End Group

End Group

End Scripted Let

Finish

Method completed.

Remove the tips from all pods. Clear all labware from the deck. Clear all labware from SILAS devices.Clear all global variables.
