# Supplementary figures and images for: Novel Automated Blood Separations Validate Whole Cell Biomarkers
Source: PLoS One. 2011 Jul 22;6(7):e22430. doi: 10.1371/journal.pone.0022430 (PMC3142167; doi:10.1371/journal.pone.0022430)

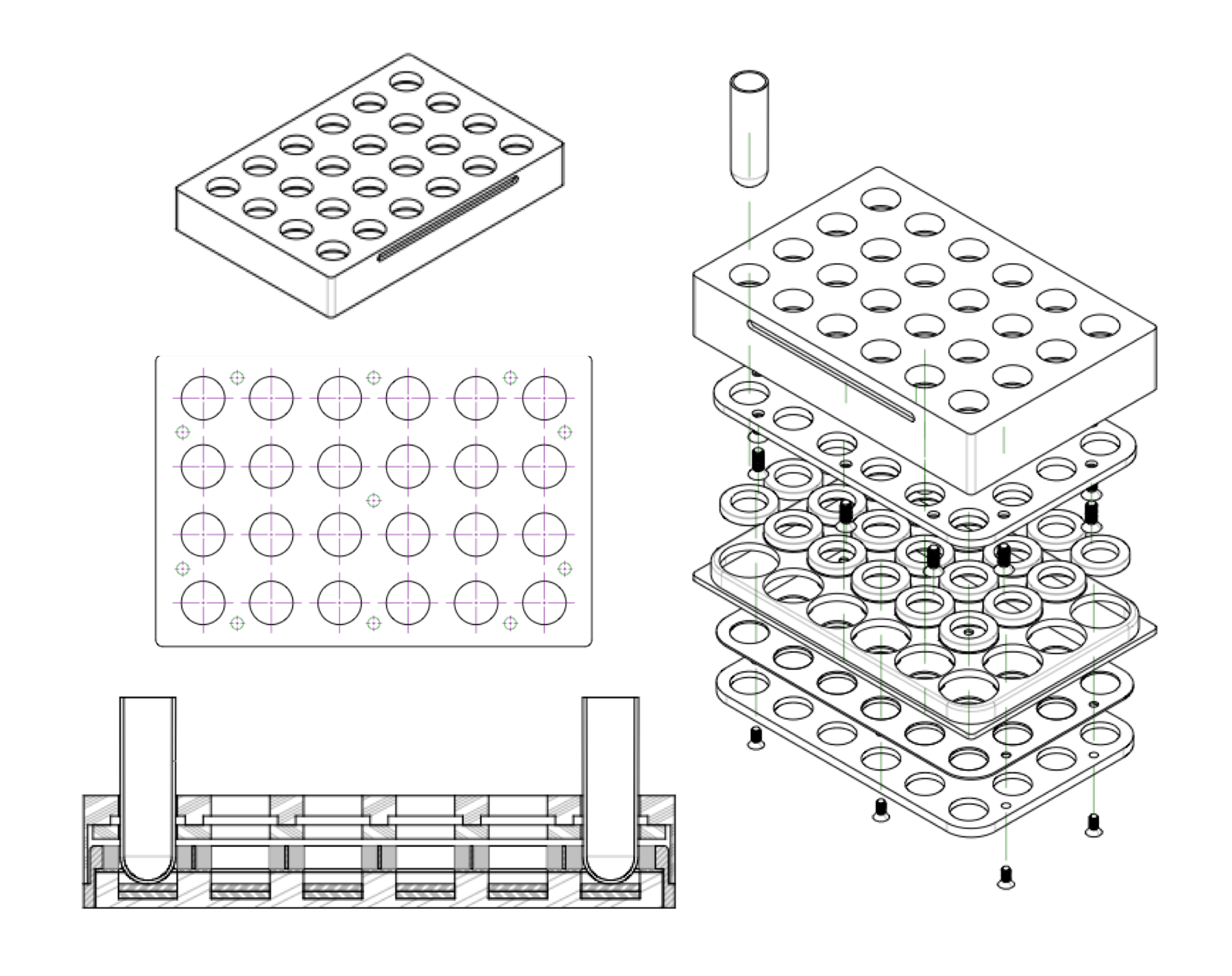

Supplement: Document S2 — Custom sample tube holders. We custom designed and built sample holders for 2 ml sample vials (up to 24 vials) and a maximum of four patient samples (one for each row). A sample occupies one-row of the four-row (by 6 column) holder design. Each (sample) row holds six vials to provide sufficient volume (up to 12 ml) to provide a sufficient number of cells of interest to be collected. Shown are several different views…clockwise from top left, are tube holder top view, viewing the tube holder at an angle, and a side view of both the sample plate with two (of 24) vials sitting within the holder and on the magnetic separation plate. The exploded view shows the various engineering components used to fabricate the sample holder base and magnetic separation plate. This plate is used to pull any magnetic beads that are present in the sample vial solutions to the sidewall. The plate design includes the use of 24 separate (rare earth) ring magnets (one ring magnet for each sample). One (2 ml) sample vial is illustrated above the sample holder. The use of multiple plate layers ensures correct ring magnet placement to allow for accurate sample vial x, y, z insertion position. The various cut-away plates also help to confine (isolate) magnetic field lines to minimize unwanted interferences and field line overflow at the bottom of the sample vials. Separately, a sample plate holder is shown (top-right) with inserted sample vial. The slot seen near the top (matching on opposite side) is used by the robotic arm to move the plate on/off the magnet plate. On the bottom right is a cut-away side view of a sample holder sitting on a magnet separation plate with the sample vial positioned in the ring magnet of an assembled magnet separation plate. As shown, the ring magnet is positioned near the bottom of the sample vial which allows use of large and small liquid volumes. (TIF) [file pone.0022430.s002.tif]
